# *Candida albicans* biofilm–induced vesicles confer drug resistance through matrix biogenesis

**DOI:** 10.1371/journal.pbio.2006872

**Published:** 2018-10-08

**Authors:** Robert Zarnowski, Hiram Sanchez, Antonio S. Covelli, Eddie Dominguez, Anna Jaromin, Jörg Bernhardt, Kaitlin F. Mitchell, Christian Heiss, Parastoo Azadi, Aaron Mitchell, David R. Andes

**Affiliations:** 1 Department of Medicine, Section of Infectious Diseases, University of Wisconsin–Madison, Madison, Wisconsin, United States of America; 2 Department of Medical Microbiology and Immunology, University of Wisconsin–Madison, Madison, Wisconsin, United States of America; 3 Department of Lipids and Liposomes, Faculty of Biotechnology, University of Wroclaw, Wroclaw, Poland; 4 Universitat Institute for Microbiology, Ernst Moritz Arndt University Greifswald, Greifswald, Germany; 5 Complex Carbohydrate Research Center, University of Georgia, Athens, Georgia, United States of America; 6 Department of Biological Sciences, Carnegie Mellon University, Pittsburgh, Pennsylvania, United States of America; University of Iowa, United States of America

## Abstract

Cells from all kingdoms of life produce extracellular vesicles (EVs). Their cargo is protected from the environment by the surrounding lipid bilayer. EVs from many organisms have been shown to function in cell–cell communication, relaying signals that impact metazoan development, microbial quorum sensing, and pathogenic host–microbe interactions. Here, we have investigated the production and functional activities of EVs in a surface-associated microbial community or biofilm of the fungal pathogen *Candida albicans*. Crowded communities like biofilms are a context in which EVs are likely to function. Biofilms are noteworthy because they are encased in an extracellular polymeric matrix and because biofilm cells exhibit extreme tolerance to antimicrobial compounds. We found that biofilm EVs are distinct from those produced by free-living planktonic cells and display strong parallels in composition to biofilm matrix material. The functions of biofilm EVs were delineated with a panel of mutants defective in orthologs of endosomal sorting complexes required for transport (ESCRT) subunits, which are required for normal EV production in diverse eukaryotes. Most ESCRT-defective mutations caused reduced biofilm EV production, reduced matrix polysaccharide levels, and greatly increased sensitivity to the antifungal drug fluconazole. Matrix accumulation and drug hypersensitivity of ESCRT mutants were reversed by addition of wild-type (WT) biofilm EVs. Vesicle complementation showed that biofilm EV function derives from specific cargo proteins. Our studies indicate that *C*. *albicans* biofilm EVs have a pivotal role in matrix production and biofilm drug resistance. Biofilm matrix synthesis is a community enterprise; prior studies of mixed cell biofilms have demonstrated extracellular complementation. Therefore, EVs function not only in cell–cell communication but also in the sharing of microbial community resources.

## Author summary

*Candida albicans*—the most common fungal pathogen in humans—often grows as a biofilm, resulting in an infection that is difficult to treat. These adherent communities tolerate extraordinarily high concentrations of antifungals due in large part to the protective extracellular matrix. The present study identifies extracellular vesicles (EVs) that are distinct to biofilms. These EVs deliver the functional extracellular matrix and are essential for resistance to antifungals. Our findings not only reveal a coordinated mechanism by which the defining trait of the biofilm lifestyle arises but also identify a number of potential therapeutic targets.

## Introduction

Vesicles are released externally by cells of bacteria, archaea, and eukaryotes [1–3]. These extracellular vesicles (EVs) deliver cargo of RNA and protein that is protected by a surrounding lipid bilayer. Classes of EVs have been distinguished based upon their size, cargo, and mechanisms of biogenesis [1–3]. Functional analysis has shown that EVs play diverse biological roles in delivery of effectors to target cells. For example, during *Drosophila* wing development, secretion of the morphogenic effector Hedgehog in EVs is required for activation of many of its target genes [4]. For many bacterial pathogens, toxin delivery via EVs causes host cell damage or lysis [1]. In the case of the eukaryotic protozoan *Trypanosoma brucei*, EVs orchestrate community escape from sources of environmental stress [5]. The purpose of EV secretion is thus tailored to each organism's biology and environmental context.

Microorganisms exist predominantly in surface-associated communities called biofilms, which typically have high cell density and include an extracellular polymeric matrix [6]. Biofilm cells are notorious for their resistance to antimicrobial treatments [7], a property often determined by multiple mechanisms [8]. Our interest is in the eukaryotic microorganism *Candida albicans*, which poses a severe threat to hospitalized patients with vascular devices due to its capacity for biofilm formation [9, 10]. *Candida* species proliferate on the surface of these devices as a biofilm [11–13]. *Candida* biofilm cells resist available drug therapies [14], and thus, the only currently effective therapy is removal of medical devices, which is often impossible for critically ill patients [15]. One of the central determinants of *C*. *albicans* (mating type locus [MTL] a/α) biofilm drug resistance is a mannan–glucan complex in the extracellular matrix [16, 17]. Our findings reported here show that EVs promote assembly of the mannan–glucan complex that leads to drug resistance. We suggest that drug resistance of other microbial biofilms may also rely upon the efficient sharing of community resources as EV cargo.

## Results/Discussion

### Production of distinctive biofilm EVs

**Fig 1:**
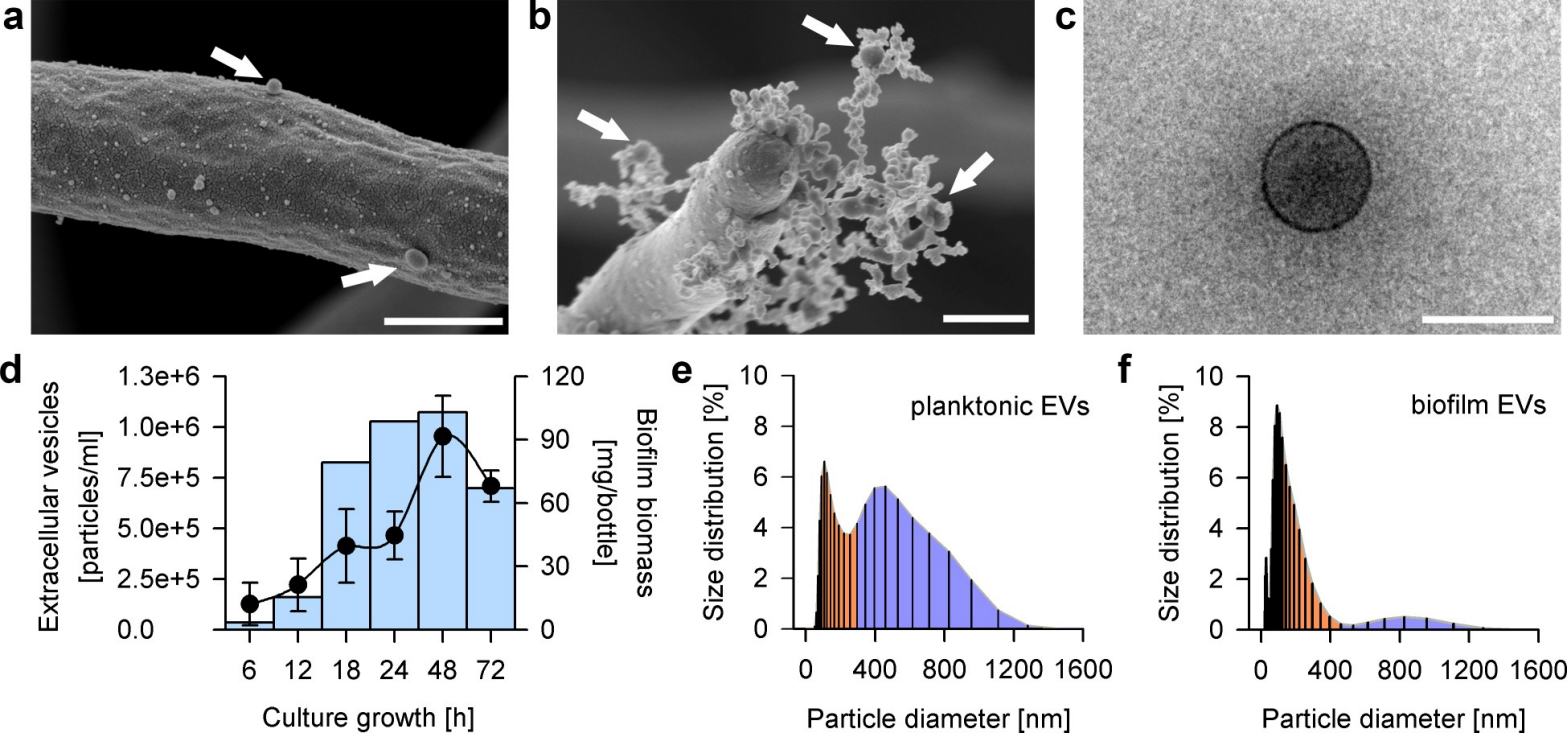
*C*. *albicans* biofilms secrete unique EVs. (a) A SEM of EV-like structures on the surface of *C*. *albicans* growing in a biofilm. Scale bar indicates 0.6 μm. (b) A SEM of EV-like structures within deposits of the extracellular matrix in biofilms. Scale bar indicates 0.5 μm. (c) A cryoTEM of *Candida* biofilm-derived EVs are surrounded by a 7-nm-thick lipid bilayer. Scale bar indicates 100 nm. (d) Quantitative analysis of EVs in *C*. *albicans* biofilms measured at various culture growth time points using imaging flow cytometry. Biofilm cell number was quantified by dry weight. The measurements were done in triplicate. (e) Size distribution of *C*. *albicans* planktonic EVs evaluated by dynamic light scattering. (f) Size distribution of *C*. *albicans* biofilm EVs evaluated by dynamic light scattering. Underlying data can be found in S1 Data. EV, extracellular vesicle; SEM, scanning electron micrograph.

We have reported that *C*. *albicans* biofilm extracellular matrix includes a significant phospholipid component [18], a finding that might indicate the presence of EVs in the matrix material. In support of this idea, we observed numerous <100-nm spheres on the surface of biofilm cells (Fig 1A) and embedded in the extracellular matrix (Fig 1B). EVs, isolated from biofilm [19, 20] and imaged by cryoTEM, were enriched for an exosome population based upon size [21] (Fig 1C), though other vesicle types may be included in the preparation. Time course studies revealed that vesicle production peaks at 48 h after biofilm initiation (Fig 1D). These kinetics paralleled the time course of both biofilm cell accumulation and matrix deposition [22]. Our results indicate that *C*. *albicans*, like many other microbes [1, 23], produces biofilm EVs.

EVs are known to be produced by free-living planktonic cells of numerous fungi, including *C*. *albicans* [1, 24, 25]. We assessed the similarity of biofilm and planktonic EVs through comparisons of their sizes and composition. The present observations with *C*. *albicans* are consistent with studies of *Saccharomyces cerevisiae* [26] revealing the production of two populations of planktonic EVs (Fig 1E). There is a 30–200-nm diameter population that corresponds in size to exosomes and a larger 200–1,000-nm diameter population that corresponds in size to microvesicles [26]. In contrast, biofilm EVs comprise predominantly a 30–200-nm diameter exosome-sized population (Fig 1F).

**Fig 2:**
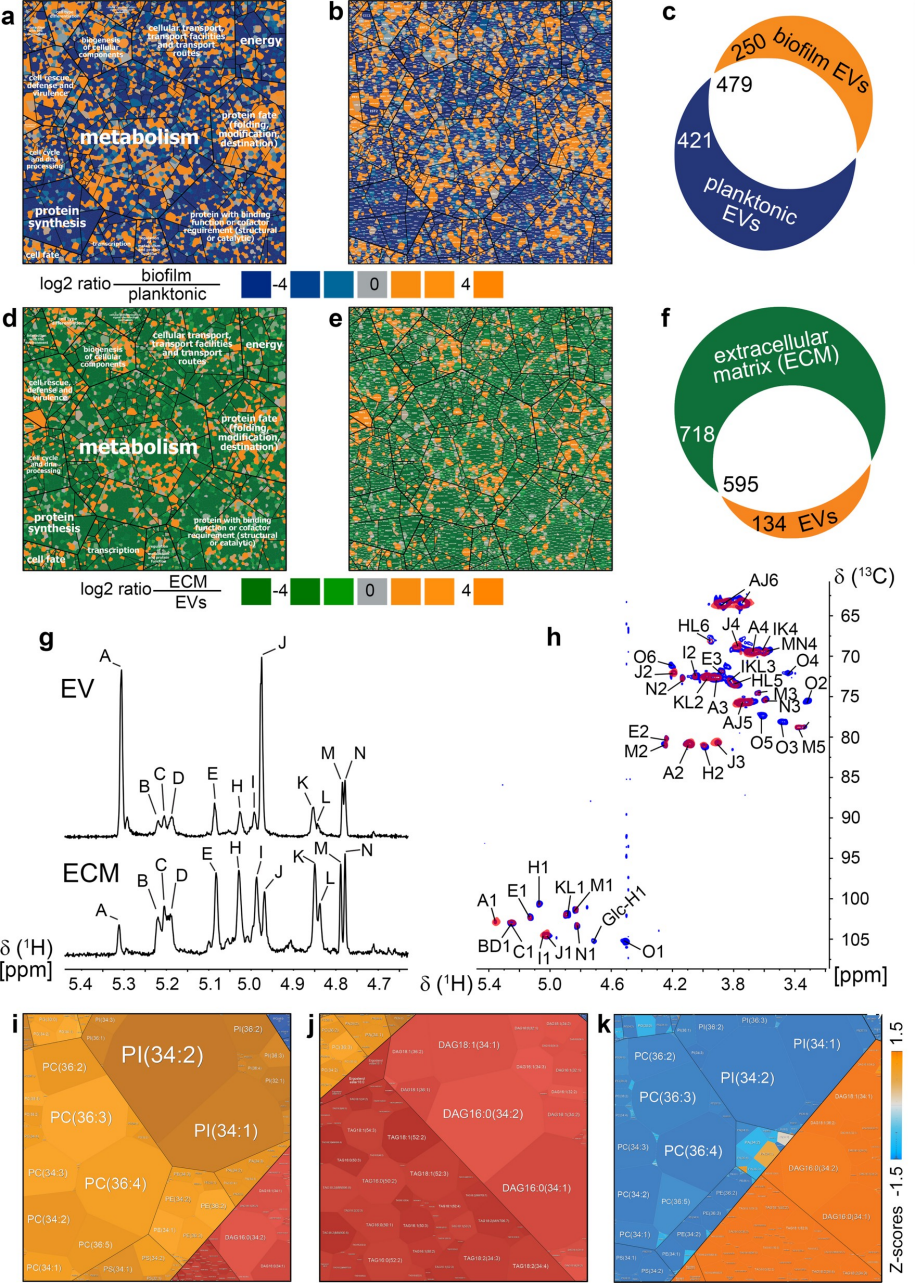
Unique *C*. *albicans* biofilm EV protein, lipid, and carbohydrate cargo delivers biofilm extracellular matrix components. Biofilm EVs proteomes are shown in orange versus planktonic EVs shown in blue (a, b, c), whereas ECM is shown in green (d, e, f). Smallest regions (a, b, d, e) represent identified proteins and are arranged inside higher level regions according to their KEGG functional category and pathway assignment by using a Voronoi treemap layout. (a, b) Log2 biofilm EVs/planktonic EVs ratios of proteins relative abundances were mapped to a color ramp starting with orange (more protein in biofilm EVs), passing grey (similar protein proportions in biofilm EVs as well as planktonic EVs), and reaching blue (more protein in planktonic cells). (c) The number of exclusive (blue and orange) and common (white) biofilm EVs and planktonic EVs proteins are illustrated by using a Venn diagram. (d, e, f) Accordingly, the proteome comparisons of biofilm EVs (orange) and ECM (green) are shown. (I) Lipidomics profiles in biofilm EVs and the ECM. The treemaps reflect relative amounts of individual lipid species present in EVs or ECM. The coloration of individual clusters based on their classification showing phospholipids in orange, neutral lipids in red, and sphingolipids in blue. Quantitative differences of biofilm EVs lipids and extracellular matrix lipids are given by using z-scores. Blue illustrates lipids with higher concentrations in biofilm EVs, whereas orange reflects lipids more abundant in ECM. ECM, extracellular matrix; EV, extracellular vesicle; KEGG, Kyoto Encyclopedia of Genes and Genomes.

Proteomic analysis revealed that planktonic and biofilm EVs have a considerable proportion of distinct cargo, with 34% of the proteome being unique to the biofilm state (Fig 2A–2C and S1 Table). In addition, many proteins shared by vesicles from both sources were 10- to 100-fold more abundant in the biofilm EVs. Our results indicate that EVs produced by biofilms are distinct from those of planktonic cells.

The composition of biofilm EVs pointed toward two prospective roles in biofilm extracellular matrix biogenesis. First, vesicle composition shows a high degree of similarity with matrix composition protein (Fig 2D–2F) and polysaccharide content (Fig 2G and 2H), suggesting that vesicles may be a major source of matrix material. The protein comparison suggests that up to 45% of the proteins in the biofilm matrix may be delivered by vesicles (Fig 2F and S2 Table). Polysaccharide analysis revealed a predominance of mannan and glucan, two major matrix components, in vesicle cargo by gas chromatography, which identified both components in a percent ratio of 84.0 ± 1.6/3.2 ± 1.0 in vesicles and 44.3 ± 4.2/8.8 ± 1.2 in the matrix, respectively. The major mannan component of the complex displayed structural similarity to the biofilm matrix mannan–glucan complex by ^1^H NMR in Fig 2G and S3 Table and 2D ^1^H-^13^C NMR in Fig 2H and S4 Table, a determinant of biofilm associated drug resistance [27]. Thus, biofilm vesicles may deliver cargo that forms the extracellular matrix. Comparative analysis of the lipid composition of biofilm EVs and matrix revealed similarity in the sphingolipid and phospholipid components, particularly in phosphatidylcholine, phosphatidylinositol, and phosphatidylethanolamine (Fig 2I). However, the neutral lipid component in the extracellular matrix appeared distinct and likely reflects an additional vesicle-independent mechanism of delivery for the remaining lipid constituents.

A second possible role is that vesicle cargo has a catalytic function in matrix macromolecule synthesis. Specifically, one of the enriched functional ontology categories for the biofilm EV proteome was polysaccharide modification (Fig 2A–2F). These observations suggest that biofilm EVs may deposit cargo that contributes directly to matrix structure, and they may also provide catalytic activities that engage in matrix polysaccharide synthesis.

### Role of biofilm EVs in matrix production

**Fig 3:**
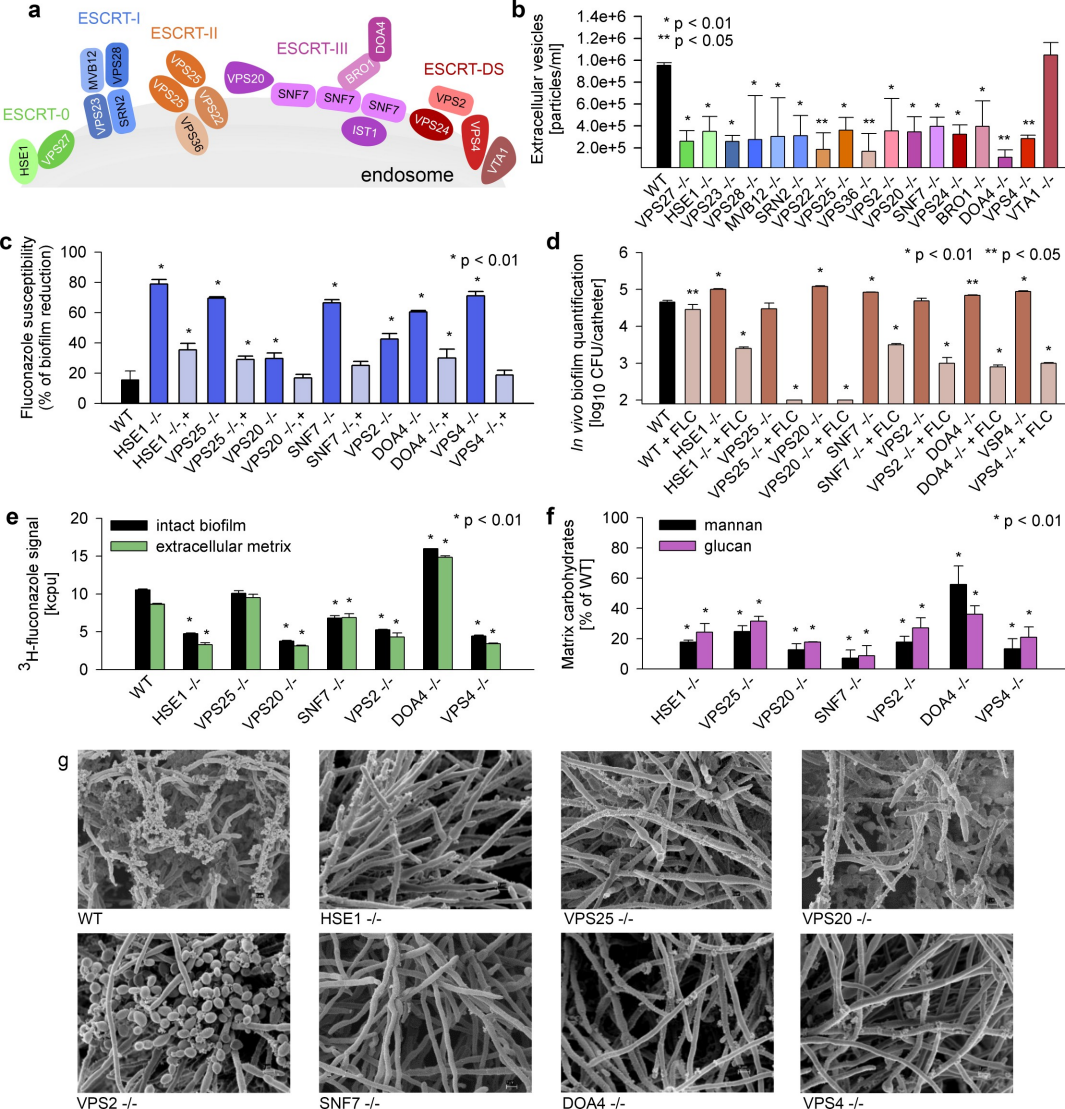
ESCRT driven biofilm EVs are responsible for drug resistance due to delivery of macromolecules to the *C*. *albicans* extracellular matrix. (a) A diagram of the ESCRT machinery involved in sorting cargo via EVs. (b) Quantitative analysis of biofilm EVs in the *C*. *albicans* WT strain and the ESCRT null mutants assessed by the imaging flow cytometry system. The experiment and assays were done in triplicate and data presented as particles per ml. Bars indicate standard deviation of the median. (c) The percent of reduction in biofilm formation following 48-h treatment with fluconazole (1 mg/ml) compared with untreated biofilms, as quantified using the 96-well XTT assay. The null deletions and corresponding complemented strains are shown for mutants with fluconazole susceptibility phenotype. The experiments and assays were performed in triplicate. Asterisks indicate values significantly different from the reference strain based on one-way ANOVA with the posthoc Tukey HSD test. Bars indicate standard deviation of the median. (d) Quantification of in vivo biofilms using a rat central venous catheter model. Individual fluconazole-susceptible ESCRT null mutants were treated either with fluconazole 250 μg/ml or 0.9 M NaCl followed by the CFU analysis. Three animal and culture replicates per condition. (e) Sequestration of ^3^H-labelled fluconazole by intact biofilms grown from the reference and ESCRT mutant strains. Biofilms were exposed to the radiolabeled drug, washed, and harvested. Scintillation counting was performed in triplicate to determine the fluconazole content in the intact biofilms and the isolated matrix. (f) The percent reduction of mannan and glucan concentration in biofilm matrices of fluconazole-susceptible ESCRT null mutants measured by gas chromatography. Three biological and assay replicates per data point. (g) Impact of ESCRT null mutants on biofilm architecture and extracellular matrix based upon SEM imaging of mature (24 h) in vitro biofilms. Underlying data can be found in S1 Data. CFU, colony-forming unit; ESCRT, endosomal sorting complexes required for transport; EV, extracellular vesicle; H, hydrogen; HSD, honestly significant difference; NaCl, sodium chloride; SEM, scanning electron micrograph; WT, wild-type.

We sought to test our hypothesis that biofilm EVs function in matrix biogenesis. The size range of biofilm EVs suggests that they are exosomes [21], and in other eukaryotes, exosome production is governed by the endosomal ESCRT pathway [21]. In fact, we note that biofilm vesicle cargo includes ESCRT subunits Hse1 and Vps27 (S1 Table). We identified 21 *C*. *albicans* ESCRT subunit homologs to *S*. *cerevisiae* and created homozygous deletion mutants (Fig 3A and 3B). Sixteen of the mutants showed decreased vesicle production (Fig 3B). We note that exosome production depends upon only a subset of ESCRT subunits in other eukaryotes [3, 21] in keeping with our observations for *C*. *albicans*. The ESCRT mutants with reduced EV production enabled us to test whether biofilm vesicles have a role in biofilm matrix biogenesis and function.

We screened the ESCRT vesicle–defective mutants for biofilm matrix–associated phenotypes. All mutants produced a biofilm structure, but a subset had prominent defects. Seven of the ESCRT mutants exhibited hypersusceptibility to the antifungal fluconazole during biofilm growth (Fig 3C). The enhanced susceptibility biofilm phenotype was reversed in each of these ESCRT mutants for which a WT allele was introduced (despite multiple attempts, we did not successfully construct a VPS2 complemented strain). This change in drug susceptibility was biofilm specific, as planktonic susceptibility was similar in WT and these ESCRT mutants (MIC range 0.25–0.5 μg/ml). The clinical relevance of these observations was confirmed via demonstration of congruent drug-susceptibility phenotypes in the rat vascular catheter biofilm model [28] (Fig 3D). Our previous studies have shown that biofilm matrix sequesters antifungals to promote drug resistance [16, 17, 29], and we verified that the six of the seven drug-susceptible ESCRT mutants were also defective in fluconazole sequestration (Fig 3E). We speculate that the sole ESCRT mutant that did not exhibit altered drug sequestration (DOA4) may reflect a difference in vesicle cargo or perhaps a matrix-independent resistance mechanism. Drug sequestration has been linked to matrix quantity and presence of a mannan–glucan complex (MGCx). Each of the ESCRT mutants with vesicle and drug-susceptibility defects similarly displayed defects in matrix mannan and glucan quantity (Fig 3F). As the vesicles alone did not sequester antifungals (S1 Fig), we reason this phenomenon is due to vesicle matrix delivery. These extracellular matrix defects are also demonstrated visually by the absence of matrix that adorns WT biofilms for each of the seven vesicle mutants (Fig 3G).

**Fig 4:**
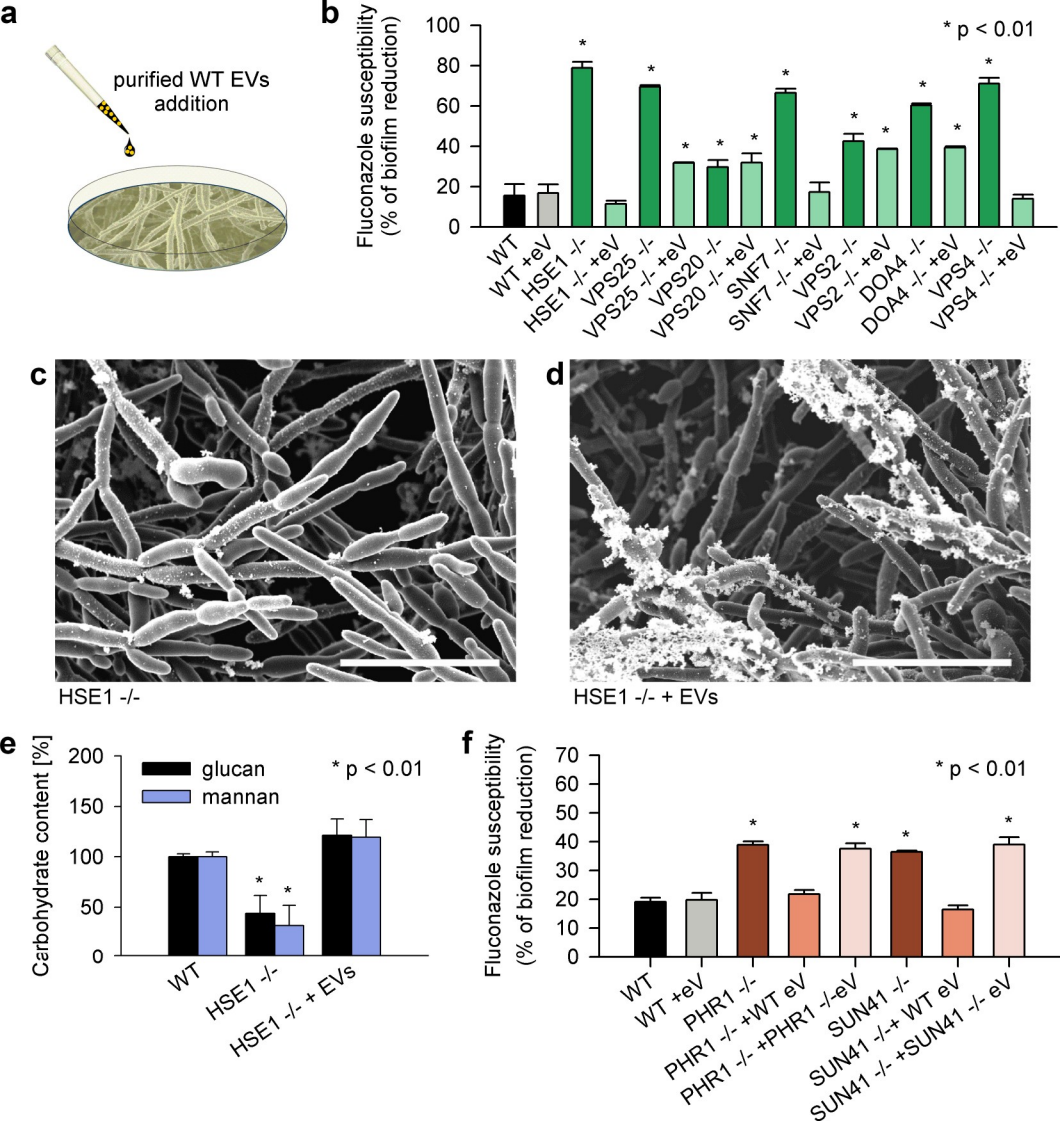
Exogenous delivery of WT vesicles restores the biofilm drug-resistant phenotype and matrix composition. (a) A diagram depicting the addition of purified WT EVs from *C*. *albicans* biofilm cultures to mutant biofilms. (b) Effect of exogenous WT biofilm EVs on biofilm fluconazole susceptibility for select ESCRT null mutants as measured by the 96-well XTT assay. Biofilm cultures of fluconazole-sensitive mutant strains amended with WT EVs (21,804 ± 1,711 EVs/ml) regain their ability to grow in the presence of fluconazole. Each experiment and assay was performed in triplicate. (c) Exogenous WT EVs rescue matrix production in ESCRT mutant biofilms. The fluconazole-susceptible biofilm of HSE1 null mutant does not produce extracellular matrix (upper SEM). The addition of exogenous vesicles restores the mutant’s ability to produce the extracellular matrix (lower SEM). Scale bars indicate 11 μm. (d) Exogenous EVs restore mannan and glucan concentrations in the biofilm matrix of HSE1 null mutant as measured by gas chromatography. Each experiment and assay was performed in triplicate. (e) Effect of exogenous biofilm EVs on drug susceptibility of select *C*. *albicans* matrix glucan-modification null mutants as measured by the 96-well XTT assay. The vesicle cargo mutants (PHR1, SUN41) regain their ability to grow in the presence of fluconazole after the addition of exogenous WT EVs. Each experiment and assay was performed in triplicate. Underlying data can be found in S1 Data. ESCRT, endosomal sorting complexes required for transport; EV, extracellular vesicle; SEM, scanning electron micrograph; SUN41, putative endo-beta-D-glucosidase; PHR1, putative glycanosyltransferase; WT, wild-type.

We considered two models for the relationship between ESCRT function, biofilm EVs, and matrix biogenesis. One model is that biofilm EVs have a direct role in matrix biogenesis; ESCRT defects cause matrix defects by reducing the levels of vesicles or packaging of functionally relevant cargo. An alternative model is that EVs have no role in matrix biogenesis; ESCRT defects cause matrix defects due to indirect effects. The second model stems from the growing appreciation that ESCRT machinery, with its central role in organelle physiology, has impact on diverse aspects of cell biology [30]. We used a “vesicle add-back” protocol to test these models (Fig 4A). Specifically, if vesicles have a direct role in matrix biogenesis, then providing WT biofilm vesicles to a vesicle-defective ESCRT mutant should restore matrix production and matrix-associated phenotypes. Remarkably, the addition of the WT vesicles to drug-susceptible ESCRT mutants increased drug resistance dramatically (Fig 4B). Furthermore, the addition of WT biofilm vesicles restored biofilm matrix architecture and quantities of the key mannan–glucan components (Fig 4C and 4D). These results support the first model: a subset of ESCRT subunits promote matrix biogenesis and function through their role in biofilm EV production.

### Biofilm EV function in cargo delivery

Among the proteins in biofilm EVs, several have previously defined roles in biofilm matrix biogenesis and specifically matrix polysaccharide modification (S2 Table) [16, 27]. We considered a model in which the presence of these proteins as vesicle cargo is central to their functional activity; they are “functional passengers.” An alternative model is that they are “coincidental passengers” in vesicles and that their true function is vesicle independent. For example, they may function in matrix biogenesis at intracellular sites or after conventional secretion into the extracellular milieu. We deployed our vesicle add-back protocol to test these models, using mutants in cargo proteins putative glycanosyltransferase (Phr1) and putative endo-beta-D-glucosidase (Sun41), which act in the glucan modification pathway (Fig 4E) [16, 31]. Remarkably, addition of WT vesicles to these drug susceptible cargo mutants restored drug resistance. Control studies in which vesicles from the Phr1 and Sun41 mutants were added to the respective mutants did not alter the fluconazole susceptibility phenotype. These results favor the functional passenger model—that cargo proteins function to confer biofilm drug resistance as vesicle components rather than through some vesicle-independent activity.

Our results indicate that biofilm growth of *C*. *albicans* results in a distinctive EV population and cargo. These findings echo studies of bacterial and eukaryotic cells that show that EV properties reflect environmental and developmental signals [1, 3]. Our findings also add a new facet to the understanding of EV function: whereas prior studies have shown a role for EVs in cell–cell signaling, our studies reveal a role for EVs in the sharing of community resources [27], that of biofilm matrix material. Matrix is a pivotal determinant of *C*. *albicans* biofilm drug resistance, and our results reveal EV-dependence for drug resistance both in vitro and in an animal biofilm infection model. Our findings suggest that EV-based therapeutics [32] may be a useful new platform for antibiofilm strategies.

## Materials and methods

### Ethics statement

All animal procedures were approved by the Institutional Animal Care and Use Committee at the University of Wisconsin according to the guidelines of the Animal Welfare Act, The Institute of Laboratory Animal Resources Guide for the Care and Use of Laboratory Animals, and Public Health Service Policy. The approved animal protocol number is DA0031.

### Fungal strain construction and growth conditions

The parent strain *C*. *albicans* SN152 (MTL a/α) was used to create homozygous deletion strains (S6 Table) using a SOE-PCR-based disruption cassette method, employing histidine and lysine auxotrophic markers [33]. PCR with primers listed in S5 Table was used to verify genotypes. Complementation of mutant strains with a single gene-of-interest copy used selection for arginine prototrophy. Transformants were selected on minimal medium with the corresponding auxotrophic supplements. Both planktonic and biofilm cultures were grown in RPMI 1640, buffered with 4-morpholinepropanesulfonic acid (MOPS) for all experiments described below [34].

### In vitro biofilm models

One of four in vitro biofilm models was used, including a 96-well or 6-well polystyrene plate, polystyrene roller bottle, or glass coverslip. Biofilm drug susceptibility was assessed using the 96-well flat-bottom polystyrene plate assay [35–38]. Matrix composition assessment utilized the 6-well plate assay. Biofilm architecture was imaged using scanning electron micrograph (SEM) using a coverslip biofilm assay. Matrix biochemistry was determined from biofilms growing, using a rolling bottle system [34]. A minimum of three biological replicates were performed for each assay.

### Matrix isolation from biofilms

Biofilm matrix for matrix biochemical analysis was grown using a rolling bottle biofilm model [34]. After 48 h of growth, media was removed, and the *Candida* biofilms were dislodged from the roller bottle surface by spatula. The intact biofilm was then gently sonicated to remove matrix from cells (sonication with a 6-mm microtip at 20 kHz with an amplitude of 30% for 8 min), followed by centrifugation to separate fungal cells from the matrix. The isolated matrix was then lyophilized. Matrix was similarly isolated from 6-well biofilm plates [16].

### Large-scale purification of EVs

EVs were isolated from both planktonic cultures and large-scale biofilms grown in polystyrene roller bottles [34]. The culture media was removed from the bottles, filter sterilized, and concentrated down to 25 ml using a Vivaflow 200 unit (Sartorius AG, Goettingen, Germany) equipped with a Hydrosart 30 kDa cut-off membrane. The sample was centrifuged at 10,000 × *g* for 1 h at 4°C to remove smaller cellular debris. The pellets were discarded, and the resulting supernatant was centrifuged again as described above. The resulting supernatant was then centrifuged at 100,000 × *g* for 1.5 h at 4°C. The supernatants were then discarded, and the pellet was then resuspended in phosphate-buffered saline (PBS) (pH 7.2). Next, the sample was subject to size exclusion chromatography on a HighPrep 16/60 Sephacryl S-400 HR column (GE Life Sciences) pre-equilibrated with PBS (pH 7.2) containing 0.01% NaN_3_. All chromatographic separation steps were performed at room temperature on the high-performance liquid chromatography ÄKTA-Purifier 10 system (Amersham Biosciences AB, Uppsala, Sweden).

### Quantitative vesicle analysis using imaging flow cytometry

EVs were quantified using a combination of imaging flow cytometry, image confirmation, and fluorescence sensitivity in low-background samples, as previously described [39, 40]. Prior to analysis, samples were stained with carboxyfluorescein succinimidyl ester (CSFE) and 1,1'-dioctadecyl-3,3,3',3'-tetramethylindocarbocyanine perchlorate (Dil) at 37°C for 90 min. Excessive dye particles were removed from stained vesicles using illustra microspin G-50 columns (GE Healthcare). All samples were analyzed on the ImageStreamX Mk II flow cytometry system from Amnis Corporation (Seattle, Washington, United States) at ×60 magnification, with default low flow rate/high sensitivity using the INSPIRE software.

### Measurements of EVs

The mean particle size of the vesicles dispersions were determined using a Zetasizer Nano-ZS (Malvern Instruments, Malvern, United Kingdom). In order to obtain the optimum light scattering intensity, 10 μl of the vesicles suspension was added to 990 μl of PBS. All the measurements were carried out in triplicate at 25°C [41].

### Imaging of EVs and biofilms

For SEM of biofilms, 40 μl of an inoculum of 10^8^ cells/ml in RPMI–MOPS was added to the coverslips and incubated for 60 min at 37°C. 1 ml RPMI–MOPS was added to each well, and the plates were incubated at 37°C for 20 h. One ml fixative (4% formaldehyde, 1% glutaraldehyde in PBS) was then added to each well prior to incubation at 4°C overnight. Coverslips were then washed with PBS prior to incubation for 30 min in 1% osmium tetroxide. Samples were then serially dehydrated in ethanol (30% to 100%). Critical point drying was used to completely dehydrate the samples prior to palladium-gold coating. Samples were imaged on a SEM LEO 1530, with Adobe Photoshop 7.0.1 used for image compilation [27].

For cryoTEM, 3 μl of sample suspensions were pipetted onto a glow-discharged 200 mesh copper grid with a lacey carbon support film (EMS, 1560 Industry Road, Hatfield, Pennsylvania, 19440, US, #LC200-CU). Before sample application, the grid was mounted on a tweezer in the Vitrobot (FEI, 5350 NE Dawson Creek Drive, Hillsboro, Oregon, 97124, US, model MarkIII). In an automated sequence, excess fluid was blotted off, and the grid was plunge frozen in liquid ethane. Once frozen, the grid was mounted in a precooled cryo transfer sample holder (Gatan, 780 Commonwealth Drive, Warrendale, Pennsylvania 15086, US, model 626) and inserted into the TEM (Hitachi Ltd., 4026, Kuji-cho, Hitachi-shi, Ibaraki, 319–12, Japan, model HT7700). The samples were observed at 120 kV acceleration voltage, and the sample temperature was kept at −170°C.

### Gel-free proteome analysis

Enzymatic “in liquid” digestion and mass spectrometric analysis was done at the Mass Spectrometry Facility, Biotechnology Center, University of Wisconsin–Madison. 200 μg of matrix proteins were extracted by precipitation with 15% TCA/60% acetone and then incubated at −20°C for 30 min. The matrix or vesicle preparation was centrifuged at 16,000 × *g* for 10 min, and the resulting pellets were washed twice with ice-cold acetone, followed by an ice-cold MeOH wash. Pelleted proteins were resolubilized and denatured in 10 μl of 8 M urea in 100 mM NH_4_HCO_3_ for 10 min, then diluted to 60 μl for tryptic digestion with the following reagents: 3 μl of 25 mM DTT, 4.5 μl of acetonitrile, 36.2 μl of 25 mM NH_4_HCO_3_, 0.3 μl of 1M Tris-HCl, and 6 μl of 100 ng/μl Trypsin Gold solution in 25 mM NH_4_HCO_3_ (Promega Co., Madison, WI). Digestion was conducted in two stages, first overnight at 37°C, then additional 4 μl of trypsin solution were added and the mixture was incubated at 42°C for an additional 2 h. The reaction was terminated by acidification with 2.5% TFA to a final concentration of 0.3% and then centrifuged at 16,000 × *g* for 10 min. Trypsin-generated peptides were analyzed by nanoLC-MS/MS using the Agilent 1100 nanoflow system (Agilent, Palo Alto, CA) connected to a hybrid linear ion trap-orbitrap mass spectrometer (LTQ-Orbitrap, Thermo Fisher Scientific, San Jose, CA) equipped with a nanoelectrospray ion source. Capillary HPLC was performed using an in-house fabricated column with an integrated electrospray emitter, as described elsewhere [42]. Sample loading and desalting were achieved using a trapping column in line with the autosampler (Zorbax 300SB-C18, 5 μm, 5 × 0.3 mm, Agilent). The LTQ-Orbitrap was set to acquire MS/MS spectra in a data-dependent mode as follows: MS survey scans from 300 to 2,000 m/z were collected in profile mode with a resolving power of 100,000. MS/MS spectra were collected on the five most abundant signals in each survey scan. Dynamic exclusion was employed to increase the dynamic range and maximize peptide identifications. Raw MS/MS data were searched against a concatenated *C*. *albicans* amino acid sequence database using an in-house MASCOT search engine [43]. Identified proteins were further annotated and filtered to 1.5% peptide and 0.1% protein false-discovery-rate with Scaffold Q+ version 3.0 (Proteome Software Inc., Portland, Oregon) using the protein prophet algorithm [44].

### Functional mapping of the EV and matrix proteomes

The *C*. *albicans* vesicle and matrix proteomes were analyzed using the Kyoto Encyclopedia of Genes and Genomes (KEGG) [45, 46]. Each protein predicted from the *C*. *albicans* genome assigned a KEGG Ontology ID (KOID) was obtained, and the specific pathway and superpathway membership information retained. This was then correlated with the experimental proteome data, and the number of proteins expressed within a given pathway was then determined. Tabulated proteins were presented as a percentage out of the total number of proteins predicted to belong to a given pathway from the *C*. *albicans* genome, as determined by KEGG. The visualization of relative quantities of biofilm proteins was also done using KEGG protein functional categorization. On the basis of this hierarchical classification scheme, Voronoi treemaps were constructed [47]. This approach divides screen space according to hierarchy levels in which the main functional categories determine screen sections on the first level, subsidiary categories on the second level, and so forth. The polygonic cells of the deepest level represented functionally classified proteins and were colored according to relative abundance of each protein that was determined based on total counts of corresponding trypsin-digested peptides.

### Isolation and analysis of EV and matrix lipids

Lipids were extracted from the desalted lyophilized EV or matrix powder with a mixture of CHCl_3_/MeOH (2:1, by vol) containing 0.1 g/l BHT. The sample was vortexed, incubated in the dark for 2 h at room temperature, and then centrifuged. The separated layer of organic solvents was removed, and the pellet was washed with 2 ml of CHCl_3_/MeOH (2:1, by vol) and centrifuged. The collected lipid extracts were combined and dried under a stream of nitrogen. After drying, the sample was reconstituted in 0.5 ml of CHCl_3_/MeOH (2:1, by vol.) and subjected to TLC separation on 20 cm × 20 cm silica gel Si60 plates. Neutral lipids were separated in hexane/ethyl ether/AcOH (90:20:1, by vol), which yielded triacylglycerols, sterol esters, free fatty acids, and a pool of immobile phospholipids. The latter group was scrapped off the plate, extracted from the silica gel, and subjected to another TLC separation in CHCl_3_/MeOH/AcOH/H_2_O (50:37.5:3.5:2, by vol). This step yielded four classes of glycerolipids (phosphatidylcholine, phosphatidylethanolamine, phosphatidylserine, and phosphatidylinositol) and one class of sphingolipids (sphingomyelins). Lipids were visualized under UV light after spraying plates evenly with a 0.2% solution of fluorescein in EtOH. All isolated lipid classes were scraped off their silica gel plates and re-extracted with CHCl_3_/MeOH (4:1, by vol) containing 0.1g/l BHT. Samples were vortexed, incubated overnight at room temperature, and then centrifuged in order to remove silica gel particles. 100 μl of 0.05 mg/ml pentadecanoic acid was added to each sample and the organic solvents were evaporated under nitrogen. Next, isolated lipids were subjected to methylation in the presence of 0.5 ml of 14% BF_3_ in MeOH. Vials containing the processed lipids were boiled. After cooling, the samples were mixed with 1 ml hexane and 0.5 ml H_2_O, vortexed, and centrifuged. The top hexane layer containing methyl ester derivatives was transferred to a new clean glass tube, dried under nitrogen, resuspended in 100 μl hexane, and transferred to GC vials. Fatty acid methyl esters were identified by gas chromatography using a Hewlett-Packard 5890 equipped with a capillary column coated with DB-225 (30-m length, 0.25-mm internal diameter, 0.25 μm; Agilent Technologies, Inc., Wilmington, Delaware). Peaks were identified by a comparison of retention times with a set of authentic fatty acid standards provided by Supelco. The abundance of fatty acids was calculated from the relative peak areas [18].

### Isolation and purification of vesicle and matrix carbohydrates

Delipidated vesicle and matrix pellets containing carbohydrates and proteins were washed twice with acetone, dried under a stream of nitrogen, and reconstituted in 3 ml of 20 mM bis-Tris/HCl (pH 6.5) loading buffer. Aliquots were chromatographically desalted on a HiPrep 26/10 Desalting column (GE Healthcare Life Sciences, Uppsala, Sweden) and then separated on an anion exchanger HiPrep 16/10 DEAE FF column (GE Healthcare Life Sciences) equilibrated with 20 mM bis-Tris/HCl (pH 6.5). Carbohydrate positive flow-through fractions were pooled together, lyophilized, resuspended in 15% acetonitrile in 150 mM ammonium bicarbonate, and applied to gel filtration on a HighPrep 16/60 Sephacryl S-300 HR column (GE Healthcare). All chromatographic separation steps were performed at room temperature on the high-performance liquid chromatography ÄKTA-Purifier 10 system (GE Healthcare Life Sciences).

### Monosugar composition analysis

Sugars were converted to alditol acetate derivatives according to the procedure described previously [48]. Monosugar alditol derivatives were identified and quantified by GLC-FID on a Shimadzu GC-2010 system (Shimadzu Co., Kyoto, Japan) using a (50% cyanopropylphenyl) methylpolysiloxane column (#007–225; 30 m × 0.25 mm with 0.25 μm film thickness,) (Quadrex Co., Woodbridge, Connecticut).

### NMR spectroscopy

The samples were dissolved in 100 μl water and precipitated by addition of 900 μl EtOH. After centrifugation, the precipitate was dried, dissolved in D_2_O (99.9% D, Sigma-Aldrich), and lyophilized. The sample was then dissolved in 280 μl D_2_O (99.96% D, Cambridge Isotope Laboratories) containing 0.5 μl acetone and placed into a 5-mm NMR tube with magnetic susceptibility plugs, matched to D_2_O (Shigemi). NMR experiments were recorded at 65°C on an Agilent Inova-600 spectrometer equipped with a 5-mm cryoprobe. The 1-D proton experiment was acquired in 8 transients with water presaturation. The 2-D COSY experiment was collected with gradient enhancement in 400 increments of 8 transients each. The 2-D TOCSY and NOESY experiments were acquired with water presaturation in 128 increments of 16 transients each. Spinlock time in TOCSY was 80 ms, and mixing time in NOESY was 200 ms. The gradient-enhanced ^1^H-^13^C HSQC experiment with adiabatic 180° carbon pulses and multiplicity editing was acquired in 128 increments of 64 transients each, with a spectral width of 18091 Hz in the carbon dimension. The gradient-enhanced ^1^H-^13^C HMBC experiment with adiabatic 180° carbon pulses was acquired in 128 increments of 128 transients each, with a spectral width of 18,091 Hz in the carbon dimension. Chemical shifts were measured relative to DSS at 0 ppm in both proton and carbon scales by setting the chemical shift of internal acetone to 2.218 ppm (proton) and 33.0 ppm (carbon). Chemical shifts assignments reported in S3 and S4 Tables were performed based on literature values reported elsewhere [49].

### In vitro biofilm and planktonic antifungal susceptibility assay

In vitro biofilm drug susceptibility to the antifungal fluconazole (at a concentration of 1,000 μg/ml) was assessed using a tetrazolium salt XTT reduction assay [16]. The percent reduction in biofilm growth compared to untreated controls is reported. Assays were performed in triplicate, and the significance of differences were assessed by one-way analysis of variance (ANOVA) with the posthoc Bonferroni and Holm methods [50] The CLSI M27 A3 broth microdilution susceptibility method was determine fluconazole activity against planktonic *Candida* strains. A visual turbidity endpoint was 24 h of grown was utilized.

### In vivo *Candida* venous catheter biofilm model

An external jugular vein rat catheter infection model was utilized for in vivo biofilm assessment [28, 37, 38]. Quantitative cultures of *C*. *albicans* after 24 h of in vivo growth was utilized to measure viable biofilm cell burden. For drug treatment experiments, fluconazole at a concentration of 250 μg/ml was instilled and dwelled in the catheter over a 24-h period. The post treatment viable burden of *Candida* biofilm on the catheter surface was compared to untreated control growth. Three replicates were performed for treatment and control conditions.

### Sequestration of ^3^H fluconazole in biofilms

Radiolabeled fluconazole was used to measure drug concentration in intact biofilms, matrix, and inside biofilm cells using a 6-well biofilm plate assay [37, 51]. After 48 hrs of biofilm growth, plates were washed and then incubated with 8.48 x 10^5^ cpm of ^3^H fluconazole (Moravek Biochemicals; 50 μM, 0.001 mCi/mL in ethanol). Unlabeled fluconazole (20 μM) in RPMI–MOPS was added for an additional 15-min incubation period and then washed to remove unbound fluconazole. Biofilm were collected with a spatula. Matrix and cells were isolated as described above. Intact biofilm, matrix, cell samples were added to a Tri-Carb 2100TR liquid scintillation analyzer after adding ScintiSafe 30% LSC mixture to each sample fraction. Three biologic and technical replicates performed. Values were compared to the reference strain using pairwise comparisons with ANOVA with the Holm-Sidak method.

### Sequestration of ^3^H fluconazole by EVs

A 100-μl sample of purified biofilm EVs (equivalent of 1000-ml biofilm culture) was used to assess fluconazole sequestration. Vesicles were mixed with an equivalent volume of the radiolabeled drug and incubated for 1 h at 37°C. The sample was centrifuged for 10 min at 14,000 *× g* followed by collection of supernatant and washed three times with 1 ml of PBS. The collected vesicle pellet was resuspended in 200 μl of PBS and added to a Tri-Carb 2100TR liquid scintillation analyzer after adding ScintiSafe 30% LSC mixture. Three replicates were used.

### EV addback assay

Biofilms were formed in the wells of 96-well microtiter plates, as described above. After a 5-h biofilm formation period, the biofilms were washed with PBS twice, and purified EVs at concentrations of 21804 ± 1711 EVs/ml were added. For treatment studies, after an additional hour of incubation, biofilm cultures were treated with fluconazole (1,000 μg/ml), followed by the drug treatment protocol described above. For biofilm matrix studies, the samples were incubated for an additional 24 hrs prior to either SEM imaging or matrix isolation for quantitative carbohydrate analysis.

## Supporting information

S1 Fig Sequestration of 3H-labelled fluconazole by extracellular vesicles isolated from *C. albicans* biofilm cultures. Purified extracellular vesicles were exposed to the radiolabeled drug (*FLC), washed, and both the supernatant and the vesicles were harvested. Scintillation counting was performed in triplicate on three biological samples to determine the fluconazole content in both fractions. Underlying data can be found in S1 Data.
(TIF)

S1 Table Biofilm and planktonic vesicle proteome comparison. (DOCX)

S2 Table Biofilm vesicle compared to biofilm matrix proteome profile. (DOCX)

S3 Table 1D 1H NMR chemical shift assignment of the major spin systems found in *C. albicans* extracellular matrix and extracellular vesicles. (DOCX)

S4 Table Chemical shift assignment of the major spin systems found in *C. albicans* extracellular vesicles. (DOCX)

S5 Table Primer sequences used for strain construction. (DOCX)

S6 Table Genotypes for strains used in this study. (DOCX)

S1 Data Data underlying Figs 1D–1F, 3B–3F, 4B, 4E and 4F and S1 Fig. (XLSX)

## Abbreviations

CSFE: carboxyfluorescein succinimidyl ester
ESCRT: endosomal sorting complexes required for transport
EV: extracellular vesicle
KEGG: Kyoto Encyclopedia of Genes and Genomes
KOID: KEGG Ontology ID
MGCx: mannan–glucan complex
MOPS: 4-morpholinepropanesulfonic acid
MTL: mating type locus
PBS: phosphate-buffered saline
Phr1: putative glycanosyltransferase
SEM: scanning electron micrograph
Sun41: putative endo-beta-D-glucosidase
WT: wild-type
