# Supplementary figures and images for: Candida albicans biofilm–induced vesicles confer drug resistance through matrix biogenesis
Source: PLoS Biol. 2018 Oct 8;16(10):e2006872. doi: 10.1371/journal.pbio.2006872 (PMC6209495; doi:10.1371/journal.pbio.2006872)

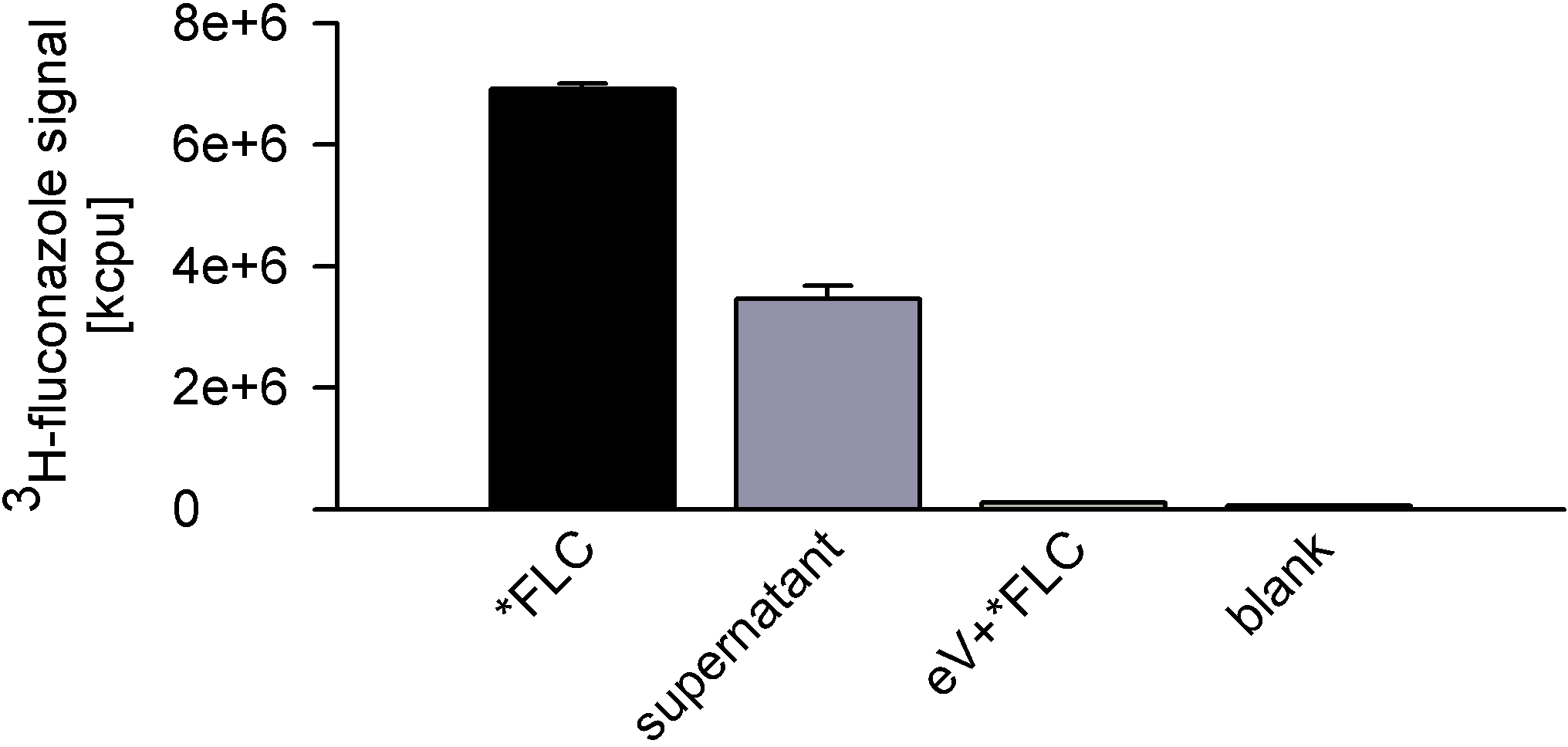

Supplement: S1 Fig — Purified extracellular vesicles were exposed to the radiolabeled drug (*FLC), washed, and both the supernatant and the vesicles were harvested. Scintillation counting was performed in triplicate on three biological samples to determine the fluconazole content in both fractions. Underlying data can be found in S1 Data. (TIF) [file pbio.2006872.s001.tif]
