# Supplementary material for: Candida albicans biofilm–induced vesicles confer drug resistance through matrix biogenesis
Source: PLoS Biol. 2018 Oct 8;16(10):e2006872. doi: 10.1371/journal.pbio.2006872 (PMC6209495; doi:10.1371/journal.pbio.2006872)
Supplement: S1 Table — (DOCX) [file pbio.2006872.s002.docx]

**S1 Table. Biofilm and planktonic vesicle proteome comparison.**

| **Proteomics Profiles** | | | | | | |
| --- | --- | --- | --- | --- | --- | --- |
| **Protein Characteristics** | | | | **Quantitative Value**  **(Normalized Total Spectra)^1^** | | **Biofilm/Planktonic**  **Ratio** |
| **Protein Name** | **Gene Name** | **ORF Number** | **UniProt ID** | **Biofilm EVs** | **Planktonic EVs** |  |
| Candidapepsin-6 | SAP6 | CaO19.12988 | Q5AC08 | 164.74 | 0.00 | biofilm unique |
| Cell surface Cu-only superoxide dismutase 5 | SOD5 | CaO19.2060 | Q5AD07 | 52.36 | 0.00 | biofilm unique |
| Candidapepsin-4 | SAP4 | CaO19.13139 | Q5A8N2 | 34.28 | 0.00 | biofilm unique |
| Uncharacterized protein |  | CAALFM_C602100WA | A0A1D8PPT3 | 27.96 | 0.00 | biofilm unique |
| Lipase 4 | LIP4 | CaO19.2133 | Q9P8W1 | 26.53 | 0.00 | biofilm unique |
| Rax2p | RAX2 | CAALFM_C112510WA | A0A1D8PFE5 | 22.03 | 0.00 | biofilm unique |
| Uncharacterized protein |  | CAALFM_C209800CA | Q59YF6 | 18.96 | 0.00 | biofilm unique |
| Profilin | PFY1 | CAALFM_C108030WA | Q5A786 | 17.01 | 0.00 | biofilm unique |
| Glucan 1,3-beta-glucosidase 2 | EXG2 | CaO19.10469 | Q5AIA1 | 16.26 | 0.00 | biofilm unique |
| Uncharacterized protein |  | CAALFM_CR06030CA | A0A1D8PT50 | 16.21 | 0.00 | biofilm unique |
| Predicted GPI-anchored protein 17 | PGA17 | CaO19.8512 | Q5AHA4 | 16.18 | 0.00 | biofilm unique |
| Surface antigen protein 2 | CSA2 | CaO19.10629 | Q5A0X8 | 15.69 | 0.00 | biofilm unique |
| Uncharacterized protein |  | CAALFM_C210150WA | A0A1D8PIN7 | 15.37 | 0.00 | biofilm unique |
| Kexin | KEX2 | CAALFM_C108990CA | A0A1D8PEG3 | 13.87 | 0.00 | biofilm unique |
| Ferroxidase | FET34 | CAALFM_C600440CA | A0A1D8PPC9 | 12.48 | 0.00 | biofilm unique |
| Beta-mannosyltransferase 6 | BMT6 | CaO19.13045 | Q5ABU8 | 12.23 | 0.00 | biofilm unique |
| Pbr1p | PBR1 | CAALFM_C106370CA | Q5AAN7 | 10.35 | 0.00 | biofilm unique |
| Glucose-6-phosphate 1-dehydrogenase | ZWF1 | CAALFM_C108980CA | A0A1D8PEG2 | 9.70 | 0.00 | biofilm unique |
| Mannan endo-1,6-alpha-mannosidase | DCW1 | CaO19.1989 | Q5AD78 | 9.66 | 0.00 | biofilm unique |
| Rdi1p | RDI1 | CAALFM_C305000WA | Q5AND4 | 9.30 | 0.00 | biofilm unique |
| Putative NADPH-dependent methylglyoxal reductase | GRP2 | CaO19.11785 | P83775 | 9.29 | 0.00 | biofilm unique |
| Glucose-6-phosphate 1-epimerase |  | CAALFM_C501230CA | Q5A1Q0 | 9.26 | 0.00 | biofilm unique |
| Uncharacterized protein |  | CAALFM_CR09240CA | A0A1D8PTY0 | 9.25 | 0.00 | biofilm unique |
| 17-beta-hydroxysteroid dehydrogenase-like protein |  | CAALFM_C114060WA | A0A1D8PFV8 | 8.75 | 0.00 | biofilm unique |
| Induced during hyphae development protein 1 | IHD1 | CaO19.13183 | Q5A8I8 | 7.72 | 0.00 | biofilm unique |
| Lysophospholipase | PLB5 | CAALFM_C108230CA | A0A1D8PEB1 | 6.83 | 0.00 | biofilm unique |
| Fet31p | FET31 | CAALFM_C600480CA | A0A1D8PPE2 | 6.29 | 0.00 | biofilm unique |
| Uncharacterized protein |  | CAALFM_C104010CA | A0A1D8PD74 | 5.97 | 0.00 | biofilm unique |
| Bleomycin hydrolase | LAP3 | CAALFM_CR04480CA | Q5A6L5 | 5.79 | 0.00 | biofilm unique |
| Glucan 1,4-alpha-glucosidase | SGA1 | CAALFM_C301320CA | Q5AJ73 | 5.69 | 0.00 | biofilm unique |
| Ifd6p | IFD6 | CAALFM_C104140WA | A0A1D8PD78 | 4.50 | 0.00 | biofilm unique |
| Metalloendopeptidase | STE23 | CAALFM_C602860WA | A0A1D8PQ06 | 4.25 | 0.00 | biofilm unique |
| Phosphatidylglycerol/phosphatidylinositol transfer protein (PG/PI-TP) | NPC2 | CaO19.10736 | Q5A8A2 | 4.17 | 0.00 | biofilm unique |
| Survival factor 1 | SVF1 | CaO19.13489 | Q5ABA2 | 4.02 | 0.00 | biofilm unique |
| Beta-mannosyltransferase 1 | BMT1 | CaO19.14074 | Q5ADQ9 | 4.01 | 0.00 | biofilm unique |
| Orotate phosphoribosyltransferase | URA5 | CAALFM_CR01650WA | A0A1D8PS11 | 3.97 | 0.00 | biofilm unique |
| Low-affinity Zn(2+) transporter | ZRT2 | CAALFM_C202590WA | A0A1D8PGN5 | 3.93 | 0.00 | biofilm unique |
| Emp46p | EMP46 | CAALFM_CR07180WA | A0A1D8PTF1 | 3.79 | 0.00 | biofilm unique |
| Pheromone-processing carboxypeptidase | KEX1 | CaJ7.0112 | Q5AFP8 | 3.75 | 0.00 | biofilm unique |
| Palmitoyltransferase | YKT6 | CAALFM_C102860CA | Q5AI79 | 3.74 | 0.00 | biofilm unique |
| Coproporphyrinogen oxidase | HEM13 | CAALFM_C304060CA | Q59MR4 | 3.59 | 0.00 | biofilm unique |
| Mid1p | MID1 | CAALFM_C503990WA | A0A1D8PNU4 | 3.47 | 0.00 | biofilm unique |
| Spermidine synthase | SPE3 | CAALFM_C206960WA | Q59Z50 | 3.40 | 0.00 | biofilm unique |
| Importin-alpha export receptor |  | CAALFM_C107690CA | A0A1D8PE78 | 3.40 | 0.00 | biofilm unique |
| High-affinity glucose transporter 1 | HGT1 | orf19.4527 | A0A1D8PCL1 | 3.29 | 0.00 | biofilm unique |
| Axl2p | AXL2 | CAALFM_C404170CA | A0A1D8PM01 | 3.26 | 0.00 | biofilm unique |
| Dynein light chain |  | CAALFM_C201440CA | A0A1D8PGE0 | 3.07 | 0.00 | biofilm unique |
| Cfl2p | CFL2 | CAALFM_C405780CA | A0A1D8PME3 | 3.00 | 0.00 | biofilm unique |
| Uncharacterized protein |  | CAALFM_C403480CA | A0A1D8PLU2 | 2.99 | 0.00 | biofilm unique |
| Secreted protein | PRY1 | CaO19.10303 | Q59PV6 | 2.82 | 0.00 | biofilm unique |
| Arginase | CAR1 | CAALFM_C504490CA | A0A1D8PP00 | 2.74 | 0.00 | biofilm unique |
| Aha1p | AHA1 | CAALFM_CR10270CA | A0A1D8PU72 | 2.71 | 0.00 | biofilm unique |
| Peptide hydrolase | APE3 | CAALFM_C208800CA | A0A1D8PI95 | 2.69 | 0.00 | biofilm unique |
| Argininosuccinate synthase | ARG1 | CAALFM_CR00620CA | A0A1D8PRR5 | 2.61 | 0.00 | biofilm unique |
| FK506-binding protein 1 | RBP1 | CaO19.11186 | P28870 | 2.52 | 0.00 | biofilm unique |
| Cip1p | CIP1 | CAALFM_C601070CA | A0A1D8PPI6 | 2.46 | 0.00 | biofilm unique |
| Beta-mannosyltransferase 8 | BMT8 | CaO19.8479 | Q5AHD6 | 2.31 | 0.00 | biofilm unique |
| Rot1p | ROT1 | CaO19.13450 | Q5ABP8 | 2.31 | 0.00 | biofilm unique |
| Cell wall protein PGA31 | PGA31 | CaO19.12761 | Q5A5U9 | 2.30 | 0.00 | biofilm unique |
| Trifunctional aldehyde reductase/xylose reductase/glucose 1-dehydrogenase (NADP(+)) | GRE3 | CAALFM_C502930CA | A0A1D8PNK3 | 2.19 | 0.00 | biofilm unique |
| Uncharacterized protein |  | CAALFM_C109670CA | Q5APD5 | 2.17 | 0.00 | biofilm unique |
| Putative alpha-1,3-mannosyltransferase | MNN12 | CaO19.12366 | Q5APQ8 | 2.12 | 0.00 | biofilm unique |
| Ldg3p | LDG3 | CAALFM_C702270WA | A0A1D8PR06 | 2.10 | 0.00 | biofilm unique |
| Putative phosphoric monoester hydrolase |  | CAALFM_C207140WA | A0A1D8PHU6 | 2.06 | 0.00 | biofilm unique |
| Hsp90 cochaperone | SBA1 | CAALFM_C603750CA | A0A1D8PQ94 | 2.06 | 0.00 | biofilm unique |
| Sap99p | SAP99 | CAALFM_C203670WA | A0A1D8PGZ9 | 2.05 | 0.00 | biofilm unique |
| L-methionine (R)-S-oxide reductase |  | CAALFM_C602800WA | A0A1D8PPZ9 | 2.05 | 0.00 | biofilm unique |
| mRNA splicing protein |  | CAALFM_C203880CA | A0A1D8PH20 | 2.04 | 0.00 | biofilm unique |
| Uncharacterized protein |  | CAALFM_C503930CA | A0A1D8PNT7 | 1.95 | 0.00 | biofilm unique |
| Cell surface superoxide dismutase | SOD4 | CaO19.2062 | Q5AD05 | 1.86 | 0.00 | biofilm unique |
| Uncharacterized protein |  | CAALFM_C703370CA | A0A1D8PRB4 | 1.83 | 0.00 | biofilm unique |
| Uncharacterized protein |  | CAALFM_C402340WA | Q5AF37 | 1.82 | 0.00 | biofilm unique |
| Uncharacterized protein |  | CAALFM_C210120WA | A0A1D8PIN9 | 1.79 | 0.00 | biofilm unique |
| Glutathione-disulfide reductase |  | CAALFM_C501560CA | Q59NQ2 | 1.79 | 0.00 | biofilm unique |
| Proteinase B |  | CAALFM_C703860WA | A0A1D8PRH0 | 1.78 | 0.00 | biofilm unique |
| 6-phosphogluconolactonase | SOL3 | CAALFM_CR06700CA | Q59PZ6 | 1.77 | 0.00 | biofilm unique |
| Rho family GTPase | RAC1 | CAALFM_C106730WA | A0A1D8PDV5 | 1.64 | 0.00 | biofilm unique |
| Uncharacterized protein |  | CAALFM_C101910WA | A0A1D8PCL4 | 1.59 | 0.00 | biofilm unique |
| Zrt1p | ZRT1 | CAALFM_C406970CA | A0A1D8PMR6 | 1.59 | 0.00 | biofilm unique |
| Inosine triphosphate pyrophosphatase (ITPase) | HAM1 | CaO19.1108 | Q59N80 | 1.59 | 0.00 | biofilm unique |
| Uncharacterized protein |  | CAALFM_CR06500CA | A0A1D8PTA6 | 1.56 | 0.00 | biofilm unique |
| Lip1p | LIP1 | CAALFM_C109580CA | A0A1D8PER6 | 1.53 | 0.00 | biofilm unique |
| Proteasome subunit beta |  | CAALFM_C502230WA | A0A1D8PNC8 | 1.51 | 0.00 | biofilm unique |
| High osmolarity signaling protein | SHO1 | CaO19.12235 | Q5AQ36 | 1.48 | 0.00 | biofilm unique |
| Doa1p | DOA1 | CAALFM_C109660WA | A0A1D8PEM3 | 1.45 | 0.00 | biofilm unique |
| Probable pathogenesis-related protein |  | CaO19.13580 | Q5AB49 | 1.44 | 0.00 | biofilm unique |
| Branched-chain-amino-acid aminotransferase | BAT22 | CAALFM_C305590CA | A0A1D8PKB9 | 1.44 | 0.00 | biofilm unique |
| Peptide hydrolase |  | CAALFM_C105670WA | Q5AA00 | 1.39 | 0.00 | biofilm unique |
| Translation initiation factor eIF1 | SUI1 | CAALFM_C504090CA | Q59LQ6 | 1.34 | 0.00 | biofilm unique |
| Cell-surface associated glycoprotein | DFI1 | CaO19.7084 | Q5AFI4 | 1.29 | 0.00 | biofilm unique |
| Uncharacterized protein |  | CAALFM_C700340CA | A0A1D8PQJ7 | 1.27 | 0.00 | biofilm unique |
| Proteasome endopeptidase complex | SCL1 | CAALFM_C300770CA | A0A1D8PJ20 | 1.20 | 0.00 | biofilm unique |
| Arf family GTPase | ARL1 | CAALFM_C401890CA | Q5AMP3 | 1.18 | 0.00 | biofilm unique |
| Phosphatidylinositol transfer protein | SFH5 | CaO19.12362 | Q5AP66 | 1.18 | 0.00 | biofilm unique |
| S-adenosylmethionine permease | GAP4 | CAALFM_C107120WA | Q59WB3 | 1.17 | 0.00 | biofilm unique |
| Uncharacterized protein |  | CAALFM_C602330WA | A0A1D8PPV6 | 1.11 | 0.00 | biofilm unique |
| Peptidyl-prolyl cis-trans isomerase (PPIase) |  | CAALFM_C305550CA | A0A1D8PKA6 | 1.06 | 0.00 | biofilm unique |
| Pyridoxine 4-dehydrogenase |  | CAALFM_CR09100CA | A0A1D8PTW9 | 1.06 | 0.00 | biofilm unique |
| Cytochrome c | CYC1 | CaO19.1770 | P53698 | 1.04 | 0.00 | biofilm unique |
| Uncharacterized protein |  | CAALFM_C600980CA | A0A1D8PPI4 | 1.02 | 0.00 | biofilm unique |
| Candidapepsin-7 | SAP7 | CaO19.756 | Q59VH7 | 0.99 | 0.00 | biofilm unique |
| Uncharacterized protein |  | CAALFM_C208770CA | Q59Y55 | 0.98 | 0.00 | biofilm unique |
| Nascent polypeptide-associated complex subunit alpha | EGD2 | CaO19.13280 | Q5ANP2 | 0.98 | 0.00 | biofilm unique |
| Putative amidotransferase |  | CAALFM_C205590CA | Q59T45 | 0.98 | 0.00 | biofilm unique |
| Mtr10p | MTR10 | CAALFM_C503760CA | A0A1D8PNS3 | 0.96 | 0.00 | biofilm unique |
| Kre5p | KRE5 | CAALFM_C302960CA | A0A1D8PJN6 | 0.89 | 0.00 | biofilm unique |
| Tyrosine protein phosphatase | LTP1 | CAALFM_C108260CA | A0A1D8PE91 | 0.87 | 0.00 | biofilm unique |
| Ribonuclease T2-like 1-B | RNY1-B | CaO19.11391 | Q5AKB1 | 0.82 | 0.00 | biofilm unique |
| Deoxyuridine 5'-triphosphate nucleotidohydrolase (dUTPase) | DUT1 | CaO19.10832 | P0CY19 | 0.81 | 0.00 | biofilm unique |
| Uncharacterized protein |  | CAALFM_C207630CA | Q59U89 | 0.80 | 0.00 | biofilm unique |
| Uncharacterized protein |  | CAALFM_CR06510WA | A0A1D8PT83 | 0.75 | 0.00 | biofilm unique |
| Leukotriene A-4 hydrolase homolog (LTA-4 hydrolase) | LKH1 | CaO19.8607 | Q59NB8 | 0.75 | 0.00 | biofilm unique |
| Proteasome endopeptidase complex | PRE10 | CAALFM_C701470CA | A0A1D8PQW1 | 0.74 | 0.00 | biofilm unique |
| Uncharacterized protein |  | CAALFM_C401660WA | A0A1D8PLF7 | 0.73 | 0.00 | biofilm unique |
| Cdr11p | CDR11 | CAALFM_C304070CA | A0A1D8PK15 | 0.73 | 0.00 | biofilm unique |
| Uncharacterized protein |  | CAALFM_C104150CA | Q59VP4 | 0.72 | 0.00 | biofilm unique |
| Aromatic-amino-acid:2-oxoglutarate transaminase | ARO9 | CAALFM_C405560CA | A0A1D8PMC5 | 0.70 | 0.00 | biofilm unique |
| Uncharacterized protein |  | CAALFM_C202720WA | A0A1D8PGN9 | 0.63 | 0.00 | biofilm unique |
| Uncharacterized protein |  | CAALFM_C401460CA | A0A1D8PLE6 | 0.63 | 0.00 | biofilm unique |
| Rab geranylgeranyltransferase | BET2 | CAALFM_CR09890CA | A0A1D8PU24 | 0.60 | 0.00 | biofilm unique |
| Uncharacterized protein |  | CAALFM_C403500CA | A0A1D8PLV4 | 0.59 | 0.00 | biofilm unique |
| Riboflavin synthase | RIB5 | CAALFM_C505300WA | A0A1D8PP67 | 0.58 | 0.00 | biofilm unique |
| cAMP-dependent protein kinase regulatory subunit (PKA regulatory subunit) | BCY1 | CaO19.9565 | Q9HEW1 | 0.58 | 0.00 | biofilm unique |
| Uncharacterized protein |  | CAALFM_C100270WA | A0A1D8PC75 | 0.50 | 0.00 | biofilm unique |
| Uncharacterized protein |  | CAALFM_C103240WA | A0A1D8PD02 | 0.50 | 0.00 | biofilm unique |
| Uncharacterized protein |  | CAALFM_C204850CA | A0A1D8PH89 | 0.50 | 0.00 | biofilm unique |
| Co-chaperone | SGT1 | CAALFM_C209270CA | A0A1D8PIG5 | 0.50 | 0.00 | biofilm unique |
| E2 ubiquitin-conjugating protein |  | CAALFM_C604280WA | A0A1D8PQD4 | 0.50 | 0.00 | biofilm unique |
| Ran GTPase-binding protein | NTF2 | CAALFM_C110100CA | A0A1D8PES3 | 0.49 | 0.00 | biofilm unique |
| Vacuolar protein sorting-associated protein 27 | VPS27 | CaO19.13452 | Q5ABD9 | 0.48 | 0.00 | biofilm unique |
| Hgt2p | HGT2 | CAALFM_C102110CA | Q59VZ0 | 0.42 | 0.00 | biofilm unique |
| Dip5p | DIP5 | CAALFM_C102530CA | A0A1D8PCR4 | 0.40 | 0.00 | biofilm unique |
| Adenosine kinase | ADO1 | CAALFM_C603080CA | A0A1D8PQ26 | 0.39 | 0.00 | biofilm unique |
| ATP synthase subunit beta | ATP2 | CAALFM_C400270WA | A0A1D8PKZ9 | 0.38 | 0.00 | biofilm unique |
| Epsin |  | CAALFM_C201390WA | A0A1D8PGB7 | 0.35 | 0.00 | biofilm unique |
| Tubulin-binding prefolding complex subunit |  | CAALFM_C111110CA | A0A1D8PF30 | 0.34 | 0.00 | biofilm unique |
| Ent3p | ENT3 | CAALFM_C202340CA | A0A1D8PGL6 | 0.34 | 0.00 | biofilm unique |
| Hsp12p (Lipid-binding protein) | HSP12 | CAALFM_C502080CA | A0A1D8PNC7 | 0.34 | 0.00 | biofilm unique |
| Uncharacterized protein |  | CAALFM_CR06530WA | A0A1D8PT84 | 0.34 | 0.00 | biofilm unique |
| Aminopeptidase 2 | APE2 | CaO19.12664 | Q59KZ1 | 0.33 | 0.00 | biofilm unique |
| Sgt2p | SGT2 | CAALFM_C202830CA | Q5A0I8 | 0.33 | 0.00 | biofilm unique |
| Ribonuclease H |  | CAALFM_C603260WA | A0A1D8PQ43 | 0.33 | 0.00 | biofilm unique |
| F-actin-capping protein subunit alpha | CAP01 | CaO19.10745 | Q5A893 | 0.33 | 0.00 | biofilm unique |
| Methylthioribose-1-phosphate isomerase | MRI1 | CaO19.2008 | Q5AD59 | 0.31 | 0.00 | biofilm unique |
| RNA export factor | GLE2 | CAALFM_C604360CA | A0A1D8PQE5 | 0.31 | 0.00 | biofilm unique |
| Uncharacterized protein |  | CAALFM_C203020CA | Q5A0L2 | 0.29 | 0.00 | biofilm unique |
| Uncharacterized protein |  | CAALFM_C209980WA | A0A1D8PIK6 | 0.27 | 0.00 | biofilm unique |
| Uncharacterized protein |  | CAALFM_C200770WA | Q5AD20 | 0.25 | 0.00 | biofilm unique |
| Carboxypeptidase | PRC2 | CAALFM_C501450WA | A0A1D8PN69 | 0.25 | 0.00 | biofilm unique |
| mRNA-binding protein | PUF3 | CAALFM_C405370WA | A0A1D8PMA5 | 0.25 | 0.00 | biofilm unique |
| Uncharacterized protein |  | CAALFM_C702260WA | Q5AH51 | 0.25 | 0.00 | biofilm unique |
| Proteasome core particle subunit beta 4 | PRE1 | CAALFM_C505310WA | Q5AJZ5 | 0.25 | 0.00 | biofilm unique |
| Uncharacterized protein |  | CAALFM_CR08990CA | A0A1D8PTU3 | 0.25 | 0.00 | biofilm unique |
| Elf1p | ELF1 | CAALFM_CR09370WA | A0A1D8PTY4 | 0.25 | 0.00 | biofilm unique |
| Cystathionine gamma-lyase | CYS3 | CAALFM_CR08340WA | Q5A362 | 0.23 | 0.00 | biofilm unique |
| Class E vacuolar protein-sorting machinery protein | HSE1 | CaO19.10743 | Q5A895 | 0.23 | 0.00 | biofilm unique |
| Autophagy-related protein 27 | ATG27 | CaO19.12782 | Q5A5S7 | 0.23 | 0.00 | biofilm unique |
| Uncharacterized protein |  | CAALFM_C406130WA | A0A1D8PMG9 | 0.20 | 0.00 | biofilm unique |
| Mdg1p | MDG1 | CAALFM_C114290CA | A0A1D8PFV7 | 0.18 | 0.00 | biofilm unique |
| Uncharacterized protein |  | CAALFM_C209070CA | A0A1D8PIC6 | 0.18 | 0.00 | biofilm unique |
| Sbp1p | SBP1 | CAALFM_C304090WA | Q5ANP6 | 0.18 | 0.00 | biofilm unique |
| Phosphoribomutase |  | CAALFM_C307490WA | Q5ADV2 | 0.18 | 0.00 | biofilm unique |
| Calmodulin | CMD1 | CAALFM_C406030WA | A0A1D8PMF8 | 0.18 | 0.00 | biofilm unique |
| Bifunctional choline kinase/ethanolamine kinase |  | CAALFM_C305300CA | A0A1D8PK80 | 0.17 | 0.00 | biofilm unique |
| Cwh41p | CWH41 | CAALFM_C406100WA | A0A1D8PMH9 | 0.17 | 0.00 | biofilm unique |
| Exportin-T | LOS1 | CaJ7.0486 | Q5A0E2 | 0.17 | 0.00 | biofilm unique |
| Peptidylprolyl isomerase |  | CAALFM_C113260WA | A0A1D8PFM9 | 0.17 | 0.00 | biofilm unique |
| Uncharacterized protein |  | CAALFM_C201340WA | A0A1D8PGD2 | 0.17 | 0.00 | biofilm unique |
| Uncharacterized protein |  | CAALFM_C400710WA | A0A1D8PL57 | 0.17 | 0.00 | biofilm unique |
| Dap2p | DAP2 | CAALFM_C502970WA | A0A1D8PNK0 | 0.17 | 0.00 | biofilm unique |
| Ubiquitin-related modifier 1 | URM1 | CaO19.1016 | Q59WK2 | 0.17 | 0.00 | biofilm unique |
| Protein transport protein SSO2 | SSO2 | CaO19.1376 | Q59YF0 | 0.17 | 0.00 | biofilm unique |
| C-8 sterol isomerase | ERG2 | CAALFM_C100800CA | A0A1D8PCB9 | 0.17 | 0.00 | biofilm unique |
| Aldehyde dehydrogenase (NAD(P)(+)) | ALD5 | CAALFM_C202970CA | A0A1D8PGT5 | 0.17 | 0.00 | biofilm unique |
| Uncharacterized protein |  | CAALFM_C204330CA | Q59TB0 | 0.17 | 0.00 | biofilm unique |
| Uncharacterized protein |  | CAALFM_C503490CA | A0A1D8PNP4 | 0.17 | 0.00 | biofilm unique |
| Lactoylglutathione lyase | GLO1 | CAALFM_C100500CA | Q5ABB2 | 0.16 | 0.00 | biofilm unique |
| Rho family GTPase | CRL1 | CAALFM_C101420CA | Q5A904 | 0.16 | 0.00 | biofilm unique |
| Eukaryotic translation initiation factor 2A |  | CAALFM_C102430CA | A0A1D8PCQ3 | 0.16 | 0.00 | biofilm unique |
| Proteasome regulatory particle lid subunit |  | CAALFM_C104230WA | A0A1D8PDB0 | 0.16 | 0.00 | biofilm unique |
| Peptide-methionine-S-sulfoxide reductase | MXR1 | CAALFM_C200960CA | Q5AD39 | 0.16 | 0.00 | biofilm unique |
| AMP deaminase |  | CAALFM_C203360WA | Q5AHA6 | 0.16 | 0.00 | biofilm unique |
| Uncharacterized protein |  | CAALFM_C205550WA | A0A1D8PHE5 | 0.16 | 0.00 | biofilm unique |
| D-arabinose 1-dehydrogenase (NAD(P)(+)) | ARA1 | CAALFM_C208130WA | A0A1D8PI24 | 0.16 | 0.00 | biofilm unique |
| Kinase | IPK2 | CAALFM_C209730CA | Q59YE9 | 0.16 | 0.00 | biofilm unique |
| Uncharacterized protein |  | CAALFM_C300850CA | A0A1D8PJ10 | 0.16 | 0.00 | biofilm unique |
| Uncharacterized protein |  | CAALFM_C304920CA | Q5ANE2 | 0.16 | 0.00 | biofilm unique |
| Putative GTPase | RHB1 | CAALFM_C305180WA | Q5ANA9 | 0.16 | 0.00 | biofilm unique |
| Rct1p | RCT1 | CAALFM_C305710WA | A0A1D8PKD1 | 0.16 | 0.00 | biofilm unique |
| Monothiol glutaredoxin | GRX3 | CAALFM_C402710CA | Q5AF81 | 0.16 | 0.00 | biofilm unique |
| Oye32p | OYE32 | CAALFM_C406780CA | A0A1D8PMP0 | 0.16 | 0.00 | biofilm unique |
| Tfs1p | TFS1 | CAALFM_C500930CA | Q5A1M1 | 0.16 | 0.00 | biofilm unique |
| mRNA splicing protein |  | CAALFM_C503110CA | A0A1D8PNM3 | 0.16 | 0.00 | biofilm unique |
| Ornithine carbamoyltransferase | ARG3 | CAALFM_C603230WA | Q5ABU0 | 0.16 | 0.00 | biofilm unique |
| DNA-directed RNA polymerases I, II, and III subunit | RPB8 | CAALFM_CR04780WA | Q59MZ8 | 0.16 | 0.00 | biofilm unique |
| S-methyl-5'-thioadenosine phosphorylase | MEU1 | CaO19.14200 | Q59ST1 | 0.16 | 0.00 | biofilm unique |
| mRNA export factor | MEX67 | CaO19.488 | P84149 | 0.16 | 0.00 | biofilm unique |
| Vac7p | VAC7 | CAALFM_C404370CA | Q59PA6 | 0.15 | 0.00 | biofilm unique |
| Uncharacterized protein |  | CAALFM_C108770WA | A0A1D8PEE6 | 0.10 | 0.00 | biofilm unique |
| Spa2p | SPA2 | CAALFM_C112620WA | A0A1D8PFH4 | 0.10 | 0.00 | biofilm unique |
| Uncharacterized protein |  | CAALFM_C207650CA | A0A1D8PHY7 | 0.10 | 0.00 | biofilm unique |
| Uncharacterized protein |  | CAALFM_C207810WA | Q5A2K5 | 0.10 | 0.00 | biofilm unique |
| Nmd5p | NMD5 | CAALFM_C400540CA | Q59SI4 | 0.10 | 0.00 | biofilm unique |
| Imp2'p |  | CAALFM_C406730CA | Q5A0W0 | 0.10 | 0.00 | biofilm unique |
| Uncharacterized protein |  | CAALFM_C700630CA | A0A1D8PQM7 | 0.10 | 0.00 | biofilm unique |
| Uncharacterized protein |  | CAALFM_C703830CA | A0A1D8PRF3 | 0.10 | 0.00 | biofilm unique |
| Uncharacterized protein |  | CAALFM_CR00230WA | A0A1D8PRM4 | 0.10 | 0.00 | biofilm unique |
| Uncharacterized protein |  | CAALFM_CR09580CA | A0A1D8PU09 | 0.10 | 0.00 | biofilm unique |
| Cullin-associated NEDD8-dissociated protein 1 | TIP120 | CaO19.14021 | Q5ADW3 | 0.10 | 0.00 | biofilm unique |
| Translation initiation factor eIF-2B subunit epsilon | GCD6 | CaO19.407 | P87163 | 0.10 | 0.00 | biofilm unique |
| Uncharacterized protein |  | CAALFM_C104600CA | A0A1D8PDC7 | 0.10 | 0.00 | biofilm unique |
| Uncharacterized protein |  | CAALFM_C105160CA | A0A1D8PDH0 | 0.10 | 0.00 | biofilm unique |
| Pfk26p | PFK26 | CAALFM_C108950WA | A0A1D8PEF9 | 0.10 | 0.00 | biofilm unique |
| Uncharacterized protein |  | CAALFM_C110890CA | A0A1D8PEZ3 | 0.10 | 0.00 | biofilm unique |
| Cta3p | CTA3 | CAALFM_C111540CA | A0A1D8PF53 | 0.10 | 0.00 | biofilm unique |
| E2 ubiquitin-conjugating protein |  | CAALFM_C112650CA | A0A1D8PFF9 | 0.10 | 0.00 | biofilm unique |
| Uncharacterized protein |  | CAALFM_C208100WA | A0A1D8PI22 | 0.10 | 0.00 | biofilm unique |
| Acyl-coenzyme A oxidase | PXP2 | CAALFM_C301930WA | Q5AJD6 | 0.10 | 0.00 | biofilm unique |
| Myosin 1 | MYO1 | CAALFM_C501650CA | A0A1D8PN92 | 0.10 | 0.00 | biofilm unique |
| DNA-directed RNA polymerase subunit beta | RPA135 | CAALFM_C700570WA | A0A1D8PQM4 | 0.10 | 0.00 | biofilm unique |
| Aldehyde dehydrogenase |  | CAALFM_C100410CA | A0A1D8PC76 | 0.08 | 0.00 | biofilm unique |
| Trm2p | TRM2 | CAALFM_C101380CA | A0A1D8PCG3 | 0.08 | 0.00 | biofilm unique |
| Carnitine O-acetyltransferase | CTN1 | CAALFM_C101740WA | Q59T80 | 0.08 | 0.00 | biofilm unique |
| Uncharacterized protein |  | CAALFM_C104360CA | A0A1D8PD97 | 0.08 | 0.00 | biofilm unique |
| Carboxypeptidase | PRC3 | CAALFM_C105770CA | A0A1D8PDM9 | 0.08 | 0.00 | biofilm unique |
| Isoleucine biosynthesis protein | MMD1 | CAALFM_C106900CA | A0A1D8PDX5 | 0.08 | 0.00 | biofilm unique |
| Nicotinate-nucleotide pyrophosphorylase |  | CAALFM_C107840WA | A0A1D8PE45 | 0.08 | 0.00 | biofilm unique |
| Gcr3p | GCR3 | CAALFM_C108420WA | A0A1D8PEB4 | 0.08 | 0.00 | biofilm unique |
| Rad23p | RAD23 | CAALFM_C201850WA | A0A1D8PGE3 | 0.08 | 0.00 | biofilm unique |
| Uncharacterized protein |  | CAALFM_C206770WA | A0A1D8PHR2 | 0.08 | 0.00 | biofilm unique |
| Prb1p | PRB1 | CAALFM_C206880CA | Q59Z57 | 0.08 | 0.00 | biofilm unique |
| Ato1p | ATO1 | CAALFM_C300920WA | A0A1D8PJ22 | 0.08 | 0.00 | biofilm unique |
| Putative flavin adenine dinucleotide transporter | FLC1 | CAALFM_C300980WA | A0A1D8PJ45 | 0.08 | 0.00 | biofilm unique |
| Skn1p | SKN1 | CAALFM_C305810CA | A0A1D8PKE1 | 0.08 | 0.00 | biofilm unique |
| Midasin | MDN1 | CAALFM_C400970CA | A0A1D8PL61 | 0.08 | 0.00 | biofilm unique |
| Glycerophosphodiester transporter | GIT2 | CAALFM_C500890CA | A0A1D8PN12 | 0.08 | 0.00 | biofilm unique |
| Uncharacterized protein |  | CAALFM_C505340WA | A0A1D8PP79 | 0.08 | 0.00 | biofilm unique |
| Long-chain fatty acid transporter | FAT1 | CAALFM_C600740WA | Q59NN4 | 0.08 | 0.00 | biofilm unique |
| Uncharacterized protein |  | CAALFM_C604410CA | A0A1D8PQE3 | 0.08 | 0.00 | biofilm unique |
| Long-chain fatty acid transporter | ACB1 | CAALFM_C700750WA | A0A1D8PQN3 | 0.08 | 0.00 | biofilm unique |
| Lmo1p | LMO1 | CAALFM_C703080WA | A0A1D8PR98 | 0.08 | 0.00 | biofilm unique |
| Mlc1p | MLC1 | CAALFM_CR03090CA | A0A1D8PSE1 | 0.08 | 0.00 | biofilm unique |
| Phospho-2-dehydro-3-deoxyheptonate aldolase | ARO4 | CaO19.11542 | P79023 | 0.08 | 0.00 | biofilm unique |
| Serine/threonine-protein phosphatase | SIT4 | CaO19.12667 | Q59KY8 | 0.08 | 0.00 | biofilm unique |
| Long chronological lifespan protein 2 | LCL2 | CaO19.13952 | Q59PT4 | 0.08 | 0.00 | biofilm unique |
| Histone acetyltransferase type B subunit 2 | HAT2 | CaO19.2146 | Q59RH5 | 0.08 | 0.00 | biofilm unique |
| D-arabinono-1,4-lactone oxidase | ALO1 | CaO19.7551 | O93852 | 0.08 | 0.00 | biofilm unique |
| Nucleosome assembly protein 1 | NAP1 | orf19.7501 | Q5AAI8 | 0.08 | 0.00 | biofilm unique |
| Candidapepsin-5 | SAP5 | CaO19.13032 | P43094 | 1361.70 | 5.62 | 242.20 |
| Agglutinin-like protein 3 | ALS3 | CaO19.1816 | Q59L12 | 354.54 | 2.81 | 126.12 |
| Hyphally regulated cell wall protein 1 | HYR1 | CaO19.12440 | Q5AL03 | 296.70 | 4.22 | 70.36 |
| pH-regulated antigen | PRA1 | CaO19.10623 | P87020 | 408.40 | 9.01 | 45.34 |
| Op4p | OP4 | CAALFM_C113080WA | A0A1D8PFJ9 | 104.80 | 2.87 | 36.47 |
| Cell wall protein | RBT1 | CaO19.1327 | Q59TP1 | 79.39 | 2.81 | 28.24 |
| Repressed by TUP1 protein 5 | RBT5 | CaO19.13081 | Q59UT4 | 32.14 | 1.41 | 22.87 |
| Putative aspartic endopeptidase | YPS7 | CAALFM_C702300WA | Q5AH56 | 31.33 | 1.41 | 22.29 |
| Extracellular glycosidase | UTR2 | CaO19.1671 | Q5AJC0 | 27.01 | 1.41 | 19.22 |
| Agglutinin-like protein 1 | ALS1 | CaO19.13163 | Q5A8T4 | 26.57 | 1.41 | 18.91 |
| Uncharacterized protein |  | CAALFM_C405580CA | Q5A470 | 173.17 | 9.26 | 18.69 |
| Beta-hexosaminidase | HEX1 | CAALFM_C503610WA | A0A1D8PNR7 | 50.95 | 2.87 | 17.73 |
| Secreted protein | RBT4 | CaO19.13583 | Q5AB48 | 89.46 | 5.17 | 17.30 |
| Exportin | CRM1 | CAALFM_CR00520CA | A0A1D8PRR9 | 9.01 | 0.57 | 15.68 |
| Cofilin | COF1 | CAALFM_C500370WA | A0A1D8PMW6 | 8.83 | 0.57 | 15.37 |
| Uncharacterized protein |  | CAALFM_C105890WA | A0A1D8PDR3 | 88.98 | 6.90 | 12.90 |
| Predicted GPI-anchored protein 45 | PGA45 | CaO19.2451 | Q5AA33 | 17.04 | 1.41 | 12.12 |
| Glyoxalase 3 | GLX3 | CaO19.251 | Q5AF03 | 33.69 | 2.81 | 11.99 |
| Adenylosuccinate lyase | ADE13 | CAALFM_CR06150CA | A0A1D8PT56 | 6.37 | 0.57 | 11.09 |
| 1,4-alpha-glucan branching enzyme | GLC3 | CAALFM_C603340CA | A0A1D8PQ59 | 5.95 | 0.57 | 10.35 |
| Coi1p | COI1 | CAALFM_C107900WA | Q5A799 | 14.34 | 1.41 | 10.20 |
| Protein OS-9 homolog | YOS9 | CaO19.7578 | Q5ACR4 | 13.34 | 1.41 | 9.49 |
| Fructose 1,6-bisphosphate 1-phosphatase | FBP1 | CAALFM_C307830WA | A0A1D8PKW2 | 5.29 | 0.57 | 9.20 |
| Putative methyltransferase | HRT2 | CAALFM_C401690CA | A0A1D8PLC6 | 5.05 | 0.57 | 8.78 |
| Alpha-1,2-mannosyltransferase | MNN24 | CaO19.1995 | Q5AD72 | 11.50 | 1.41 | 8.18 |
| pH-responsive protein 1 | PHR1 | CaO19.11310 | P43076 | 180.03 | 25.30 | 7.12 |
| Lysophospholipase | PLB3 | CAALFM_CR09690CA | A0A1D8PU17 | 7.78 | 1.15 | 6.77 |
| Bifunctional (2E,6E)-farnesyl diphosphate synthase/dimethylallyltranstransferase | ERG20 | CAALFM_C204580WA | A0A1D8PH78 | 3.88 | 0.57 | 6.75 |
| Acetyl-CoA C-acetyltransferase | ERG10 | CAALFM_C204310WA | A0A1D8PH52 | 7.12 | 1.15 | 6.20 |
| 6-phosphogluconate dehydrogenase, decarboxylating | GND1 | CAALFM_C113860CA | A0A1D8PFS4 | 56.95 | 9.20 | 6.19 |
| Asm3p | ASM3 | CAALFM_C100680WA | Q5ABD3 | 41.64 | 7.03 | 5.93 |
| Tos1p | TOS1 | CAALFM_C301550CA | A0A1D8PJA8 | 111.69 | 18.97 | 5.89 |
| Covalently-linked cell wall protein 14 | SSR1 | CaO19.7030 | Q5AFN8 | 10.68 | 1.98 | 5.39 |
| Agglutinin-like protein 4 | ALS4 | orf19.4555 | A0A1D8PQB9 | 9.01 | 1.72 | 5.23 |
| ER oxidoreductin | ERO1 | CAALFM_C110040WA | A0A1D8PER8 | 8.85 | 1.72 | 5.13 |
| E2 ubiquitin-conjugating protein | UBC4 | CAALFM_CR09970WA | A0A1D8PU40 | 5.83 | 1.15 | 5.07 |
| Pst1p | PST1 | CAALFM_C206870CA | A0A1D8PHR5 | 5.81 | 1.15 | 5.05 |
| Het1p | HET1 | CAALFM_C600100CA | A0A1D8PPA5 | 22.92 | 4.60 | 4.98 |
| Extent of cell elongation protein 1 | ECE1 | CaO19.10882 | Q07730 | 36.47 | 7.60 | 4.80 |
| Isopentenyl-diphosphate delta-isomerase | IDI1 | CAALFM_C402280WA | A0A1D8PLI2 | 10.80 | 2.30 | 4.70 |
| Phosphoglucomutase | PGM2 | CAALFM_CR02820WA | A0A1D8PSA9 | 20.92 | 4.60 | 4.55 |
| Chitinase 2 | CHT2 | CaO19.11376 | P40953 | 91.98 | 20.51 | 4.49 |
| Pleiotropic ABC efflux transporter of multiple drugs | CDR1 | CaO19.13421 | Q5ANA3 | 6.15 | 1.41 | 4.37 |
| Hsp70 nucleotide exchange factor | FES1 | CaO19.11133 | Q59NN8 | 2.35 | 0.57 | 4.09 |
| GPI-anchored protein 52 | PGA52 | CaO19.1911 | Q59L72 | 5.73 | 1.41 | 4.07 |
| Fum12p | FUM12 | CAALFM_C307640CA | A0A1D8PKV4 | 4.37 | 1.15 | 3.80 |
| Uncharacterized protein |  | CAALFM_C701710WA | A0A1D8PQW4 | 2.12 | 0.57 | 3.68 |
| Candidapepsin-10 | SAP10 | CaO19.11320 | Q5A651 | 4.93 | 1.41 | 3.51 |
| Hch1p | HCH1 | CAALFM_C601860CA | A0A1D8PPR5 | 2.00 | 0.57 | 3.48 |
| Adenosylhomocysteinase | SAH1 | CaO19.11392 | P83783 | 32.77 | 9.84 | 3.33 |
| Rho family GTPase | RHO3 | CAALFM_C205030CA | A0A1D8PH96 | 1.90 | 0.57 | 3.30 |
| Uncharacterized protein |  | CAALFM_C103510CA | A0A1D8PD11 | 3.74 | 1.15 | 3.26 |
| Cell wall mannoprotein | PIR1 | CaO19.220 | Q59SF7 | 9.07 | 2.81 | 3.23 |
| Coatomer subunit beta' | SEC27 | CAALFM_C602260CA | A0A1D8PPV5 | 3.67 | 1.15 | 3.19 |
| Secreted beta-glucosidase | SUN41 | CaO19.11124 | Q59NP5 | 176.15 | 55.39 | 3.18 |
| Acyl-protein thioesterase 1 |  | CaO19.11723 | Q5AGD1 | 5.44 | 1.72 | 3.15 |
| High-affinity iron permease | FTR1 | CAALFM_C114130WA | A0A1D8PFV0 | 1.81 | 0.57 | 3.15 |
| Csh1p | CSH1 | CAALFM_C104020CA | Q59QH2 | 17.67 | 5.62 | 3.14 |
| Ornithine-oxo-acid transaminase | CAR2 | CAALFM_C400160CA | A0A1D8PL14 | 14.43 | 4.60 | 3.14 |
| Peptidyl-prolyl cis-trans isomerase (PPIase) | CYP5 | CAALFM_C306360CA | A0A1D8PKL0 | 10.66 | 3.45 | 3.09 |
| Extracellular glycosidase | CRH11 | CaO19.10221 | Q5AFA2 | 37.77 | 12.65 | 2.99 |
| Karyopherin beta |  | CAALFM_C102240WA | Q59VX7 | 4.16 | 1.41 | 2.96 |
| Alpha-mannosidase | AMS1 | CAALFM_C402360WA | Q5AF38 | 16.55 | 5.62 | 2.94 |
| Citrate synthase | CIT1 | CAALFM_CR03500WA | A0A1D8PSH3 | 15.00 | 5.17 | 2.90 |
| Metalloaminopeptidase | LAP41 | CAALFM_C302170CA | A0A1D8PJE0 | 9.81 | 3.45 | 2.84 |
| Cell wall acid trehalase | ATC1 | CaO19.13595 | Q5AAU5 | 15.92 | 5.62 | 2.83 |
| Ecm33p | ECM33 | CAALFM_C103190CA | A0A1D8PCY4 | 98.54 | 35.06 | 2.81 |
| Putative ammonium permease | FRP3 | CAALFM_C206680WA | A0A1D8PHP8 | 1.60 | 0.57 | 2.78 |
| Sfc1p | SFC1 | CAALFM_C504440CA | A0A1D8PNY6 | 1.59 | 0.57 | 2.76 |
| Proteasome endopeptidase complex | PRE6 | CAALFM_CR04550WA | A0A1D8PSS4 | 3.16 | 1.15 | 2.75 |
| Uncharacterized protein |  | CAALFM_CR03520CA | A0A1D8PSI3 | 1.54 | 0.57 | 2.68 |
| Proteasome endopeptidase complex | PRE5 | CAALFM_C704020CA | A0A1D8PRH6 | 1.54 | 0.57 | 2.67 |
| Superoxide dismutase | SOD3 | CAALFM_C700110WA | A0A1D8PQH5 | 4.60 | 1.72 | 2.67 |
| Corticosteroid-binding protein | CBP1 | CaO19.7323 | P31225 | 7.44 | 2.81 | 2.65 |
| Msb2p | MSB2 | CAALFM_C201780WA | A0A1D8PGF8 | 44.11 | 16.67 | 2.65 |
| Putative glucan endo-1\3-beta-D-glucosidase | SCW11 | CAALFM_C504110WA | A0A1D8PNW1 | 35.85 | 13.79 | 2.60 |
| Lysophospholipase | PLB4.5 | CAALFM_C201380WA | A0A1D8PGF0 | 50.69 | 19.54 | 2.59 |
| Sortilin | PEP1 | CAALFM_C112490WA | A0A1D8PFH5 | 11.70 | 4.60 | 2.54 |
| Scw4p | SCW4 | CAALFM_C102520WA | Q5AIB2 | 10.22 | 4.22 | 2.42 |
| 1,3-beta-glucanosyltransferase PGA4 | PGA4 | CaO19.11518 | Q5AJY5 | 31.81 | 13.22 | 2.41 |
| Glycolipid 2-alpha-mannosyltransferase 2 | MNT2 | CaO19.1663 | P46592 | 13.49 | 5.62 | 2.40 |
| Gca2p | GCA2 | CAALFM_C110550CA | A0A1D8PEW1 | 49.09 | 20.69 | 2.37 |
| Proteasome core particle subunit beta 3 | PUP3 | CAALFM_C703390CA | A0A1D8PRB6 | 2.71 | 1.15 | 2.36 |
| H(+)-transporting V1 sector ATPase subunit H | VMA13 | CAALFM_CR05080WA | A0A1D8PSX0 | 6.66 | 2.87 | 2.32 |
| Chorismate synthase | ARO2 | CAALFM_CR07710WA | A0A1D8PTK1 | 1.33 | 0.57 | 2.32 |
| Glutamate decarboxylase | GAD1 | CAALFM_C111660WA | A0A1D8PF79 | 16.95 | 7.47 | 2.27 |
| PRA1 family protein |  | CAALFM_C106480CA | A0A1D8PDT9 | 1.29 | 0.57 | 2.24 |
| Alpha-1,2-mannosyltransferase | MNN26 | CaO19.13984 | Q59R28 | 9.16 | 4.22 | 2.17 |
| Cell surface mannoprotein MP65 | MP65 | CaO19.1779 | Q59XX2 | 189.21 | 90.65 | 2.09 |
| Proteasome core particle subunit beta 1 | PRE3 | CAALFM_C305560WA | A0A1D8PK85 | 1.18 | 0.57 | 2.05 |
| Kre9p | KRE9 | CAALFM_C304180WA | Q5ANN9 | 2.69 | 1.41 | 1.91 |
| Uncharacterized protein |  | CAALFM_C300640WA | Q5A7S3 | 2.63 | 1.41 | 1.87 |
| Cell division control protein 10 | CDC10 | CaO19.548 | P39827 | 2.62 | 1.41 | 1.86 |
| Uncharacterized protein |  | CAALFM_C307470WA | A0A1D8PKU6 | 11.26 | 6.32 | 1.78 |
| Carboxypeptidase | CPY1 | CAALFM_C703360WA | A0A1D8PRC2 | 1.02 | 0.57 | 1.77 |
| Glutathione peroxidase |  | CAALFM_C600850WA | Q59WW7 | 7.44 | 4.22 | 1.76 |
| 5-methyltetrahydropteroyltriglutamate--homocysteine methyltransferase | MET6 | CaO19.10083 | P82610 | 21.54 | 12.65 | 1.70 |
| Proteasome endopeptidase complex | PRE9 | CAALFM_C303520CA | Q5AEB8 | 2.32 | 1.41 | 1.65 |
| Uncharacterized protein |  | CAALFM_C105630CA | A0A1D8PDL7 | 15.77 | 9.77 | 1.61 |
| Ifr2p | IFR2 | CAALFM_CR03280WA | A0A1D8PSE7 | 3.70 | 2.30 | 1.61 |
| Transaldolase | TAL1 | CAALFM_CR03720WA | Q5A017 | 36.12 | 22.49 | 1.61 |
| Glucose-6-phosphate isomerase | PGI1 | CaO19.11369 | P83780 | 24.51 | 15.46 | 1.59 |
| Proteasome regulatory particle base subunit | RPN1 | CAALFM_C113300CA | A0A1D8PFL7 | 2.73 | 1.72 | 1.58 |
| Protein transport protein SEC31 | PGA63 | CaO19.13598 | Q5AAU3 | 2.18 | 1.41 | 1.55 |
| Ras-like protein 1 | RAS1 | CaO19.1760 | Q59XU5 | 6.50 | 4.22 | 1.54 |
| Hsp70 family ATPase | KAR2 | CAALFM_C201120WA | A0A1D8PG96 | 20.06 | 13.22 | 1.52 |
| Rab GDP dissociation inhibitor | GDI1 | CAALFM_C114440CA | A0A1D8PFX8 | 5.16 | 3.45 | 1.50 |
| Proteasome core particle subunit beta 6 |  | CAALFM_C402470CA | A0A1D8PLK1 | 3.38 | 2.30 | 1.47 |
| 3-hydroxyanthranilate 3,4-dioxygenase | BNA1 | CaO19.3515 | Q59K86 | 1.99 | 1.41 | 1.42 |
| Phosphoserine phosphatase | SER2 | CAALFM_C202690WA | Q5A0H3 | 1.98 | 1.41 | 1.41 |
| E1 ubiquitin-activating protein | UBA1 | CAALFM_C306500WA | A0A1D8PKJ3 | 7.25 | 5.17 | 1.40 |
| Arf3p | ARF3 | CAALFM_C301470WA | A0A1D8PJ64 | 0.80 | 0.57 | 1.39 |
| ADP-ribosylation factor GTPase-activating protein | GLO3 | CAALFM_C300240CA | Q5A7M1 | 0.79 | 0.57 | 1.38 |
| 3,4-dihydroxy-2-butanone 4-phosphate synthase) | RIB3 | CaO19.12693 | Q5A3V6 | 3.87 | 2.81 | 1.38 |
| ADP-ribosylation factor | ARF1 | CaO19.13805 | P22274 | 7.04 | 5.17 | 1.36 |
| Ras family GTPase | RSR1 | CAALFM_CR02140WA | A0A1D8PS50 | 1.56 | 1.15 | 1.36 |
| Protein disulfide isomerase | PDI1 | CAALFM_C703250CA | A0A1D8PR99 | 7.60 | 5.75 | 1.32 |
| 14-3-3 protein homolog | BMH1 | CaO19.10532 | O42766 | 20.35 | 15.46 | 1.32 |
| Repressed by EFG1 protein 1) | RBE1 | CaO19.7218 | Q59ZX3 | 9.25 | 7.03 | 1.32 |
| Phosphatidylinositol 4-phosphate-binding protein | GGA2 | CAALFM_C103130CA | A0A1D8PCX6 | 1.51 | 1.15 | 1.31 |
| Translationally-controlled tumor protein homolog (TCTP) | TMA19 | CaO19.10778 | Q5A860 | 1.84 | 1.41 | 1.31 |
| Alkaline phosphatase | PHO8 | CAALFM_C110430WA | A0A1D8PEV2 | 2.25 | 1.72 | 1.30 |
| Malate synthase | MLS1 | CAALFM_C109690WA | Q5APD2 | 14.64 | 11.24 | 1.30 |
| 60S ribosomal protein L10a | RPL10A | CaO19.10969 | Q9UVJ4 | 16.45 | 12.65 | 1.30 |
| Phosphoribosylaminoimidazolesuccinocarboxamide synthase | ADE1 | CAALFM_CR00510CA | A0A1D8PRQ1 | 1.49 | 1.15 | 1.29 |
| Septin | CDC3 | CAALFM_C104210CA | A0A1D8PD83 | 1.48 | 1.15 | 1.29 |
| Leu42p | LEU42 | CAALFM_C209750WA | A0A1D8PIF8 | 2.22 | 1.72 | 1.29 |
| Peptidyl-prolyl cis-trans isomerase (PPIase) | CYP1 | CaJ7.0279 | P22011 | 16.26 | 12.65 | 1.29 |
| E3 ubiquitin-protein ligase | RSP5 | CAALFM_C208500WA | Q59Y21 | 1.77 | 1.41 | 1.26 |
| Ran GTPase-binding protein | YRB1 | CAALFM_CR00580WA | A0A1D8PRQ3 | 0.71 | 0.57 | 1.23 |
| Coatomer subunit gamma | SEC21 | CAALFM_C104830WA | A0A1D8PDE3 | 5.63 | 4.60 | 1.23 |
| Dag7p | DAG7 | CAALFM_C401010CA | Q5AMF9 | 1.69 | 1.41 | 1.20 |
| RNA-binding protein |  | CAALFM_C304590WA | A0A1D8PK11 | 1.35 | 1.15 | 1.18 |
| Uncharacterized protein |  | CAALFM_CR03020CA | Q5A1X9 | 1.64 | 1.41 | 1.17 |
| Flavodoxin-like fold family protein | PST3 | CAALFM_CR05390WA | A0A1D8PT02 | 5.36 | 4.60 | 1.17 |
| Thioredoxin peroxidase | AHP1 | CAALFM_C402410CA | Q5AF44 | 14.31 | 12.65 | 1.13 |
| Hsp70 family chaperone | LHS1 | CAALFM_C202760WA | A0A1D8PGU0 | 4.45 | 4.02 | 1.11 |
| Uncharacterized protein |  | CAALFM_C110170WA | Q5AP78 | 1.54 | 1.41 | 1.09 |
| Proteasome endopeptidase complex | PUP2 | CAALFM_CR06750CA | Q59PZ1 | 1.50 | 1.41 | 1.06 |
| Glutamate-5-semialdehyde dehydrogenase | PRO2 | CAALFM_C307220CA | Q5ADR2 | 2.09 | 1.98 | 1.06 |
| Copper transport protein | CTR1 | CaO19.11130 | Q59NP1 | 5.88 | 5.62 | 1.05 |
| Proteasome endopeptidase complex | PRE8 | CAALFM_CR09380WA | A0A1D8PTY6 | 2.39 | 2.30 | 1.04 |
| Transcriptional repressor | TUP1 | CaO19.13528 | P0CY34 | 2.92 | 2.81 | 1.04 |
| Candidapepsin-9 | SAP9 | CaO19.14190 | Q59SU1 | 13.07 | 12.65 | 1.03 |
| Reticulon-like protein |  | CAALFM_C404800WA | A0A1D8PM53 | 3.54 | 3.45 | 1.03 |
| Phosphomannomutase | PMM1 | CaO19.10454 | P31353 | 11.51 | 11.24 | 1.02 |
| Saccharopine dehydrogenase [NAD(+), L-lysine-forming] | LYS1 | CAALFM_C405320WA | A0A1D8PMA1 | 2.35 | 2.30 | 1.02 |
| Hypoxanthine phosphoribosyltransferase | HPT1 | CAALFM_C202740CA | A0A1D8PGN7 | 0.59 | 0.57 | 1.02 |
| Nucleoside diphosphate kinase | YNK1 | CAALFM_C502890WA | Q5AG68 | 11.43 | 11.24 | 1.02 |
| Pho114p | PHO114 | CAALFM_C402070WA | Q5AMR2 | 2.81 | 2.81 | 1.00 |
| Uncharacterized protein |  | CAALFM_C101300WA | Q5A915 | 1.39 | 1.41 | 0.99 |
| Isocitrate dehydrogenase [NAD] subunit, mitochondrial | IDH2 | CAALFM_C203080WA | A0A1D8PGS5 | 2.84 | 2.87 | 0.99 |
| Formate dehydrogenase | FDH1 | CAALFM_CR05170CA | Q59QN6 | 6.92 | 7.03 | 0.98 |
| Isocitrate dehydrogenase [NADP] | IDP2 | CAALFM_CR02360WA | A0A1D8PS79 | 6.78 | 6.90 | 0.98 |
| Fimbrin | SAC6 | CAALFM_C602730WA | A0A1D8PPY8 | 2.24 | 2.30 | 0.97 |
| Yim1p | YIM1 | CAALFM_C203750WA | Q5AHE9 | 1.35 | 1.41 | 0.96 |
| Omega-class glutathione transferase | ECM4 | CAALFM_CR02130WA | A0A1D8PS56 | 1.63 | 1.72 | 0.94 |
| Endo-1,3(4)-beta-glucanase 1 | ENG1 | CaO19.10584 | Q5AIR7 | 46.76 | 50.60 | 0.92 |
| Protein YOP1 |  | CAALFM_C208160CA | A0A1D8PI76 | 2.06 | 2.30 | 0.90 |
| Glucan 1,3-beta-glucosidase | XOG1 | Ca49C10.05 | P29717 | 38.73 | 43.25 | 0.90 |
| Protein SUR7 | SUR7 | CaO19.10917 | Q5A4M8 | 4.94 | 5.62 | 0.88 |
| Actin-related protein 2/3 complex subunit 4 | ARC19 | CAALFM_CR01040CA | A0A1D8PRU4 | 0.50 | 0.57 | 0.87 |
| Plasma membrane ATPase | PMA1 | CAALFM_C300720WA | A0A1D8PJ01 | 17.94 | 20.69 | 0.87 |
| Oxysterol-binding protein |  | CAALFM_C205720CA | A0A1D8PHG1 | 3.96 | 4.60 | 0.86 |
| Glucan 1,3-beta-glucosidase | BGL2 | CaO19.12034 | Q5AMT2 | 178.63 | 208.72 | 0.86 |
| Chitinase 1 | CHT1 | CaO19.7517 | Q5AAH2 | 7.17 | 8.43 | 0.85 |
| Cell division control protein 42 homolog | CDC42 | CaO19.390 | P0CY33 | 2.32 | 2.81 | 0.83 |
| Flavodoxin-like fold family protein | YCP4 | CAALFM_CR05380CA | A0A1D8PT03 | 0.94 | 1.15 | 0.82 |
| Glycolipid 2-alpha-mannosyltransferase 1 | MNT1 | CaO19.1665 | Q00310 | 20.71 | 25.30 | 0.82 |
| Coatomer subunit zeta |  | CAALFM_CR03600CA | Q5A006 | 2.30 | 2.81 | 0.82 |
| Glucoamylase 1 | GAM1 | CaO19.12365 | O74254 | 37.13 | 45.81 | 0.81 |
| Candidapepsin-8 | SAP8 | CaO19.242 | Q5AEM6 | 5.48 | 7.03 | 0.78 |
| Proteinase A | APR1 | CAALFM_C207400CA | Q59U59 | 5.43 | 7.03 | 0.77 |
| Endoplasmic reticulum vesicle protein 25 | ERV25 | CaO19.7409 | Q5A302 | 3.26 | 4.22 | 0.77 |
| Rab family GTPase | YPT1 | CAALFM_C103500WA | Q5AI00 | 4.26 | 5.62 | 0.76 |
| Cell wall protein RHD3 (LDG family protein 7) | RHD3 | CaO19.12765 | Q5A5U4 | 2.13 | 2.81 | 0.76 |
| ATP-dependent RNA helicase eIF4A | TIF1 | CaO19.10834 | P87206 | 31.37 | 41.91 | 0.75 |
| Translation elongation factor EF1B gamma | CAM1 | CAALFM_C306010WA | A0A1D8PKC3 | 10.65 | 14.37 | 0.74 |
| Protein phosphatase 2A structural subunit | TPD3 | CAALFM_C306910CA | Q5ADN1 | 4.03 | 5.62 | 0.72 |
| Mitochondrial outer membrane protein porin (Cytoplasmic antigenic protein 4) | POR1 | CaO19.1042 | P83781 | 18.12 | 25.30 | 0.72 |
| Arf family GTPase | ARF2 | CAALFM_C304950WA | Q5AND9 | 17.05 | 23.89 | 0.71 |
| Peptidyl-prolyl cis-trans isomerase D (PPIase D) | CPR6 | CaO19.7654 | Q5ACI8 | 2.97 | 4.22 | 0.70 |
| Peroxiredoxin TSA1-A | TSA1 | CaO19.7417 | Q9Y7F0 | 11.76 | 16.87 | 0.70 |
| Uncharacterized protein |  | CAALFM_C201690WA | Q5ALU6 | 0.98 | 1.41 | 0.70 |
| Glutamate dehydrogenase | GDH3 | CAALFM_C406120WA | A0A1D8PMH8 | 3.20 | 4.60 | 0.70 |
| Exomer complex subunit | BUD7 | CAALFM_C503820CA | Q59N76 | 0.96 | 1.41 | 0.69 |
| Cell surface GPI-anchored protein ECM33 | ECM331 | CaO19.11730 | Q5AGC4 | 0.96 | 1.41 | 0.68 |
| Uncharacterized protein |  | CAALFM_C202410WA | A0A1D8PGL1 | 1.56 | 2.30 | 0.68 |
| Mir1p | MIR1 | CAALFM_C110160WA | Q5AP79 | 15.20 | 22.49 | 0.68 |
| L-iditol 2-dehydrogenase | XYL2 | CAALFM_CR10840CA | A0A1D8PUB4 | 8.03 | 12.07 | 0.67 |
| Superoxide dismutase | SOD2 | CAALFM_C101520CA | Q5A8Z4 | 1.87 | 2.81 | 0.66 |
| GTP-binding protein RHO1 | RHO1 | CaO19.10362 | O42825 | 10.08 | 15.46 | 0.65 |
| Tubulin alpha chain | TUB1 | CAALFM_CR09120CA | A0A1D8PTV4 | 6.14 | 9.77 | 0.63 |
| Eukaryotic translation initiation factor 4E | TIF45 | CaO19.7626 | Q9P975 | 1.71 | 2.81 | 0.61 |
| Transcription factor TFIIIC subunit |  | CAALFM_C701650WA | A0A1D8PQW6 | 1.05 | 1.72 | 0.61 |
| Mrf1p | MRF1 | CAALFM_C111700CA | Q59TU5 | 2.55 | 4.22 | 0.60 |
| Chitinase 3 | CHT3 | CaO19.7586 | P40954 | 27.70 | 45.81 | 0.60 |
| Phosphoglycerate kinase | PGK1 | CaO19.11135 | P46273 | 39.13 | 65.23 | 0.60 |
| Protein transport protein | SEC13 | CaO19.316 | Q5AEF2 | 1.68 | 2.81 | 0.60 |
| Actin | ACT1 | CAALFM_C113700WA | A0A1D8PFR4 | 13.69 | 22.99 | 0.60 |
| Phosphoacetylglucosamine mutase (PAGM) | AGM1 | CAALFM_C113760WA | Q5AKW4 | 1.65 | 2.81 | 0.59 |
| NAD-specific glutamate dehydrogenase | GDH2 | CAALFM_C207900WA | A0A1D8PI00 | 3.36 | 5.75 | 0.58 |
| pH-responsive protein 2 | PHR2 | CaO19.13500 | O13318 | 4.92 | 8.43 | 0.58 |
| Ribosomal 60S subunit protein L12A | RPL12 | CAALFM_C302110WA | Q5AJF7 | 6.04 | 10.41 | 0.58 |
| Alcohol dehydrogenase 2 | ADH2 | Ca41C10.04 | O94038 | 13.27 | 23.06 | 0.58 |
| Candidapepsin-2 | SAP2 | CaO19.11193 | P0DJ06 | 5.94 | 10.41 | 0.57 |
| Sla2p | SLA2 | CAALFM_C703810WA | A0A1D8PRF5 | 0.33 | 0.57 | 0.57 |
| Mannose-6-phosphate isomerase | PMI1 | CaO19.1390 | P34948 | 2.70 | 4.79 | 0.56 |
| S-adenosylmethionine synthase | SAM2 | CAALFM_C111450CA | A0A1D8PF68 | 6.45 | 11.49 | 0.56 |
| Guanine nucleotide-binding protein subunit beta-like protein (Cytoplasmic antigenic protein 1) | ASC1 | CaJ7.0140 | P83774 | 22.00 | 39.36 | 0.56 |
| Uncharacterized protein |  | CAALFM_CR00090CA | A0A1D8PRL3 | 0.31 | 0.57 | 0.55 |
| Proteasome core particle subunit beta 5 | PRE2 | CAALFM_C206820CA | Q59Z65 | 1.53 | 2.81 | 0.54 |
| S-(hydroxymethyl)glutathione dehydrogenase | FDH3 | CAALFM_CR10250CA | A0A1D8PU61 | 4.96 | 9.20 | 0.54 |
| Phosphoenolpyruvate carboxykinase | PCK1 | CAALFM_CR00200WA | A0A1D8PRM7 | 12.53 | 24.14 | 0.52 |
| Proliferating cell nuclear antigen | POL30 | CAALFM_C401770WA | Q5AMN0 | 2.18 | 4.22 | 0.52 |
| 3-hydroxy-3-methylglutaryl coenzyme A synthase (HMG-CoA synthase) | ERG13 | CAALFM_CR09160CA | A0A1D8PTW6 | 3.52 | 6.90 | 0.51 |
| Ribosomal 60S subunit protein L14B | RPL14 | CAALFM_C113050WA | A0A1D8PFL9 | 3.47 | 6.90 | 0.50 |
| Cam1-1p | CAM1-1 | CAALFM_C503280WA | A0A1D8PNN8 | 5.70 | 11.49 | 0.50 |
| Adenylyl cyclase-associated protein | SRV2 | CAALFM_CR04190WA | Q5A6P9 | 2.08 | 4.22 | 0.49 |
| Phosphoglycerate mutase (PGAM) | GPM1 | CaO19.8522 | P82612 | 8.91 | 18.27 | 0.49 |
| Inorganic pyrophosphatase | IPP1 | CaO19.11072 | P83777 | 7.39 | 15.46 | 0.48 |
| Heat shock protein 90 homolog | HSP90 | CaJ7.0234 | P46598 | 38.73 | 82.61 | 0.47 |
| Emp24p | EMP24 | CAALFM_CR07590WA | Q59V63 | 1.96 | 4.22 | 0.46 |
| Hexokinase-2 | HXK2 | CaO19.542 | P83776 | 22.87 | 50.34 | 0.45 |
| Secreted beta-glucosidase | SIM1 | CaO19.12499 | Q5AKU5 | 35.33 | 77.88 | 0.45 |
| Rab family GTPase | YPT31 | CAALFM_CR07520CA | A0A1D8PTI2 | 0.78 | 1.72 | 0.45 |
| Metalloendopeptidase | PRD1 | CAALFM_C105300CA | Q5A2A7 | 3.75 | 8.43 | 0.45 |
| SCF ubiquitin ligase subunit | SKP1 | CAALFM_C107410CA | Q59WE2 | 0.62 | 1.41 | 0.44 |
| 60S acidic ribosomal protein P0 | RPP0 | CAALFM_C700990WA | A0A1D8PQS0 | 3.05 | 6.90 | 0.44 |
| Isocitrate dehydrogenase [NAD] subunit, mitochondrial | IDH1 | CAALFM_C109630WA | A0A1D8PEM5 | 1.51 | 3.45 | 0.44 |
| Tubulin beta chain | TUB2 | CAALFM_C100710CA | A0A1D8PC97 | 3.53 | 8.05 | 0.44 |
| Triosephosphate isomerase | TPI1 | CaO19.14037 | Q9P940 | 33.08 | 75.90 | 0.44 |
| Trifunctional fatty acid synthase subunit | FAS2 | CAALFM_C304830CA | A0A1D8PK65 | 21.03 | 48.85 | 0.43 |
| 1,3-beta-glucan synthase | GSC1 | CAALFM_C102420CA | A0A1D8PCT0 | 4.68 | 10.92 | 0.43 |
| Putative cystathionine beta-lyase |  | CAALFM_CR09010CA | A0A1D8PTV7 | 1.47 | 3.45 | 0.43 |
| SEC14 cytosolic factor (Phosphatidylinositol/phosphatidylcholine transfer protein) | SEC14 | CaO19.8556 | P46250 | 2.39 | 5.62 | 0.42 |
| DNA mismatch repair protein HSM3 | HSM3 | CaJ7.0396 | Q59PP0 | 2.10 | 5.05 | 0.42 |
| UTP--glucose-1-phosphate uridylyltransferase | UGP1 | CaO19.1738 | Q59KI0 | 5.39 | 13.22 | 0.41 |
| D-arabinose 1-dehydrogenase (NAD(P)(+)) |  | CAALFM_C400380WA | A0A1D8PL15 | 0.23 | 0.57 | 0.41 |
| Mannan endo-1,6-alpha-mannosidase | DFG5 | CaO19.2075 | Q5ACZ2 | 1.70 | 4.22 | 0.40 |
| Nucleoporin |  | CAALFM_C104200CA | A0A1D8PD79 | 3.71 | 9.20 | 0.40 |
| 40S ribosomal protein S12 | RPS12 | CAALFM_C307150CA | Q5ADQ6 | 2.83 | 7.03 | 0.40 |
| Saccharopine dehydrogenase (NADP+, L-glutamate-forming) | LYS9 | CAALFM_C306590WA | A0A1D8PKJ4 | 2.08 | 5.17 | 0.40 |
| Phm7p | PHM7 | CAALFM_C208140CA | A0A1D8PI19 | 0.23 | 0.57 | 0.40 |
| Succinate--CoA ligase [ADP-forming] subunit alpha, mitochondrial | LSC1 | CAALFM_C101690CA | Q5A8X6 | 3.36 | 8.43 | 0.40 |
| Dihydrolipoyl dehydrogenase | LPD1 | CAALFM_CR07400CA | Q59RQ6 | 2.18 | 5.62 | 0.39 |
| Elongation factor 2 | EFT2 | CaO19.13210 | Q5A0M4 | 50.63 | 132.76 | 0.38 |
| Uncharacterized protein |  | CAALFM_CR07460CA | A0A1D8PTI1 | 7.65 | 20.12 | 0.38 |
| ADP/ATP carrier protein | PET9 | CAALFM_C500590WA | Q5A516 | 24.37 | 66.64 | 0.37 |
| Fructose-bisphosphate aldolase | FBA1 | CaO19.12088 | Q9URB4 | 39.11 | 107.08 | 0.37 |
| Bifunctional cysteine synthase/O-acetylhomoserine aminocarboxypropyltransferase | MET15 | CAALFM_C400200CA | Q59US5 | 2.55 | 7.03 | 0.36 |
| Adh1p | ADH1 | CAALFM_C505050WA | A0A1D8PP43 | 24.86 | 69.54 | 0.36 |
| Hsp90 co-chaperone | CDC37 | CaO19.5531 | Q8X1E6 | 0.50 | 1.41 | 0.36 |
| Pyruvate kinase | CDC19 | CaO19.11059 | P46614 | 13.15 | 37.12 | 0.35 |
| GTP-binding nuclear protein | GSP1 | CAALFM_C206310CA | Q59P43 | 3.68 | 10.41 | 0.35 |
| Asparagine--tRNA ligase | DED81 | CAALFM_C703670WA | Q59R18 | 3.92 | 11.24 | 0.35 |
| Acetyl-coenzyme A synthetase | ACS1 | CAALFM_C210350CA | Q59XW4 | 5.06 | 14.63 | 0.35 |
| Transketolase | TKL1 | CAALFM_C108320WA | Q5A750 | 11.28 | 34.31 | 0.33 |
| Histone H4 | HHF1 | CAALFM_C104240CA | Q59VN4 | 1.37 | 4.22 | 0.33 |
| F1F0 ATP synthase subunit 4 | ATP4 | CAALFM_C205500WA | Q59ZE0 | 2.72 | 8.43 | 0.32 |
| Ardp | ARD | CAALFM_C600150WA | A0A1D8PPB1 | 0.55 | 1.72 | 0.32 |
| 60S ribosomal protein L6 | RPL6 | CAALFM_C103110WA | A0A1D8PCX8 | 2.59 | 8.05 | 0.32 |
| Isocitrate lyase | ICL1 | CAALFM_C104500WA | Q59RB8 | 4.45 | 14.06 | 0.32 |
| Ribosomal 60S subunit protein L22B |  | CAALFM_C404390WA | A0A1D8PM41 | 1.26 | 4.02 | 0.31 |
| Dolichyl-diphosphooligosaccharide--protein glycosyltransferase subunit | WBP1 | CAALFM_C111170WA | A0A1D8PF22 | 0.17 | 0.57 | 0.30 |
| Non-classical export protein 102 | NCE102 | CaO19.13381 | Q5ANE3 | 1.70 | 5.62 | 0.30 |
| UDP-N-acetylglucosamine diphosphorylase | UAP1 | CAALFM_C502530WA | A0A1D8PNG6 | 0.17 | 0.57 | 0.30 |
| Ribosomal 40S subunit protein S18B | RPS18 | CAALFM_C700960WA | A0A1D8PQQ5 | 2.89 | 9.77 | 0.30 |
| Enolase 1 | ENO1 | CaO19.395 | P30575 | 53.48 | 180.80 | 0.30 |
| Homocitrate synthase | LYS21 | CAALFM_C104730CA | Q59VG1 | 5.37 | 18.27 | 0.29 |
| H(+)-transporting V1 sector ATPase subunit E | VMA4 | CAALFM_CR01970CA | A0A1D8PS38 | 0.34 | 1.15 | 0.29 |
| ATP synthase subunit d, mitochondrial | ATP7 | CAALFM_C107600WA | Q59PV8 | 2.06 | 7.03 | 0.29 |
| Anthranilate phosphoribosyltransferase | TRP4 | CAALFM_C407090CA | A0A1D8PMR2 | 0.64 | 2.30 | 0.28 |
| Importin subunit alpha |  | CAALFM_C500150CA | A0A1D8PMU8 | 0.96 | 3.45 | 0.28 |
| Glutathione peroxidase | GPX2 | CAALFM_C600840WA | Q59WW8 | 0.78 | 2.81 | 0.28 |
| Bifunctional AP-4-A phosphorylase/ADP sulfurylase | APA2 | CAALFM_C603410CA | A0A1D8PQ57 | 0.16 | 0.57 | 0.27 |
| ATP-binding cassette glutathione S-conjugate transporter | YCF1 | CAALFM_C702330WA | A0A1D8PR42 | 0.16 | 0.57 | 0.27 |
| Aspartyl aminopeptidase |  | CAALFM_C110820CA | Q59WG6 | 2.04 | 7.60 | 0.27 |
| Clathrin heavy chain (Fragment) | CHC1 | CAALFM_C602120WA | A0A1D8PPS9 | 2.78 | 10.35 | 0.27 |
| Ubiquitin-ribosomal 40S subunit protein S31 fusion protein | UBI3 | CAALFM_C407180WA | Q5A109 | 12.18 | 45.55 | 0.27 |
| Ftr2p | FTR2 | CAALFM_C114220CA | A0A1D8PFV2 | 0.15 | 0.57 | 0.27 |
| Uncharacterized protein |  | CAALFM_C209970CA | Q59YH1 | 1.45 | 5.62 | 0.26 |
| Ketol-acid reductoisomerase, mitochondrial | ILV5 | CAALFM_C600870CA | A0A1D8PPG7 | 1.44 | 5.75 | 0.25 |
| Acyl-coenzyme A oxidase | POX1-3 | CAALFM_C301960CA | Q5AJD9 | 5.36 | 21.66 | 0.25 |
| Sphingolipid C9-methyltransferase (C-9-MT) | MTS1 | CaO19.12294 | Q5APD4 | 1.87 | 7.60 | 0.25 |
| Ribosomal 40S subunit protein S23B | RPS23A | CAALFM_C106580WA | A0A1D8PDU3 | 0.56 | 2.30 | 0.24 |
| Peroxisomal catalase | CAT1 | CaO19.13609 | O13289 | 3.24 | 13.61 | 0.24 |
| Proteasome core particle subunit beta 2 | PUP1 | CAALFM_CR10300WA | A0A1D8PU67 | 0.27 | 1.15 | 0.24 |
| NADH-cytochrome b5 reductase 2 | MCR1 | CaO19.11001 | Q59M70 | 3.27 | 14.06 | 0.23 |
| Eukaryotic translation initiation factor 5A | ANB1 | CaO19.10930 | O94083 | 1.89 | 8.43 | 0.22 |
| Uncharacterized protein |  | CAALFM_C113190WA | A0A1D8PFM0 | 0.26 | 1.15 | 0.22 |
| Alpha-1,2-mannosyltransferase | MNN2 | CaO19.2347 | Q59WF4 | 2.17 | 9.84 | 0.22 |
| Tetrafunctional fatty acid synthase subunit | FAS1 | CAALFM_C500190CA | Q5A4W7 | 27.76 | 127.65 | 0.22 |
| AMP-activated serine/threonine-protein kinase regulatory subunit | SNF4 | CAALFM_C603920WA | A0A1D8PQA6 | 0.25 | 1.15 | 0.22 |
| Ribosomal 40S subunit protein S9B | RPS9B | CAALFM_C203820CA | A0A1D8PGY8 | 1.48 | 6.90 | 0.22 |
| Ribosomal 40S subunit protein S16A | RPS16A | CAALFM_C103030WA | A0A1D8PCW6 | 1.44 | 6.90 | 0.21 |
| Asparagine synthase (Glutamine-hydrolyzing) 2 | ASN1 | CAALFM_C209060CA | A0A1D8PIB2 | 2.37 | 11.49 | 0.21 |
| Alpha,alpha-trehalose-phosphate synthase [UDP-forming] | TPS1 | CaO19.13961 | Q92410 | 1.68 | 8.43 | 0.20 |
| Phosphotransferase | GLK1 | CAALFM_CR07150WA | Q59TZ8 | 6.19 | 31.50 | 0.20 |
| Elongation factor 1-alpha 2 (EF-1-alpha 2) | TEF2 | CaO19.382 | Q59QD6 | 23.54 | 119.86 | 0.20 |
| Ribosomal 60S subunit protein L18A | RPL18 | CAALFM_C305100CA | A0A1D8PK43 | 1.44 | 7.47 | 0.19 |
| Psa2p | PSA2 | CAALFM_C113160WA | Q5AL34 | 2.69 | 14.06 | 0.19 |
| Ras-related protein SEC4 | SEC4 | Ca49C4.03c | P0CY31 | 1.60 | 8.43 | 0.19 |
| Protein transport protein SEC23 | SEC23 | CaO19.1254 | Q5A455 | 1.71 | 9.01 | 0.19 |
| Pyridoxine biosynthesis protein | SNZ1 | CAALFM_C102590CA | Q5AIA6 | 2.67 | 14.63 | 0.18 |
| Protein transport protein SEC24 | SEC24 | CaO19.12194 | Q5AQ76 | 1.98 | 11.24 | 0.18 |
| Trehalose 6-phosphate synthase/phosphatase complex subunit | TPS3 | CAALFM_C210690WA | A0A1D8PIS4 | 0.60 | 3.45 | 0.17 |
| Uncharacterized protein |  | CAALFM_C202000WA | A0A1D8PGG5 | 0.69 | 4.02 | 0.17 |
| 40S ribosomal protein S0 | RPS0 | CaO19.6975 | O42817 | 5.24 | 30.67 | 0.17 |
| Uncharacterized protein |  | CAALFM_C108110WA | A0A1D8PEA9 | 0.10 | 0.57 | 0.17 |
| Nuclear pore protein |  | CAALFM_C201220WA | A0A1D8PGB0 | 0.10 | 0.57 | 0.17 |
| Alpha-1,4 glucan phosphorylase | GPH1 | CAALFM_C700930WA | A0A1D8PQQ3 | 6.09 | 36.21 | 0.17 |
| F1F0 ATP synthase subunit e |  | CAALFM_C400330CA | A0A1D8PL02 | 0.19 | 1.15 | 0.17 |
| Putative hydrolase | NIT3 | CAALFM_C110700CA | Q59WF0 | 0.23 | 1.41 | 0.17 |
| Transcription elongation factor | SPT6 | CaJ7.0509 | Q3MNT0 | 0.10 | 0.57 | 0.17 |
| Homoserine dehydrogenase | HOM6 | CAALFM_C102620CA | Q5AIA2 | 2.56 | 15.46 | 0.17 |
| 40S ribosomal protein S22-B | RPS22B | orf19.3061.1 | P0CU35 | 2.75 | 16.87 | 0.16 |
| Heat shock protein SSA1 | SSA1 | CaO19.12447 | P41797 | 16.75 | 103.18 | 0.16 |
| Isoleucine--tRNA ligase | ILS1 | CAALFM_C604520WA | Q59RI1 | 6.97 | 43.57 | 0.16 |
| Uncharacterized protein |  | CAALFM_C502380WA | A0A1D8PNE5 | 0.63 | 4.02 | 0.16 |
| Pyruvate decarboxylase | PDC11 | CaO19.10395 | P83779 | 35.27 | 227.76 | 0.15 |
| Ubiquinol--cytochrome-c reductase subunit |  | CAALFM_C505230CA | A0A1D8PP59 | 1.50 | 9.77 | 0.15 |
| ATP synthase subunit alpha | ATP1 | CAALFM_C104610WA | A0A1D8PDC4 | 2.16 | 14.37 | 0.15 |
| 40S ribosomal protein S21 | RPS21B | CAALFM_C101370CA | A0A1D8PCG7 | 0.17 | 1.15 | 0.15 |
| S-adenosylmethionine-homocysteine S-methyltransferase | SAM4 | CAALFM_C108410CA | A0A1D8PEB0 | 0.34 | 2.30 | 0.15 |
| Transmembrane 9 superfamily member | EMP70 | CAALFM_C402550CA | Q5AF62 | 0.50 | 3.39 | 0.15 |
| Thioredoxin peroxidase | PRX1 | CAALFM_C702810WA | Q5A5A0 | 1.43 | 9.84 | 0.15 |
| Fmp45p | FMP45 | CAALFM_C702240WA | A0A1D8PR25 | 0.33 | 2.30 | 0.14 |
| Cell wall protein | IFF11 | CaO19.12854 | Q5A7R7 | 2.20 | 15.46 | 0.14 |
| Uncharacterized protein |  | CAALFM_C504470CA | Q5AK88 | 0.39 | 2.81 | 0.14 |
| Acetyltransferase component of pyruvate dehydrogenase complex | LAT1 | CAALFM_C701640WA | Q5AGX8 | 2.52 | 18.27 | 0.14 |
| Bifunctional aminoimidazole ribotide synthase/glycinamide ribotide synthase | ADE5,7 | CAALFM_C107890CA | A0A1D8PE67 | 0.63 | 4.60 | 0.14 |
| Carnitine O-acetyltransferase | CAT2 | CAALFM_C402020WA | Q5AMQ5 | 0.58 | 4.22 | 0.14 |
| Dipeptidyl peptidase 3 |  | CAALFM_C603960WA | A0A1D8PQB4 | 0.16 | 1.15 | 0.14 |
| Isocitrate dehydrogenase [NADP] | IDP1 | CAALFM_C205890CA | A0A1D8PHH7 | 0.23 | 1.72 | 0.14 |
| Age3p | AGE3 | CAALFM_C102260CA | A0A1D8PCP6 | 0.08 | 0.57 | 0.13 |
| Peptidyl-prolyl cis-trans isomerase (PPIase) | CPR3 | CAALFM_C202320CA | Q5ALM6 | 0.08 | 0.57 | 0.13 |
| Uncharacterized protein |  | CAALFM_C202540WA | A0A1D8PGR5 | 0.08 | 0.57 | 0.13 |
| Histidine biosynthesis trifunctional protein | HIS4 | CAALFM_C400140CA | A0A1D8PKY7 | 0.08 | 0.57 | 0.13 |
| Uncharacterized protein |  | CAALFM_C400410WA | Q59UQ1 | 0.08 | 0.57 | 0.13 |
| Fre7p | FRE7 | CAALFM_CR07290WA | A0A1D8PTG2 | 0.08 | 0.57 | 0.13 |
| Ribosomal 40S subunit protein S3 | RPS3 | CAALFM_CR04810WA | A0A1D8PSV5 | 2.03 | 15.52 | 0.13 |
| Histone H2A.Z | HTZ1 | CaO19.327 | Q5AEE1 | 0.18 | 1.41 | 0.13 |
| Hexose transporter | HGT6 | CAALFM_C201020WA | Q5AD47 | 1.25 | 9.84 | 0.13 |
| Aspartate aminotransferase | AAT21 | CAALFM_CR07620WA | Q59N40 | 0.70 | 5.62 | 0.13 |
| Osm1p | OSM1 | CAALFM_C205700WA | Q59T35 | 1.22 | 9.84 | 0.12 |
| Very-long-chain 3-oxoacyl-CoA reductase |  | CaO19.11340 | Q59V93 | 0.17 | 1.41 | 0.12 |
| Acetyl-CoA hydrolase | ACH1 | CaO19.10681 | P83773 | 2.14 | 17.44 | 0.12 |
| Erv29p | ERV29 | CAALFM_C402120WA | Q5AMR7 | 0.17 | 1.41 | 0.12 |
| Carnitine:acyl carnitine antiporter | CRC1 | CAALFM_CR01980CA | Q5A967 | 0.17 | 1.41 | 0.12 |
| Pyruvate carboxylase | PYC2 | CAALFM_C403940CA | A0A1D8PLY4 | 1.85 | 15.52 | 0.12 |
| ATP-dependent RNA helicase | SUB2 | CAALFM_C400220CA | A0A1D8PKZ3 | 1.09 | 9.20 | 0.12 |
| Cys-Gly metallodipeptidase | DUG1 | CaO19.11397 | Q5AKA5 | 2.90 | 24.73 | 0.12 |
| Aspartate transaminase | AAT1 | CAALFM_C205250CA | A0A1D8PHC9 | 0.87 | 7.47 | 0.12 |
| Acetyl-coenzyme A synthetase 2 | ACS2 | CaO19.1064 | Q8NJN3 | 3.68 | 32.90 | 0.11 |
| Uncharacterized protein |  | CAALFM_C100700WA | Q5ABD5 | 0.16 | 1.41 | 0.11 |
| 4-nitrophenylphosphatase (PNPPase) | PHO15 | CAALFM_C107230WA | Q59WC5 | 0.16 | 1.41 | 0.11 |
| Histone H2B.2 | HTB2 | CaO19.1052 | Q59VP1 | 0.76 | 7.03 | 0.11 |
| Ribosomal 60S subunit protein L9B | RPL9B | CAALFM_C302470CA | Q5AEN2 | 2.32 | 21.66 | 0.11 |
| AAA family ATPase | SEC18 | CAALFM_C113580WA | A0A1D8PFN6 | 0.18 | 1.72 | 0.10 |
| Translation initiation factor eIF2 subunit gamma | GCD11 | CAALFM_C502170CA | Q5AGF6 | 1.28 | 12.65 | 0.10 |
| Ribonucleotide-diphosphate reductase subunit | RNR21 | CAALFM_C203010CA | Q5A0L0 | 0.56 | 5.62 | 0.10 |
| 6,7-dimethyl-8-ribityllumazine synthase (DMRL synthase) | RIB4 | CAALFM_C105560WA | A0A1D8PDK6 | 0.17 | 1.72 | 0.10 |
| Csp37p | CSP37 | CAALFM_CR01470WA | Q5A9D4 | 0.80 | 8.43 | 0.10 |
| Bifunctional 2-aminoadipate transaminase/aromatic-amino-acid:2-oxoglutarate transaminase | ARO8 | CAALFM_C200340CA | A0A1D8PG20 | 0.63 | 6.90 | 0.09 |
| Phospho-2-dehydro-3-deoxyheptonate aldolase | ARO3 | CAALFM_C202030WA | A0A1D8PGI8 | 0.16 | 1.72 | 0.09 |
| 60S ribosomal protein L36 | RPL39 | CAALFM_C203960WA | A0A1D8PH21 | 0.16 | 1.72 | 0.09 |
| Uncharacterized protein |  | CAALFM_C304650WA | Q5ANH8 | 0.37 | 4.22 | 0.09 |
| Succinate--CoA ligase [ADP-forming] subunit beta, mitochondrial | LSC2 | CAALFM_CR06760CA | A0A1D8PTB5 | 0.69 | 8.05 | 0.09 |
| Acid phosphatase | PHO113 | CAALFM_CR02180WA | Q59UY6 | 2.94 | 34.31 | 0.09 |
| F1F0 ATP synthase subunit i | ATP18 | CAALFM_C200610CA | A0A1D8PG50 | 0.10 | 1.15 | 0.08 |
| 40S ribosomal protein S27 | RPS27 | CAALFM_CR07630CA | A0A1D8PTI7 | 0.10 | 1.15 | 0.08 |
| Chaperone ATPase | HSP104 | CAALFM_CR08250CA | A0A1D8PTP9 | 1.36 | 16.67 | 0.08 |
| Uncharacterized protein |  | CAALFM_C101490WA | Q5A8Z9 | 0.56 | 7.03 | 0.08 |
| Ribosomal 60S subunit protein L7A |  | CAALFM_C105720WA | A0A1D8PDL6 | 0.94 | 12.07 | 0.08 |
| Cytochrome c oxidase subunit 1 | COX1 | CaalfMp08 | P0C8K9 | 0.33 | 4.22 | 0.08 |
| Mannose-1-phosphate guanyltransferase | MPG1 | CaO19.6190 | O93827 | 2.04 | 26.71 | 0.08 |
| Elongation factor 3 (EF-3) | CEF3 | CaO19.11629 | P25997 | 9.35 | 122.60 | 0.08 |
| Cytochrome c oxidase subunit Va | COX5 | CAALFM_C109030CA | Q5APK5 | 0.63 | 8.43 | 0.08 |
| Png2p | PNG2 | CAALFM_C205660WA | A0A1D8PHH2 | 0.17 | 2.30 | 0.07 |
| Glyceraldehyde-3-phosphate dehydrogenase | TDH3 | CAALFM_C306870WA | Q5ADM7 | 20.32 | 277.59 | 0.07 |
| ATP-dependent 6-phosphofructokinase (ATP-PFK) | PFK2 | CAALFM_C701800CA | Q5AGZ8 | 2.13 | 30.35 | 0.07 |
| Rab family GTPase | VPS21 | CAALFM_CR08060CA | Q59X89 | 0.16 | 2.24 | 0.07 |
| Replication protein A subunit | RFA1 | CAALFM_C200380CA | Q5ACX4 | 0.10 | 1.41 | 0.07 |
| V-type proton ATPase subunit a | VPH1 | CAALFM_C405240CA | Q59R99 | 0.20 | 2.81 | 0.07 |
| Ribosomal 60S subunit protein L11B | RPL11 | CAALFM_C206810CA | A0A1D8PHW1 | 0.63 | 9.20 | 0.07 |
| Diphosphomevalonate decarboxylase | MVD | CAALFM_C100070WA | A0A1D8PC43 | 0.16 | 2.30 | 0.07 |
| Proteasome regulatory particle lid subunit | RPN5 | CAALFM_C505380WA | A0A1D8PP77 | 0.16 | 2.30 | 0.07 |
| Putative Rab GTPase-binding protein | SRO77 | CAALFM_C600410CA | Q59RK1 | 0.10 | 1.41 | 0.07 |
| Proteasome regulatory particle lid subunit |  | CAALFM_C201320WA | A0A1D8PGA6 | 0.19 | 2.87 | 0.07 |
| Serine/threonine-protein phosphatase | PPH21 | CAALFM_C301600WA | A0A1D8PJ80 | 0.15 | 2.30 | 0.07 |
| 40S ribosomal protein S4 | RPS4A | CAALFM_C210620WA | Q5A5Q8 | 1.61 | 24.47 | 0.07 |
| Adenylate kinase | ADK1 | CaO19.10894 | Q5A4Q1 | 0.64 | 9.84 | 0.07 |
| Putative mannosyltransferase | KTR4 | CAALFM_C104000CA | Q59QH4 | 0.18 | 2.81 | 0.06 |
| Bifunctional UDP-glucose 4-epimerase/aldose 1-epimerase | GAL10 | CAALFM_C102150WA | A0A1D8PCN0 | 0.25 | 4.02 | 0.06 |
| ATP-dependent RNA helicase | DHH1 | CaO19.13577 | Q5AAW3 | 0.17 | 2.81 | 0.06 |
| Guanosine-diphosphatase (GDPase) | GDA1 | CaO19.7394 | Q8TGH6 | 1.04 | 16.87 | 0.06 |
| Gst2p | GST2 | CAALFM_C402990CA | Q5AFB4 | 0.17 | 2.81 | 0.06 |
| Uncharacterized protein |  | CAALFM_C603320WA | A0A1D8PQ54 | 0.31 | 5.17 | 0.06 |
| Glycerol-3-phosphate dehydrogenase [NAD(+)] | GPD2 | CAALFM_C602010CA | Q59W33 | 0.25 | 4.22 | 0.06 |
| Uncharacterized protein |  | CAALFM_C303410CA | Q5AED0 | 0.50 | 8.43 | 0.06 |
| Oxidoreductase |  | CAALFM_C401510WA | Q5AML3 | 0.25 | 4.22 | 0.06 |
| Methionine--tRNA ligase | MES1 | CAALFM_C504700CA | A0A1D8PP21 | 0.60 | 10.35 | 0.06 |
| Translation initiation factor eIF4G | TIF4631 | CAALFM_C208760CA | A0A1D8PI73 | 0.10 | 1.72 | 0.06 |
| Ribosomal 60S subunit protein L34B |  | CAALFM_C106890CA | A0A1D8PDZ1 | 0.10 | 1.72 | 0.06 |
| T-complex protein 1 subunit delta |  | CAALFM_C402780WA | A0A1D8PLN3 | 0.10 | 1.72 | 0.06 |
| Cytochrome b-c1 complex subunit 2, mitochondrial | QCR2 | CaO19.10167 | P83782 | 1.54 | 28.11 | 0.05 |
| Arginine--tRNA ligase |  | CAALFM_C101530CA | A0A1D8PCI5 | 0.31 | 6.32 | 0.05 |
| Adenine phosphoribosyltransferase (APRT) | APT1 | CaO19.1448 | Q5ALX8 | 0.55 | 11.24 | 0.05 |
| Adenylosuccinate synthetase (AMPSase) (AdSS) | ADE12 | CaO19.12290 | P0CH96 | 0.96 | 19.68 | 0.05 |
| 40S ribosomal protein S7 | RPS7A | CAALFM_C301490WA | Q5AJ93 | 0.80 | 16.87 | 0.05 |
| Lys22p | LYS22 | CAALFM_C204460WA | Q59TC4 | 1.17 | 25.04 | 0.05 |
| Ali1p | ALI1 | CAALFM_C301410CA | A0A1D8PJ73 | 0.16 | 3.45 | 0.05 |
| Respiratory growth induced protein 1 | RGI1 | CaO19.1354 | Q59KG2 | 0.81 | 18.02 | 0.04 |
| V-type proton ATPase subunit |  | CAALFM_C400020WA | A0A1D8PKX3 | 0.08 | 1.72 | 0.04 |
| Pyrroline-5-carboxylate reductase | PRO3 | CAALFM_C400240CA | A0A1D8PKZ7 | 0.08 | 1.72 | 0.04 |
| Mitogen-activated protein kinase MKC1 | MKC1 | CaO19.7523 | Q5AAG6 | 0.10 | 2.24 | 0.04 |
| Ribosomal 40S subunit protein S19A | RPS19A | CAALFM_C305200WA | A0A1D8PK61 | 0.34 | 8.05 | 0.04 |
| Hsp70 family ATPase | SSB1 | CAALFM_CR08090WA | Q5A397 | 3.54 | 86.63 | 0.04 |
| Acetyl-CoA carboxylase | ACC1 | CAALFM_CR00640WA | A0A1D8PRR7 | 0.78 | 19.54 | 0.04 |
| Uncharacterized protein |  | CAALFM_CR09140CA | A0A1D8PTW2 | 0.17 | 4.60 | 0.04 |
| Heat shock protein 60, mitochondrial (60 kDa chaperonin) | HSP60 | CaO19.717 | O74261 | 0.25 | 7.03 | 0.04 |
| Candidapepsin-3 | SAP3 | CaO19.13422 | P0CY29 | 0.41 | 11.82 | 0.03 |
| Ribosomal 60S subunit protein L21A | RPL21A | CAALFM_C203810CA | A0A1D8PGY0 | 0.16 | 4.60 | 0.03 |
| Ribosomal 60S subunit protein L5 | RPL5 | CAALFM_C701790CA | Q5AGZ7 | 1.29 | 37.69 | 0.03 |
| Translation elongation factor 1 subunit beta | EFB1 | CAALFM_C404480CA | A0A1D8PM35 | 0.08 | 2.30 | 0.03 |
| Proteasome regulatory particle lid subunit | RPN7 | CAALFM_CR08910CA | A0A1D8PTV0 | 0.08 | 2.30 | 0.03 |
| Long-chain fatty acid-CoA ligase | FAA4 | CAALFM_CR10160WA | A0A1D8PU56 | 0.08 | 2.30 | 0.03 |
| Glycylpeptide N-tetradecanoyltransferase | NMT1 | CaO19.12111 | P30418 | 0.17 | 5.62 | 0.03 |
| Actin-related protein 3 | ARP3 | CAALFM_C207320WA | Q59Z11 | 0.17 | 5.62 | 0.03 |
| Pho112p | PHO112 | CAALFM_CR02400WA | A0A1D8PS71 | 0.38 | 13.22 | 0.03 |
| Succinate dehydrogenase [ubiquinone] flavoprotein subunit, mitochondrial | SDH12 | CAALFM_C406610CA | Q5A1E8 | 0.57 | 19.68 | 0.03 |
| 60S ribosomal protein L13 | RPL13 | Ca49C10.02 | O59931 | 0.15 | 5.62 | 0.03 |
| Ribosomal 40S subunit protein S10A | RPS10 | CAALFM_C208040CA | A0A1D8PI15 | 0.16 | 5.75 | 0.03 |
| Increased recombination centers protein 22-2 | IRC22-2 | CaO19.1782 | Q59XW9 | 0.08 | 2.81 | 0.03 |
| Coatomer subunit delta | RET2 | CAALFM_C502300CA | A0A1D8PND9 | 0.08 | 2.87 | 0.03 |
| Phosphoglycerate dehydrogenase | SER33 | CAALFM_C112030WA | Q5A3K7 | 0.70 | 26.71 | 0.03 |
| Threonine synthase | THR4 | CAALFM_C502270WA | A0A1D8PNG9 | 0.16 | 6.32 | 0.02 |
| Glutamine--fructose-6-phosphate aminotransferase [isomerizing] | GFA1 | CaO19.1618 | P53704 | 0.33 | 13.22 | 0.02 |
| Hgt7p | HGT7 | CAALFM_C201000WA | A0A1D8PG81 | 0.10 | 4.02 | 0.02 |
| Ribosomal 40S subunit protein S14B | RPS14B | CAALFM_C106450CA | A0A1D8PDT3 | 0.18 | 8.05 | 0.02 |
| Ribosomal 60S subunit protein L24A | RPL24A | CAALFM_C404890CA | Q5A6A1 | 0.16 | 7.03 | 0.02 |
| Uncharacterized protein |  | CAALFM_C111860WA | A0A1D8PF90 | 0.16 | 7.47 | 0.02 |
| Heat shock protein homolog SSE1 | MSI3 | CaO19.2435 | Q96VB9 | 0.56 | 26.77 | 0.02 |
| Ribosomal protein L19 | RPL19A | CAALFM_C304500CA | A0A1D8PK40 | 0.17 | 8.62 | 0.02 |
| 40S ribosomal protein S1 (S3aE) | RPS1 | CaO19.10520 | P40910 | 0.56 | 29.52 | 0.02 |
| V-type proton ATPase subunit C | VMA5 | CAALFM_C208190WA | Q5A2U9 | 0.08 | 4.22 | 0.02 |
| Glutamate--tRNA ligase | GUS1 | CAALFM_C700620WA | A0A1D8PQL8 | 0.16 | 8.62 | 0.02 |
| Chaperonin-containing T-complex subunit | CCT7 | CAALFM_C501690CA | Q59YH4 | 0.17 | 9.84 | 0.02 |
| Cytochrome c oxidase subunit 2 | COX2 | CaalfMp01 | Q9B8D8 | 0.10 | 5.62 | 0.02 |
| Eukaryotic translation initiation factor 3 subunit B (eIF3b) | PRT1 | CaO19.13937 | Q5AGV4 | 0.10 | 5.62 | 0.02 |
| Proline--tRNA ligase |  | CAALFM_C703660CA | Q59R20 | 0.41 | 23.89 | 0.02 |
| Valine--tRNA ligase | VAS1 | CAALFM_C206640CA | A0A1D8PHR0 | 0.16 | 9.20 | 0.02 |
| Proteasome regulatory particle base subunit | RPN2 | CAALFM_C112050WA | Q5A3L0 | 0.25 | 15.46 | 0.02 |
| Malate dehydrogenase, cytoplasmic | MDH1 | CaO19.7481 | P83778 | 0.42 | 26.71 | 0.02 |
| Thioredoxin reductase | TRR1 | CAALFM_C502710WA | Q5AG89 | 0.17 | 11.24 | 0.02 |
| Aspartate--tRNA ligase | DPS1-1 | CAALFM_CR03170WA | Q59UF7 | 0.23 | 15.46 | 0.02 |
| Dihydroxyacetone kinase | DAK2 | CAALFM_C109190CA | A0A1D8PEI2 | 0.08 | 5.17 | 0.01 |
| Type I HSP40 co-chaperone | YDJ1 | CAALFM_CR04200WA | A0A1D8PSQ3 | 0.08 | 5.75 | 0.01 |
| Serine hydroxymethyltransferase, cytosolic (SHMT) | SHM2 | CaO19.13173 | O13426 | 0.25 | 19.68 | 0.01 |
| Coatomer subunit beta (Beta-coat protein) | SEC26 | CAALFM_CR04380CA | Q5A6M6 | 0.08 | 6.20 | 0.01 |
| Threonine--tRNA ligase | THS1 | CAALFM_C500110CA | A0A1D8PMV9 | 0.08 | 6.32 | 0.01 |
| Ribosomal 40S subunit protein S11A |  | CAALFM_C501540WA | A0A1D8PN83 | 0.08 | 6.32 | 0.01 |
| Glycogen [starch] synthase | GSY1 | CAALFM_CR00780CA | Q5A850 | 0.10 | 8.43 | 0.01 |
| Type II HSP40 co-chaperone | SIS1 | CAALFM_CR06080WA | Q59V92 | 0.08 | 7.03 | 0.01 |
| T-complex protein 1 subunit gamma | CCT3 | CAALFM_C505120WA | Q5AK16 | 0.10 | 9.84 | 0.01 |
| Ribosomal 40S subunit protein S2 | RPS21 | CAALFM_C101480CA | Q5A900 | 0.34 | 35.14 | 0.01 |
| Heat shock protein SSC1, mitochondrial (Cytoplasmic antigenic protein 6) (mtHSP70) | SSC1 | CaO19.1896 | P83784 | 0.31 | 46.96 | 0.01 |
| Pho100p | PHO100 | CAALFM_C107430WA | Q59WE5 | 0.16 | 24.53 | 0.01 |
| V-type proton ATPase subunit B (V-ATPase subunit B) (Vacuolar proton pump subunit B) | VMA2 | CaO19.13955 | Q59PT0 | 0.16 | 26.71 | 0.01 |
| Ribosomal 60S subunit protein L16A | RPL16A | CAALFM_C100180WA | Q5AB87 | 0.10 | 16.87 | 0.01 |
| Bifunctional phosphoribosylaminoimidazolecarboxamide formyltransferase/IMP cyclohydrolase | ADE17 | CAALFM_CR04090CA | Q5A6R2 | 0.25 | 49.19 | 0.01 |
| Heat shock protein SSA2 | SSA2 | CaO19.1065 | P46587 | 0.00 | 93.60 | yeast unique |
| Ribosomal 60S subunit protein L3 | RPL3 | CAALFM_C209430WA | Q59LS1 | 0.16 | 61.01 | yeast unique |
| Ribosomal 40S subunit protein S5 | RPS5 | CAALFM_C503070WA | Q5AG43 | 0.16 | 44.40 | yeast unique |
| Malate dehydrogenase | MDH1-1 | CAALFM_C401900CA | Q5AMP4 | 0.17 | 35.71 | yeast unique |
| Aconitate hydratase, mitochondrial (Aconitase) | ACO1 | CaO19.13742 | P82611 | 0.00 | 35.46 | yeast unique |
| Ribosomal 60S subunit protein L8B | RPL8B | CAALFM_C305240CA | Q5ANA1 | 0.00 | 34.31 | yeast unique |
| Ribosomal 60S subunit protein L10 | RPL10 | CAALFM_C102460WA | Q5AIB8 | 0.16 | 32.90 | yeast unique |
| ATP-dependent 6-phosphofructokinase (ATP-PFK) (Phosphofructokinase) | PFK1 | CAALFM_C504810WA | Q5AK53 | 0.00 | 29.52 | yeast unique |
| V-type proton ATPase catalytic subunit A (V-ATPase subunit A) | TFP1 | CaO19.9249 | Q5AJB1 | 0.10 | 29.26 | yeast unique |
| Lysophospholipase 1 (EC 3.1.1.5) (CaPLB1) (Phospholipase B 1) | PLB1 | CaO19.689 | Q9UWF6 | 0.00 | 28.69 | yeast unique |
| 40S ribosomal protein S8 | RPS8A | CAALFM_C205610CA | Q59T44 | 0.00 | 26.71 | yeast unique |
| Pentafunctional AROM polypeptide [Includes: 3-dehydroquinate synthase (DHQS) | ARO1 | CaO19.12175 | Q5AME2 | 0.00 | 26.71 | yeast unique |
| Phosphoribosylformylglycinamidine synthase | ADE6 | CAALFM_CR04740CA | Q59MZ5 | 0.00 | 26.71 | yeast unique |
| Pyruvate dehydrogenase E1 component subunit alpha | PDA1 | CAALFM_C407110CA | Q5A0Z9 | 0.00 | 23.89 | yeast unique |
| Alanine--tRNA ligase | ALA1 | CaO19.13169 | Q5A8K2 | 0.00 | 22.23 | yeast unique |
| Ribosomal 60S subunit protein L4B | RPL4B | CAALFM_C114110CA | A0A1D8PFV1 | 0.00 | 21.84 | yeast unique |
| Bifunctional carbamoylphosphate synthetase/aspartate transcarbamylase | URA2 | CAALFM_CR07050CA | A0A1D8PTD1 | 0.00 | 21.26 | yeast unique |
| Trifunctional formate-tetrahydrofolate ligase/methenyltetrahydrofolate cyclohydrolase/methylenetetrahydrofolate dehydrogenase | MIS11 | CAALFM_CR07010WA | Q59SM8 | 0.00 | 21.08 | yeast unique |
| Pyruvate dehydrogenase E1 component subunit beta | PDB1 | CAALFM_C404150CA | Q5A5V6 | 0.00 | 20.25 | yeast unique |
| Lipid-binding protein | LSP1 | CAALFM_C206730WA | Q59KV8 | 0.08 | 19.68 | yeast unique |
| Cystathionine beta-synthase | CYS4 | CAALFM_C101870CA | Q59T95 | 0.00 | 18.27 | yeast unique |
| Eukaryotic translation initiation factor 3 subunit C (eIF3c) | NIP1 | CaO19.12105 | Q5AML1 | 0.00 | 18.27 | yeast unique |
| Homoisocitrate dehydrogenase | LYS12 | CAALFM_CR01400WA | Q5A9D9 | 0.00 | 17.70 | yeast unique |
| Ribosomal protein L15 | RPL15A | CAALFM_CR04100CA | Q5A6R1 | 0.00 | 17.44 | yeast unique |
| Lysine--tRNA ligase | KRS1 | CAALFM_C307410CA | Q5ADU2 | 0.00 | 15.46 | yeast unique |
| Chaperonin-containing T-complex subunit | CCT5 | CAALFM_C207310WA | Q59Z12 | 0.00 | 14.63 | yeast unique |
| Leucine--tRNA ligase | CDC60 | CAALFM_CR01690CA | A0A1D8PS12 | 0.00 | 14.37 | yeast unique |
| Uncharacterized protein |  | CAALFM_C204370WA | Q59TB4 | 0.00 | 14.06 | yeast unique |
| GMP synthase [glutamine-hydrolyzing] | GUA1 | CaO19.12276 | Q5APF2 | 0.00 | 14.06 | yeast unique |
| Elongation factor Tu | TUF1 | CAALFM_C100590WA | Q5ABC3 | 0.00 | 13.48 | yeast unique |
| Chaperonin-containing T-complex alpha subunit | TCP1 | CAALFM_C108560WA | Q59QB7 | 0.00 | 13.22 | yeast unique |
| Polyadenylate-binding protein, cytoplasmic and nuclear (PABP) | PAB1 | CaO19.10555 | Q5AI15 | 0.00 | 13.22 | yeast unique |
| Serine--tRNA ligase, cytoplasmic | SES1 | CaO19.7901 | Q9HGT6 | 0.00 | 13.22 | yeast unique |
| Glycine--tRNA ligase | GRS1 | CAALFM_C105290WA | Q5A2A5 | 0.00 | 12.65 | yeast unique |
| Ribonucleoside-diphosphate reductase | RNR1 | CAALFM_C203160CA | Q5A0N3 | 0.00 | 12.65 | yeast unique |
| ATP-binding cassette family ATPase | KRE30 | CAALFM_C208000CA | Q5A2T2 | 0.00 | 12.65 | yeast unique |
| Clustered mitochondria protein homolog (Protein TIF31 homolog) | CLU1 | CaO19.51 | Q59MA9 | 0.00 | 12.65 | yeast unique |
| Fe-S cluster-binding ribosome biosynthesis protein | RLI1 | CAALFM_C103350CA | Q5AI20 | 0.00 | 11.82 | yeast unique |
| AAA family ATPase | CDC48 | CAALFM_C110790WA | Q59WG3 | 0.00 | 11.82 | yeast unique |
| Ribosomal 60S subunit protein L2A | RPL2 | CAALFM_C111060CA | A0A1D8PF08 | 0.00 | 11.49 | yeast unique |
| Flavohemoprotein | YHB1 | CaO19.11192 | Q59MV9 | 0.00 | 11.24 | yeast unique |
| Inosine-5'-monophosphate dehydrogenase (IMP dehydrogenase) (IMPD) (IMPDH) | IMH3 | CaO19.18 | Q59Q46 | 0.00 | 11.24 | yeast unique |
| Cytochrome c peroxidase, mitochondrial (CCP) | CCP1 | CaO19.238 | Q5AEN1 | 0.00 | 11.24 | yeast unique |
| Trehalose-phosphatase | TPS2 | CAALFM_C103380WA | Q5AI14 | 0.00 | 10.99 | yeast unique |
| Ssz1p | SSZ1 | CAALFM_C404700WA | Q5A678 | 0.00 | 10.41 | yeast unique |
| Tom40p | TOM40 | CAALFM_C701970CA | Q5AH14 | 0.00 | 10.41 | yeast unique |
| Alpha-ketoglutarate dehydrogenase | KGD1 | CAALFM_C300880WA | A0A1D8PJ26 | 0.00 | 10.35 | yeast unique |
| Chaperonin-containing T-complex subunit | CCT8 | CAALFM_C100110WA | Q5AB74 | 0.00 | 9.84 | yeast unique |
| Tyrosine--tRNA ligase | TYS1 | CAALFM_C402980WA | Q5AFB3 | 0.00 | 9.84 | yeast unique |
| Histidine--tRNA ligase | HTS1 | CAALFM_C505490CA | Q5AJX0 | 0.00 | 9.84 | yeast unique |
| Small COPII coat GTPase | SAR1 | CaO19.10966 | Q59S78 | 0.00 | 9.84 | yeast unique |
| ATP-dependent RNA helicase | DED1 | CaO19.7392 | Q5A4E2 | 0.00 | 9.84 | yeast unique |
| Lanosterol 14-alpha demethylase | ERG11 | CaO19.922 | P10613 | 0.00 | 9.84 | yeast unique |
| 40S ribosomal protein S6 | RPS6A | CAALFM_C401270WA | A0A1D8PL99 | 0.00 | 9.77 | yeast unique |
| Protein channel | TOM70 | CAALFM_C702640WA | Q59LZ5 | 0.00 | 9.01 | yeast unique |
| Protein-arginine omega-N methyltransferase | HMT1 | CAALFM_C101030WA | Q5A943 | 0.00 | 8.43 | yeast unique |
| Translation initiation factor eIF5B | FUN12 | CAALFM_C108090CA | Q5A782 | 0.00 | 8.43 | yeast unique |
| NADH dehydrogenase [ubiquinone] flavoprotein 1, mitochondrial | NDH51 | CAALFM_C204550CA | Q59TD5 | 0.00 | 8.43 | yeast unique |
| Phosphoserine aminotransferase | SER1 | CAALFM_C206210CA | Q59P52 | 0.00 | 8.43 | yeast unique |
| Proteasome regulatory particle lid subunit |  | CAALFM_C208930WA | Q59SD0 | 0.00 | 8.43 | yeast unique |
| Translation termination factor GTPase eRF3 | SUP35 | CAALFM_C209720WA | Q59YE8 | 0.00 | 8.43 | yeast unique |
| 40S ribosomal protein S24 | RPS24 | CAALFM_C300090WA | Q5A7K0 | 0.00 | 8.43 | yeast unique |
| Zuotin | ZUO1 | CAALFM_C402870CA | Q5AF98 | 0.00 | 8.43 | yeast unique |
| Obg-like ATPase 1 | YBN5 | CAALFM_C104890WA | A0A1D8PDE8 | 0.00 | 8.05 | yeast unique |
| Translation initiation factor eIF2 subunit alpha | SUI2 | CAALFM_C106960WA | Q5AAU7 | 0.00 | 7.86 | yeast unique |
| Eukaryotic translation initiation factor 3 subunit A (eIF3a) | RPG1A | CaO19.13701 | Q59PL9 | 0.00 | 7.60 | yeast unique |
| Bifunctional hydroxyacyl-CoA dehydrogenase/enoyl-CoA hydratase | FOX2 | CAALFM_C300810CA | A0A1D8PJ13 | 0.00 | 7.47 | yeast unique |
| Ribosomal 40S subunit protein S13 | RPS13 | CAALFM_C600650CA | A0A1D8PPE0 | 0.00 | 7.47 | yeast unique |
| 40S ribosomal protein S26 | RPS26A | CAALFM_C201610CA | Q5ALV6 | 0.00 | 7.03 | yeast unique |
| Ribosomal 60S subunit protein L17B | RPL17B | CAALFM_C204600CA | Q59TE0 | 0.00 | 7.03 | yeast unique |
| Translation termination factor eRF1 | ERF1 | CAALFM_C205100CA | Q59ZH8 | 0.00 | 7.03 | yeast unique |
| CTP synthase | URA7 | CAALFM_C504570CA | Q5AK79 | 0.00 | 7.03 | yeast unique |
| C-22 sterol desaturase | ERG5 | CAALFM_C702840CA | G1UB11 | 0.00 | 7.03 | yeast unique |
| Nucleolar protein 58 | NOP58 | CaO19.1199 | Q59S06 | 0.00 | 7.03 | yeast unique |
| Sterol 24-C-methyltransferase | ERG6 | CaO19.1631 | O74198 | 0.00 | 7.03 | yeast unique |
| ATP-dependent RNA helicase | DBP5 | CaO19.1661 | Q5AJD0 | 0.00 | 7.03 | yeast unique |
| Ribosomal 60S subunit protein L26B |  | CAALFM_C102330CA | A0A1D8PCQ5 | 0.00 | 6.90 | yeast unique |
| Glutamate synthase (NADH) | GLT1 | CAALFM_C106550WA | A0A1D8PDU9 | 0.00 | 6.90 | yeast unique |
| Ribosomal 40S subunit protein S15 | RPS15 | CAALFM_C304670CA | A0A1D8PK22 | 0.00 | 6.90 | yeast unique |
| 60S ribosomal protein L20 | RPL20B | CAALFM_C401520CA | A0A1D8PLC9 | 0.00 | 6.90 | yeast unique |
| rRNA methyltransferase | NOP1 | CAALFM_C406720WA | Q5A0V9 | 0.00 | 6.45 | yeast unique |
| Lipid-binding protein | PIL1 | CAALFM_C104680WA | A0A1D8PDD1 | 0.00 | 6.32 | yeast unique |
| Ubiquinol--cytochrome-c reductase catalytic subunit | CYT1 | CAALFM_C204950CA | A0A1D8PHA3 | 0.00 | 6.32 | yeast unique |
| Adh5p | ADH5 | CAALFM_CR02070CA | A0A1D8PS45 | 0.00 | 6.32 | yeast unique |
| Chaperonin-containing T-complex subunit | CCT2 | CAALFM_C209520CA | Q59YC4 | 0.00 | 6.20 | yeast unique |
| Uncharacterized protein |  | CAALFM_C400860CA | Q5AME0 | 0.00 | 6.20 | yeast unique |
| Serine/threonine-protein phosphatase | GLC7 | CAALFM_CR07650WA | Q59N42 | 0.00 | 6.20 | yeast unique |
| Ribosomal 40S subunit protein S17B | RPS17B | CAALFM_C110870WA | A0A1D8PEY9 | 0.00 | 5.75 | yeast unique |
| Coatomer subunit alpha |  | CAALFM_C301720CA | A0A1D8PJB0 | 0.00 | 5.75 | yeast unique |
| Uncharacterized protein |  | CAALFM_C602560WA | A0A1D8PPX6 | 0.00 | 5.75 | yeast unique |
| snoRNP complex protein | SIK1 | CAALFM_CR09950CA | A0A1D8PU46 | 0.00 | 5.75 | yeast unique |
| Proteasome regulatory particle base subunit | RPT1 | CAALFM_C105240CA | Q5A2A0 | 0.00 | 5.62 | yeast unique |
| Malate dehydrogenase | MDH1-3 | CAALFM_C210480WA | Q5A5S6 | 0.00 | 5.62 | yeast unique |
| Signal recognition particle subunit SRP68 |  | CAALFM_C306970WA | Q5ADN7 | 0.00 | 5.62 | yeast unique |
| Fesur1p | FESUR1 | CAALFM_C307060WA | Q5ADP7 | 0.00 | 5.62 | yeast unique |
| Sterol-4-alpha-carboxylate 3-dehydrogenase (Decarboxylating) | ERG26 | CAALFM_C406270CA | Q5A1B0 | 0.00 | 5.62 | yeast unique |
| Uracil phosphoribosyltransferase | FUR1 | CAALFM_C503390CA | Q59QT3 | 0.00 | 5.62 | yeast unique |
| Cytochrome b-c1 complex subunit 7 | QCR7 | CAALFM_C603400CA | Q5ABS1 | 0.00 | 5.62 | yeast unique |
| Nuc2p | NUC2 | CAALFM_C701900WA | Q5AH07 | 0.00 | 5.62 | yeast unique |
| NADH-cytochrome b5 reductase 1 | CBR1 | CaO19.1801 | Q59P03 | 0.00 | 5.62 | yeast unique |
| Small heat shock protein 21 | HSP21 | CaO19.822 | Q5AHH4 | 0.00 | 5.62 | yeast unique |
| Heat shock protein 78, mitochondrial | HSP78 | CaO19.8501 | Q96UX5 | 0.00 | 5.62 | yeast unique |
| 60S ribosomal protein L27 | RPL27A | CAALFM_C112390CA | A0A1D8PFG4 | 0.00 | 5.17 | yeast unique |
| Sulfite reductase (NADPH) subunit beta | ECM17 | CAALFM_C206170CA | A0A1D8PHL8 | 0.00 | 5.17 | yeast unique |
| Ribosomal 60S subunit protein L23B | RPL23A | CAALFM_C602070CA | A0A1D8PPT5 | 0.00 | 5.17 | yeast unique |
| Agglutinin-like protein 2 (Adhesin 2) | ALS2 | CaO19.1097 | P0CU38 | 0.00 | 5.17 | yeast unique |
| Ribosomal 60S subunit protein L28 | RPL28 | CAALFM_CR03030CA | A0A1D8PSC5 | 0.00 | 5.17 | yeast unique |
| Ife2p | IFE2 | CAALFM_CR05340CA | A0A1D8PSZ0 | 0.00 | 5.17 | yeast unique |
| Uncharacterized protein |  | CAALFM_CR10140WA | A0A1D8PU51 | 0.00 | 5.17 | yeast unique |
| Uncharacterized protein |  | CAALFM_C111120CA | Q59WJ7 | 0.00 | 4.79 | yeast unique |
| Succinate dehydrogenase [ubiquinone] iron-sulfur subunit, mitochondrial | SDH2 | CAALFM_CR05180CA | Q59QN7 | 0.00 | 4.79 | yeast unique |
| Uncharacterized protein |  | CAALFM_C702100WA | A0A1D8PR11 | 0.00 | 4.60 | yeast unique |
| Fumarate reductase | OSM2 | CAALFM_C113670WA | Q5AKX2 | 0.00 | 4.22 | yeast unique |
| Bfr1p | BFR1 | CAALFM_C402270CA | Q5AMT7 | 0.00 | 4.22 | yeast unique |
| Dihydroorotate dehydrogenase (quinone), mitochondrial (DHOD) (DHODase) | URA9 | CaO19.12299 | Q874I4 | 0.00 | 4.22 | yeast unique |
| Sulfate adenylyltransferase | MET3 | CaO19.12492 | Q9Y872 | 0.00 | 4.22 | yeast unique |
| 13 kDa ribonucleoprotein-associated protein | SNU13 | CaO19.13306 | Q5ANL6 | 0.00 | 4.22 | yeast unique |
| Purine nucleoside permease | NUP | CaO19.13923 | Q5AGW8 | 0.00 | 4.22 | yeast unique |
| Squalene monooxygenase | ERG1 | CaO19.406 | Q92206 | 0.00 | 4.22 | yeast unique |
| Eukaryotic translation initiation factor 3 subunit G (eIF3g) | TIF35 | CaO19.7236 | Q59ZV5 | 0.00 | 4.22 | yeast unique |
| Vacuolar protein 8 | VAC8 | CaO19.745 | Q59MN0 | 0.00 | 4.22 | yeast unique |
| Homoserine kinase (HK) (HSK) | THR1 | CaO19.923 | Q92209 | 0.00 | 4.22 | yeast unique |
| Glycogenin glucosyltransferase |  | CAALFM_C101360CA | A0A1D8PCG4 | 0.00 | 4.02 | yeast unique |
| Uncharacterized protein |  | CAALFM_C301610WA | A0A1D8PJA6 | 0.00 | 4.02 | yeast unique |
| Tryptophan synthase | TRP5 | CAALFM_C406110CA | A0A1D8PMH6 | 0.00 | 4.02 | yeast unique |
| Phenylalanine--tRNA ligase subunit alpha | FRS2 | CAALFM_C102710WA | A0A1D8PCT4 | 0.00 | 3.45 | yeast unique |
| Rnr22p | RNR22 | CAALFM_C207570WA | A0A1D8PI17 | 0.00 | 3.45 | yeast unique |
| Cytochrome b-c1 complex subunit Rieske, mitochondrial | RIP1 | CAALFM_C304430WA | A0A1D8PJX3 | 0.00 | 3.45 | yeast unique |
| Ribosomal 60S subunit protein L35A | RPL35 | CAALFM_C304960WA | A0A1D8PK30 | 0.00 | 3.45 | yeast unique |
| Phenylalanine--tRNA ligase subunit beta | FRS1 | CAALFM_CR01760CA | A0A1D8PS16 | 0.00 | 3.45 | yeast unique |
| Uncharacterized protein |  | CAALFM_CR10200WA | A0A1D8PU57 | 0.00 | 3.45 | yeast unique |
| FACT complex subunit SPT16 | CDC68 | CaO19.10402 | Q5A1D5 | 0.00 | 3.39 | yeast unique |
| Acetolactate synthase | ILV2 | CAALFM_C302320WA | A0A1D8PJF9 | 0.00 | 2.87 | yeast unique |
| Proteasome regulatory particle lid subunit | RPN6 | CAALFM_C403790WA | A0A1D8PLW8 | 0.00 | 2.87 | yeast unique |
| Ribosomal 60S subunit protein L30 | RPL30 | CAALFM_C404900WA | A0A1D8PM75 | 0.00 | 2.87 | yeast unique |
| Oxidative DNA demethylase | OFD1 | CAALFM_C405460CA | A0A1D8PMB7 | 0.00 | 2.87 | yeast unique |
| Chaperonin-containing T-complex subunit | CCT6 | CAALFM_C406830CA | A0A1D8PMN9 | 0.00 | 2.87 | yeast unique |
| Ribosomal 40S subunit protein S25B | RPS25B | CAALFM_C503540CA | A0A1D8PNQ6 | 0.00 | 2.87 | yeast unique |
| Ribosomal 60S subunit protein L32 | RPL32 | CAALFM_C601700WA | A0A1D8PPN6 | 0.00 | 2.87 | yeast unique |
| ATP synthase subunit gamma | ATP3 | CAALFM_CR01310WA | A0A1D8PRY3 | 0.00 | 2.87 | yeast unique |
| Pho88p | PHO88 | CAALFM_CR09320CA | A0A1D8PTX9 | 0.00 | 2.87 | yeast unique |
| GTP-binding protein | DRG1 | CAALFM_C108100WA | Q5A779 | 0.00 | 2.81 | yeast unique |
| Tryptophan--tRNA ligase | WRS1 | CAALFM_C112380CA | Q5A3P4 | 0.00 | 2.81 | yeast unique |
| Cysteine--tRNA ligase |  | CAALFM_C113030CA | Q5AL46 | 0.00 | 2.81 | yeast unique |
| Pdx1p | PDX1 | CAALFM_C113830CA | Q5AKV6 | 0.00 | 2.81 | yeast unique |
| 4a-hydroxytetrahydrobiopterin dehydratase | PHHB | CAALFM_C200480CA | Q5ACY8 | 0.00 | 2.81 | yeast unique |
| Erg28p | ERG28 | CAALFM_C201090CA | Q5AD51 | 0.00 | 2.81 | yeast unique |
| Cytochrome c oxidase subunit 6A, mitochondrial (Cytochrome c oxidase polypeptide VIa) | COX13 | CAALFM_C201590WA | Q5ALV9 | 0.00 | 2.81 | yeast unique |
| Cytochrome c oxidase subunit IV | COX4 | CAALFM_C201620WA | Q5ALV5 | 0.00 | 2.81 | yeast unique |
| Dolichyl-diphosphooligosaccharide--protein glycosyltransferase subunit | STT3 | CAALFM_C201670CA | Q5ALU8 | 0.00 | 2.81 | yeast unique |
| Mitochondrial nucleoid protein |  | CAALFM_C207190CA | Q59Z25 | 0.00 | 2.81 | yeast unique |
| Mitochondrial import inner membrane translocase subunit TIM44 | TIM44 | CAALFM_C300250CA | Q5A7M2 | 0.00 | 2.81 | yeast unique |
| Glycerol-1-phosphatase | RHR2 | CAALFM_C300320WA | Q5A7M9 | 0.00 | 2.81 | yeast unique |
| Nuo2p | NUO2 | CAALFM_C302940CA | Q5AEI1 | 0.00 | 2.81 | yeast unique |
| NADH-ubiquinone reductase (H(+)-translocating) | NDE1 | CAALFM_C303420CA | Q5AEC9 | 0.00 | 2.81 | yeast unique |
| Phb1p | PHB1 | CAALFM_C303730CA | Q59SS4 | 0.00 | 2.81 | yeast unique |
| Ceramide very long chain fatty acid hydroxylase | SCS7 | CAALFM_C404590WA | Q5A668 | 0.00 | 2.81 | yeast unique |
| Gvp36p | GVP36 | CAALFM_C405550CA | Q5A473 | 0.00 | 2.81 | yeast unique |
| Uncharacterized protein |  | CAALFM_C502660CA | Q5AG96 | 0.00 | 2.81 | yeast unique |
| Ribosome assembly factor mrt4 | MRT4 | CAALFM_C602770WA | Q5AC00 | 0.00 | 2.81 | yeast unique |
| Very-long-chain (3R)-3-hydroxyacyl-CoA dehydratase |  | CAALFM_C703050WA | G1UB68 | 0.00 | 2.81 | yeast unique |
| RuvB-like helicase 2 | RVB2 | CaJ7.0208 | Q5AGZ9 | 0.00 | 2.81 | yeast unique |
| Dolichyl-phosphate-mannose--protein mannosyltransferase 1 | PMT1 | CaJ7.0330 | O74189 | 0.00 | 2.81 | yeast unique |
| FK506-binding protein 3 | FPR3 | CaO19.1030 | Q59VR3 | 0.00 | 2.81 | yeast unique |
| ATPase | GET3 | CaO19.10482 | P0CB54 | 0.00 | 2.81 | yeast unique |
| Eukaryotic translation initiation factor 3 subunit I (eIF3i) | TIF34 | CaO19.10484 | Q5AI86 | 0.00 | 2.81 | yeast unique |
| Histone H2A.2 | HTA2 | CaO19.1051 | Q59VP2 | 0.00 | 2.81 | yeast unique |
| Probable metalloprotease | ARX1 | CaO19.10533 | Q5AI37 | 0.00 | 2.81 | yeast unique |
| Signal peptidase complex catalytic subunit | SEC11 | CaO19.10769 | Q5A869 | 0.00 | 2.81 | yeast unique |
| GDP-mannose transporter (GMT) | VRG4 | CaO19.1232 | Q5A477 | 0.00 | 2.81 | yeast unique |
| Multiple drug resistance-associated protein-like transporter 1 | MLT1 | CaO19.12566 | Q5A762 | 0.00 | 2.81 | yeast unique |
| Increased recombination centers protein 22-1 | IRC22-1 | CaO19.1372 | Q59YF4 | 0.00 | 2.81 | yeast unique |
| ATP-dependent RNA helicase | DBP2 | CaO19.171 | Q59LU0 | 0.00 | 2.81 | yeast unique |
| Eukaryotic translation initiation factor 6 (eIF-6) | TIF6 | CaO19.1815 | Q59L13 | 0.00 | 2.81 | yeast unique |
| Oxysterol-binding protein-like protein OBPa | OBPA | CaO19.3198 | Q9UW25 | 0.00 | 2.81 | yeast unique |
| Protein transport protein SEC61 subunit alpha | SEC61 | CaO19.6176 | Q9P8E3 | 0.00 | 2.81 | yeast unique |
| Mitogen-activated protein kinase | HOG1 | CaO19.8514 | Q92207 | 0.00 | 2.81 | yeast unique |
| Uncharacterized protein |  | CAALFM_CR01350CA | Q5A9E7 | 0.00 | 2.81 | yeast unique |
| NADH-ubiquinone oxidoreductase |  | CAALFM_CR02620CA | Q5A222 | 0.00 | 2.81 | yeast unique |
| Fumarase | FUM11 | CAALFM_CR04530WA | Q5A6L1 | 0.00 | 2.81 | yeast unique |
| E2 SUMO-conjugating protein |  | CAALFM_CR08560CA | Q5A339 | 0.00 | 2.81 | yeast unique |
| L-aminoadipate-semialdehyde dehydrogenase | LYS2 | CAALFM_C102820WA | A0A1D8PCV7 | 0.00 | 2.30 | yeast unique |
| Dolichyl-phosphate beta-D-mannosyltransferase | DPM1 | CAALFM_C108010WA | A0A1D8PEA2 | 0.00 | 2.30 | yeast unique |
| Ribosomal 60S subunit protein L42A | RPL42 | CAALFM_C110390CA | A0A1D8PEV4 | 0.00 | 2.30 | yeast unique |
| Uncharacterized protein |  | CAALFM_C301140WA | A0A1D8PJ36 | 0.00 | 2.30 | yeast unique |
| Gtt11p | GTT11 | CAALFM_C303720WA | A0A1D8PJR2 | 0.00 | 2.30 | yeast unique |
| Phosphopantothenate--cysteine ligase |  | CAALFM_C305760WA | A0A1D8PKB0 | 0.00 | 2.30 | yeast unique |
| Acetolactate synthase regulatory subunit | ILV6 | CAALFM_C401370WA | A0A1D8PLB7 | 0.00 | 2.30 | yeast unique |
| NEDD8-activating enzyme E1 regulatory subunit |  | CAALFM_C501590WA | A0A1D8PN68 | 0.00 | 2.30 | yeast unique |
| Translation initiation factor eIF5 | TIF5 | CAALFM_C502490CA | A0A1D8PNF5 | 0.00 | 2.30 | yeast unique |
| Ribosomal 60S subunit protein L43A | RPL43A | CAALFM_C504590CA | A0A1D8PP14 | 0.00 | 2.30 | yeast unique |
| Glutamine--tRNA ligase | GLN4 | CAALFM_C700550CA | A0A1D8PQM9 | 0.00 | 2.30 | yeast unique |
| Imidazoleglycerol-phosphate synthase | HIS7 | CAALFM_C703720CA | A0A1D8PRE9 | 0.00 | 2.30 | yeast unique |
| Arc1p | ARC1 | CAALFM_CR03060WA | A0A1D8PSC8 | 0.00 | 2.30 | yeast unique |
| Aconitate hydratase, mitochondrial (Aconitase) | ACO2 | CAALFM_CR05790CA | A0A1D8PT27 | 0.00 | 2.30 | yeast unique |
| Protein SDS23 | SDS24 | Ca41C10.01c | Q5A744 | 0.00 | 1.98 | yeast unique |
| Respiratory supercomplex factor 1, mitochondrial | RCF1 | CaO19.1114 | Q59N74 | 0.00 | 1.98 | yeast unique |
| Ribosomal 40S subunit protein S20 | RPS20 | CAALFM_CR08150WA | Q5A389 | 0.00 | 1.98 | yeast unique |
| Ubiquitin-specific protease | UBP6 | CAALFM_C100440WA | A0A1D8PC77 | 0.00 | 1.72 | yeast unique |
| Uncharacterized protein |  | CAALFM_C102650WA | A0A1D8PCU5 | 0.00 | 1.72 | yeast unique |
| Uncharacterized protein |  | CAALFM_C107160CA | A0A1D8PE03 | 0.00 | 1.72 | yeast unique |
| Smi1p | SMI1 | CAALFM_C107870CA | A0A1D8PE53 | 0.00 | 1.72 | yeast unique |
| Phosphate transporter | PHO84 | CAALFM_C111480WA | A0A1D8PF54 | 0.00 | 1.72 | yeast unique |
| Ribosomal 60S subunit protein L33A |  | CAALFM_C205710CA | A0A1D8PHH4 | 0.00 | 1.72 | yeast unique |
| Gcn1p | GCN1 | CAALFM_C210550CA | A0A1D8PIR2 | 0.00 | 1.72 | yeast unique |
| Deoxyhypusine synthase |  | CAALFM_C302180CA | A0A1D8PJD2 | 0.00 | 1.72 | yeast unique |
| Aspartate aminotransferase | AAT22 | CAALFM_C401200CA | A0A1D8PL85 | 0.00 | 1.72 | yeast unique |
| MICOS complex subunit |  | CAALFM_C404970CA | A0A1D8PM81 | 0.00 | 1.72 | yeast unique |
| Aspartokinase | HOM3 | CAALFM_C405540WA | A0A1D8PMB8 | 0.00 | 1.72 | yeast unique |
| Hsp90 cochaperone | STI1 | CAALFM_C501820WA | A0A1D8PN90 | 0.00 | 1.72 | yeast unique |
| Ribosomal 60S subunit protein L25 | RPL25 | CAALFM_C601970CA | A0A1D8PPS1 | 0.00 | 1.72 | yeast unique |
| Aminomethyltransferase | GCV1 | CAALFM_C602500CA | A0A1D8PPW9 | 0.00 | 1.72 | yeast unique |
| Uncharacterized protein |  | CAALFM_C603380WA | A0A1D8PQ55 | 0.00 | 1.72 | yeast unique |
| Translation initiation factor eIF2 subunit beta | SUI3 | CAALFM_C704130CA | A0A1D8PRJ6 | 0.00 | 1.72 | yeast unique |
| 3-isopropylmalate dehydratase | LEU1 | CAALFM_CR00360CA | A0A1D8PRP8 | 0.00 | 1.72 | yeast unique |
| 40S ribosomal protein S30 | RPS30 | CAALFM_CR03770CA | A0A1D8PSK2 | 0.00 | 1.72 | yeast unique |
| Pyridoxamine-phosphate oxidase | PDX3 | CAALFM_CR04590CA | A0A1D8PSU2 | 0.00 | 1.72 | yeast unique |
| Glutamine synthetase | GLN1 | CAALFM_CR05050WA | A0A1D8PSY1 | 0.00 | 1.72 | yeast unique |
| Mitochondrial 2-oxodicarboxylate carrier |  | CAALFM_CR05480WA | A0A1D8PT04 | 0.00 | 1.72 | yeast unique |
| Uncharacterized protein |  | CAALFM_C100160CA | Q5AB84 | 0.00 | 1.41 | yeast unique |
| Zinc finger-containing protein | ZPR1 | CAALFM_C101110CA | Q5A934 | 0.00 | 1.41 | yeast unique |
| Uncharacterized protein |  | CAALFM_C104220CA | Q59VN7 | 0.00 | 1.41 | yeast unique |
| C-5 sterol desaturase | ERG3 | CAALFM_C104770CA | Q59VG6 | 0.00 | 1.41 | yeast unique |
| MICOS complex subunit |  | CAALFM_C105490CA | Q5A2C6 | 0.00 | 1.41 | yeast unique |
| Uncharacterized protein |  | CAALFM_C108050WA | Q5A785 | 0.00 | 1.41 | yeast unique |
| SUMO family protein | SMT3 | CAALFM_C111330CA | Q59W54 | 0.00 | 1.41 | yeast unique |
| F1F0 ATP synthase subunit g | ATP20 | CAALFM_C112590WA | Q59M30 | 0.00 | 1.41 | yeast unique |
| Uncharacterized protein |  | CAALFM_C200760CA | Q5AD19 | 0.00 | 1.41 | yeast unique |
| Glutamine-dependent NAD(+) synthetase |  | CAALFM_C201530CA | Q5ALW6 | 0.00 | 1.41 | yeast unique |
| Protein phosphatase PP2A regulatory subunit B | CDC55 | CAALFM_C201600CA | Q5ALV8 | 0.00 | 1.41 | yeast unique |
| Aspartate-semialdehyde dehydrogenase | HOM2 | CAALFM_C202370CA | Q5ALM0 | 0.00 | 1.41 | yeast unique |
| Proteasome regulatory particle base subunit | PR26 | CAALFM_C203060WA | Q5A0L8 | 0.00 | 1.41 | yeast unique |
| Uncharacterized protein |  | CAALFM_C205290CA | Q59ZG1 | 0.00 | 1.41 | yeast unique |
| 2-methoxy-6-polyprenyl-1,4-benzoquinol methylase, mitochondrial | COQ5 | CAALFM_C205470WA | Q59ZE2 | 0.00 | 1.41 | yeast unique |
| Uncharacterized protein |  | CAALFM_C205760CA | Q59T30 | 0.00 | 1.41 | yeast unique |
| Blm3p | BLM3 | CAALFM_C208010WA | Q5A2T3 | 0.00 | 1.41 | yeast unique |
| Glycerol-3-phosphate dehydrogenase [NAD(+)] | GPD1 | CAALFM_C210240WA | Q59XU9 | 0.00 | 1.41 | yeast unique |
| Hgt19p | HGT19 | CAALFM_C300220WA | Q5A7L9 | 0.00 | 1.41 | yeast unique |
| Arp9p | ARP9 | CAALFM_C301030WA | Q5A9X7 | 0.00 | 1.41 | yeast unique |
| Methylenetetrahydrofolate reductase | MET13 | CAALFM_C302950CA | Q5AEI0 | 0.00 | 1.41 | yeast unique |
| Ccc1p | CCC1 | CAALFM_C303710WA | Q59SS1 | 0.00 | 1.41 | yeast unique |
| Ptr22p | PTR22 | CAALFM_C303800WA | Q59ST2 | 0.00 | 1.41 | yeast unique |
| Uncharacterized protein |  | CAALFM_C305150WA | Q5ANB5 | 0.00 | 1.41 | yeast unique |
| Eukaryotic translation initiation factor 3 subunit H (eIF3h) |  | CAALFM_C307420WA | Q5ADU3 | 0.00 | 1.41 | yeast unique |
| F-actin-capping protein subunit beta |  | CAALFM_C401950WA | Q5AMP9 | 0.00 | 1.41 | yeast unique |
| Abp1p | ABP1 | CAALFM_C402940WA | Q5AFA8 | 0.00 | 1.41 | yeast unique |
| Vtc4p | VTC4 | CAALFM_C403360CA | Q59PH5 | 0.00 | 1.41 | yeast unique |
| Uncharacterized protein |  | CAALFM_C405810WA | Q5A441 | 0.00 | 1.41 | yeast unique |
| Translation machinery-associated protein 20 |  | CAALFM_C406170CA | Q5A199 | 0.00 | 1.41 | yeast unique |
| E2 ubiquitin-conjugating protein |  | CAALFM_C500560WA | Q5A513 | 0.00 | 1.41 | yeast unique |
| Erv46p | ERV46 | CAALFM_C500830CA | Q59X92 | 0.00 | 1.41 | yeast unique |
| 1-pyrroline-5-carboxylate dehydrogenase | PUT2 | CAALFM_C504880CA | Q5AK46 | 0.00 | 1.41 | yeast unique |
| Apm1p | APM1 | CAALFM_C505400WA | Q5AJY4 | 0.00 | 1.41 | yeast unique |
| Amino acid transporter | GNP1 | CAALFM_C600330CA | Q59RL1 | 0.00 | 1.41 | yeast unique |
| Yhm2p | YHM2 | CAALFM_C600600CA | Q59NH3 | 0.00 | 1.41 | yeast unique |
| Uncharacterized protein |  | CAALFM_C601420CA | Q5A4J9 | 0.00 | 1.41 | yeast unique |
| Uncharacterized protein |  | CAALFM_C601560WA | Q5A4L1 | 0.00 | 1.41 | yeast unique |
| Malate dehydrogenase (Oxaloacetate-decarboxylating) | MAE1 | CAALFM_C601670WA | Q5A4M2 | 0.00 | 1.41 | yeast unique |
| NADP-dependent alcohol dehydrogenase |  | CAALFM_C602480WA | Q5AC33 | 0.00 | 1.41 | yeast unique |
| Sphingolipid homeostasis protein | ORM1 | CAALFM_C603770CA | Q5A8J7 | 0.00 | 1.41 | yeast unique |
| Uncharacterized protein |  | CAALFM_C603820CA | Q5A8J1 | 0.00 | 1.41 | yeast unique |
| Membrane insertase |  | CAALFM_C701600WA | Q5AGX4 | 0.00 | 1.41 | yeast unique |
| NADH-ubiquinone oxidoreductase chain 3 | NAD3 | CaalfMp10 | Q9B8D1 | 0.00 | 1.41 | yeast unique |
| V-type proton ATPase subunit D (V-ATPase subunit D) (Vacuolar proton pump subunit D) | VMA8 | CaO19.10413 | P87220 | 0.00 | 1.41 | yeast unique |
| Translocation protein | SEC62 | CaO19.10549 | Q5AI21 | 0.00 | 1.41 | yeast unique |
| RuvB-like helicase 1 | RVB1 | CaO19.10641 | Q5A0W7 | 0.00 | 1.41 | yeast unique |
| Altered inheritance of mitochondria protein 9, mitochondrial | AIM9 | CaO19.10822 | Q5A922 | 0.00 | 1.41 | yeast unique |
| NAD(P)H-hydrate epimerase |  | CaO19.11163 | Q59VX9 | 0.00 | 1.41 | yeast unique |
| Virulence protein SSD1 | SSD1 | CaO19.11441 | Q5AK62 | 0.00 | 1.41 | yeast unique |
| Pescadillo homolog (Nucleolar protein 7 homolog) | NOP7 | CaO19.11574 | Q59X38 | 0.00 | 1.41 | yeast unique |
| MICOS complex subunit MIC60 (Mitofilin) | MIC60 | CaO19.11874 | Q5A044 | 0.00 | 1.41 | yeast unique |
| Elongation factor G, mitochondrial (EF-Gmt | MEF1 | CaO19.12398 | Q5AL45 | 0.00 | 1.41 | yeast unique |
| CAP1-binding-protein | YBP1 | CaO19.12501 | Q5AKU3 | 0.00 | 1.41 | yeast unique |
| Dolichyl-phosphate-mannose--protein mannosyltransferase 2 | PMT2 | CaO19.14104 | Q5ADM9 | 0.00 | 1.41 | yeast unique |
| Transcription elongation factor SPT5 (Chromatin elongation factor SPT5) | SPT5 | CaO19.1453 | Q5ALX3 | 0.00 | 1.41 | yeast unique |
| Actin cytoskeleton-regulatory complex protein SLA1 | SLA1 | CaO19.1474 | Q5ALV2 | 0.00 | 1.41 | yeast unique |
| Centromere/microtubule-binding protein | CBF5 | CaO19.1833 | O43101 | 0.00 | 1.41 | yeast unique |
| Catabolic 3-dehydroquinase (cDHQase) | DQD1 | CaO19.2283 | Q59Z17 | 0.00 | 1.41 | yeast unique |
| Deoxyhypusine hydroxylase (DOHH) | LIA1 | CaO19.2286 | Q59Z14 | 0.00 | 1.41 | yeast unique |
| Presequence translocated-associated motor subunit PAM17, mitochondrial | PAM17 | CaO19.240 | Q5AEM8 | 0.00 | 1.41 | yeast unique |
| Mitochondrial import inner membrane translocase subunit TIM50 | TIM50 | CaO19.680 | Q59W44 | 0.00 | 1.41 | yeast unique |
| Homoserine O-acetyltransferase | MET2 | CAALFM_CR02170WA | Q5A948 | 0.00 | 1.41 | yeast unique |
| Proteasome regulatory particle base subunit | RPT4 | CAALFM_CR04000WA | Q5A6S2 | 0.00 | 1.41 | yeast unique |
| Phenylpyruvate decarboxylase | ARO10 | CAALFM_CR06860CA | Q59MU3 | 0.00 | 1.41 | yeast unique |
| Type 2C protein phosphatase | PTC5 | CAALFM_CR08160WA | Q5A388 | 0.00 | 1.41 | yeast unique |
| Mitochondrial pyruvate carrier |  | CAALFM_CR08610WA | Q5A328 | 0.00 | 1.41 | yeast unique |
| Proteasome regulatory particle lid subunit | RPN3 | CAALFM_C103520WA | A0A1D8PD15 | 0.00 | 1.15 | yeast unique |
| 3-hydroxy-3-methylglutaryl coenzyme A reductase (HMG-CoA reductase) | HMG1 | CAALFM_C103780CA | A0A1D8PD39 | 0.00 | 1.15 | yeast unique |
| Cyclin-dependent serine/threonine-protein kinase | PHO85 | CAALFM_C104520CA | A0A1D8PDA6 | 0.00 | 1.15 | yeast unique |
| Ssh1p | SSH1 | CAALFM_C105530CA | A0A1D8PDL0 | 0.00 | 1.15 | yeast unique |
| Oxysterol-binding protein related protein |  | CAALFM_C108180CA | A0A1D8PE79 | 0.00 | 1.15 | yeast unique |
| Glycine cleavage system P protein | GCV2 | CAALFM_C108400CA | A0A1D8PE97 | 0.00 | 1.15 | yeast unique |
| Uncharacterized protein |  | CAALFM_C109020WA | A0A1D8PEH4 | 0.00 | 1.15 | yeast unique |
| Uncharacterized protein |  | CAALFM_C111670WA | A0A1D8PF63 | 0.00 | 1.15 | yeast unique |
| Ribosomal 60S subunit protein L38 | RPL38 | CAALFM_C200210WA | A0A1D8PG16 | 0.00 | 1.15 | yeast unique |
| Uncharacterized protein |  | CAALFM_C202170WA | A0A1D8PGJ8 | 0.00 | 1.15 | yeast unique |
| Uncharacterized protein |  | CAALFM_C202310WA | A0A1D8PGJ9 | 0.00 | 1.15 | yeast unique |
| Ribosomal 60S subunit protein L31B |  | CAALFM_C205410WA | A0A1D8PHF5 | 0.00 | 1.15 | yeast unique |
| Uncharacterized protein |  | CAALFM_C207010WA | A0A1D8PHU4 | 0.00 | 1.15 | yeast unique |
| Proteasome regulatory particle base subunit | RPT2 | CAALFM_C300290WA | A0A1D8PIV7 | 0.00 | 1.15 | yeast unique |
| Met18p (Fragment) | MET18 | CAALFM_C301450CA | A0A1D8PJ95 | 0.00 | 1.15 | yeast unique |
| GTPase-activating protein | RNA1 | CAALFM_C301990WA | A0A1D8PJD7 | 0.00 | 1.15 | yeast unique |
| D-lactate dehydrogenase | AIP2 | CAALFM_C303040WA | A0A1D8PJK5 | 0.00 | 1.15 | yeast unique |
| Dolichyl-diphosphooligosaccharide--protein glycosyltransferase subunit 1 | OST1 | CAALFM_C305530WA | A0A1D8PK87 | 0.00 | 1.15 | yeast unique |
| Methylenetetrahydrofolate dehydrogenase (NAD(+)) |  | CAALFM_C404720WA | A0A1D8PM44 | 0.00 | 1.15 | yeast unique |
| Coatomer subunit epsilon |  | CAALFM_C500080CA | A0A1D8PMT6 | 0.00 | 1.15 | yeast unique |
| Uncharacterized protein |  | CAALFM_C500100CA | A0A1D8PMU2 | 0.00 | 1.15 | yeast unique |
| Dynamin-like GTPase | VPS1 | CAALFM_C501210WA | A0A1D8PN45 | 0.00 | 1.15 | yeast unique |
| Ribulose-phosphate 3-epimerase |  | CAALFM_C700150WA | A0A1D8PQI4 | 0.00 | 1.15 | yeast unique |
| Replication factor C subunit 2 | RFC2 | CAALFM_C700820WA | A0A1D8PQP2 | 0.00 | 1.15 | yeast unique |
| Blood-induced peptide 1 | BLP1 | CaO19.4914.1 | P0CT51 | 0.00 | 1.15 | yeast unique |
| Uncharacterized protein |  | CAALFM_CR00310CA | A0A1D8PRQ2 | 0.00 | 1.15 | yeast unique |
| Anthranilate synthase | TRP2 | CAALFM_CR01590CA | A0A1D8PS03 | 0.00 | 1.15 | yeast unique |
| Uncharacterized protein |  | CAALFM_CR03120WA | A0A1D8PSE0 | 0.00 | 1.15 | yeast unique |
| Alpha-ketoglutarate dehydrogenase | KGD2 | CAALFM_CR07420WA | A0A1D8PTH3 | 0.00 | 1.15 | yeast unique |
| Uncharacterized protein |  | CAALFM_CR09330CA | A0A1D8PTZ1 | 0.00 | 1.15 | yeast unique |
| Cytochrome c lysine N-methyltransferase | CTM1 | CAALFM_CR10360CA | A0A1D8PU71 | 0.00 | 1.15 | yeast unique |
| Kis1p | KIS1 | CAALFM_C209230CA | Q59X47 | 0.00 | 0.83 | yeast unique |
| Uncharacterized protein |  | CAALFM_C100210CA | A0A1D8PC56 | 0.00 | 0.57 | yeast unique |
| Uncharacterized protein |  | CAALFM_C101010WA | A0A1D8PCD3 | 0.00 | 0.57 | yeast unique |
| Septin | CDC12 | CAALFM_C103210CA | A0A1D8PCY5 | 0.00 | 0.57 | yeast unique |
| Uncharacterized protein |  | CAALFM_C103310WA | A0A1D8PCZ5 | 0.00 | 0.57 | yeast unique |
| Succinate dehydrogenase [ubiquinone] cytochrome b small subunit |  | CAALFM_C103940WA | A0A1D8PD61 | 0.00 | 0.57 | yeast unique |
| Rgd1p | RGD1 | CAALFM_C106020WA | A0A1D8PDP3 | 0.00 | 0.57 | yeast unique |
| Ist2p | IST2 | CAALFM_C107520CA | A0A1D8PE41 | 0.00 | 0.57 | yeast unique |
| Amidophosphoribosyltransferase (ATase) | ADE4 | CAALFM_C107710CA | A0A1D8PE37 | 0.00 | 0.57 | yeast unique |
| Uncharacterized protein |  | CAALFM_C108080CA | A0A1D8PE69 | 0.00 | 0.57 | yeast unique |
| Mevalonate kinase (MK) | ERG12 | CAALFM_C109460WA | A0A1D8PEL1 | 0.00 | 0.57 | yeast unique |
| Erp5p | ERP5 | CAALFM_C110940CA | A0A1D8PF48 | 0.00 | 0.57 | yeast unique |
| Ribosomal protein L37 | RPL37B | CAALFM_C111360WA | A0A1D8PF45 | 0.00 | 0.57 | yeast unique |
| Bifunctional anthranilate synthase/indole-3-glycerol-phosphate synthase | TRP3 | CAALFM_C112210WA | A0A1D8PFC8 | 0.00 | 0.57 | yeast unique |
| Nicotinamide-nucleotide adenylyltransferase |  | CAALFM_C113270WA | Q5AL24 | 0.00 | 0.57 | yeast unique |
| SNAP receptor |  | CAALFM_C113690CA | A0A1D8PFS0 | 0.00 | 0.57 | yeast unique |
| Myosin 2 | MYO2 | CAALFM_C113780WA | Q5AKW2 | 0.00 | 0.57 | yeast unique |
| Rab family GTPase | YPT52 | CAALFM_C114100WA | A0A1D8PFT9 | 0.00 | 0.57 | yeast unique |
| Proteasome regulatory particle lid subunit |  | CAALFM_C114460WA | A0A1D8PFZ8 | 0.00 | 0.57 | yeast unique |
| Polyamine acetyltransferase |  | CAALFM_C114500CA | A0A1D8PFY0 | 0.00 | 0.57 | yeast unique |
| Uncharacterized protein |  | CAALFM_C200360CA | A0A1D8PG26 | 0.00 | 0.57 | yeast unique |
| Folylpolyglutamate synthase |  | CAALFM_C204380CA | A0A1D8PH56 | 0.00 | 0.57 | yeast unique |
| Ubiquinol--cytochrome-c reductase subunit 8 | QCR8 | CAALFM_C204590CA | A0A1D8PHA2 | 0.00 | 0.57 | yeast unique |
| Divalent metal ion transporter | SMF12 | CAALFM_C207160WA | A0A1D8PHV2 | 0.00 | 0.57 | yeast unique |
| Proteasome regulatory particle base subunit | RPT6 | CAALFM_C208780WA | A0A1D8PI93 | 0.00 | 0.57 | yeast unique |
| Uncharacterized protein |  | CAALFM_C209050CA | A0A1D8PI97 | 0.00 | 0.57 | yeast unique |
| Uncharacterized protein |  | CAALFM_C209910CA | A0A1D8PIL6 | 0.00 | 0.57 | yeast unique |
| Delta(24(24(1)))-sterol reductase | ERG4 | CAALFM_C300760WA | A0A1D8PJ25 | 0.00 | 0.57 | yeast unique |
| Arf family guanine nucleotide exchange factor | GEA2 | CAALFM_C301390CA | A0A1D8PJ74 | 0.00 | 0.57 | yeast unique |
| Cullin | CDC53 | CAALFM_C301700WA | Q5AJB7 | 0.00 | 0.57 | yeast unique |
| Medium-chain fatty acid-CoA ligase | FAA21 | CAALFM_C302810CA | A0A1D8PJI7 | 0.00 | 0.57 | yeast unique |
| Alanine transaminase | ALT1 | CAALFM_C303480CA | A0A1D8PJP4 | 0.00 | 0.57 | yeast unique |
| Sphinganine-1-phosphate aldolase |  | CAALFM_C303680WA | A0A1D8PJQ9 | 0.00 | 0.57 | yeast unique |
| Ctp1p | CTP1 | CAALFM_C304270CA | A0A1D8PJV4 | 0.00 | 0.57 | yeast unique |
| Dynamin-related GTPase | DNM1 | CAALFM_C305520CA | A0A1D8PKC9 | 0.00 | 0.57 | yeast unique |
| Uncharacterized protein |  | CAALFM_C400420CA | A0A1D8PL12 | 0.00 | 0.57 | yeast unique |
| Uncharacterized protein |  | CAALFM_C403290WA | A0A1D8PLT5 | 0.00 | 0.57 | yeast unique |
| Uncharacterized protein |  | CAALFM_C403370CA | A0A1D8PLT3 | 0.00 | 0.57 | yeast unique |
| Proteasome regulatory particle base subunit | RPT5 | CAALFM_C406870WA | A0A1D8PMQ4 | 0.00 | 0.57 | yeast unique |
| DNA-directed RNA polymerase subunit |  | CAALFM_C407060WA | A0A1D8PMS1 | 0.00 | 0.57 | yeast unique |
| ATP-dependent RNA helicase | NAM7 | CAALFM_C500500WA | Q5A507 | 0.00 | 0.57 | yeast unique |
| Sam51p | SAM51 | CAALFM_C500640CA | A0A1D8PN01 | 0.00 | 0.57 | yeast unique |
| Gpr1p | GPR1 | CAALFM_C501250WA | A0A1D8PN42 | 0.00 | 0.57 | yeast unique |
| 5-demethoxyubiquinone hydroxylase, mitochondrial (DMQ hydroxylase) | COQ7 | CAALFM_C501290CA | A0A1D8PN46 | 0.00 | 0.57 | yeast unique |
| Proteasome regulatory particle lid subunit | RPN8 | CAALFM_C502030WA | A0A1D8PNA8 | 0.00 | 0.57 | yeast unique |
| Vid27p | VID27 | CAALFM_C600130CA | A0A1D8PP94 | 0.00 | 0.57 | yeast unique |
| Yhm1p | YHM1 | CAALFM_C601930WA | A0A1D8PPR4 | 0.00 | 0.57 | yeast unique |
| Mitochondrial 37S ribosomal protein YmS18 |  | CAALFM_C601980CA | A0A1D8PPR6 | 0.00 | 0.57 | yeast unique |
| Mitochondrial 37S ribosomal protein RSM23 |  | CAALFM_C602370CA | A0A1D8PPW1 | 0.00 | 0.57 | yeast unique |
| Guanine nucleotide exchange factor |  | CAALFM_C602630CA | A0A1D8PPY0 | 0.00 | 0.57 | yeast unique |
| CCR4-NOT core subunit | CDC39 | CAALFM_C604270WA | A0A1D8PQD2 | 0.00 | 0.57 | yeast unique |
| Uncharacterized protein |  | CAALFM_C604290WA | A0A1D8PQC8 | 0.00 | 0.57 | yeast unique |
| Pex11p | PEX11 | CAALFM_C604310WA | A0A1D8PQD7 | 0.00 | 0.57 | yeast unique |
| Uncharacterized protein |  | CAALFM_C604560WA | A0A1D8PQF9 | 0.00 | 0.57 | yeast unique |
| Calcium-transporting ATPase | PMR1 | CAALFM_C700320CA | A0A1D8PQK6 | 0.00 | 0.57 | yeast unique |
| Uncharacterized protein |  | CAALFM_C700350CA | A0A1D8PQJ8 | 0.00 | 0.57 | yeast unique |
| 3-isopropylmalate dehydrogenase | LEU2 | CAALFM_C700400WA | A0A1D8PQK5 | 0.00 | 0.57 | yeast unique |
| Ribosomal 40S subunit protein S28B | RPS28B | CAALFM_C700710WA | A0A1D8PQN0 | 0.00 | 0.57 | yeast unique |
| Uncharacterized protein |  | CAALFM_C701160CA | A0A1D8PQU3 | 0.00 | 0.57 | yeast unique |
| Coronin | CRN1 | CAALFM_C701850CA | A0A1D8PQZ0 | 0.00 | 0.57 | yeast unique |
| Mam33p | MAM33 | CAALFM_C703930CA | A0A1D8PRG4 | 0.00 | 0.57 | yeast unique |
| Uncharacterized protein |  | CAALFM_C704210CA | A0A1D8PRJ1 | 0.00 | 0.57 | yeast unique |
| GDP-Man:Man(3)GlcNAc(2)-PP-Dol alpha-1,2-mannosyltransferase | ALG11 | CaO19.10972 | Q59S72 | 0.00 | 0.57 | yeast unique |
| Uncharacterized protein |  | CAALFM_CR01130WA | Q5A887 | 0.00 | 0.57 | yeast unique |
| Protein transport protein Sec61 subunit beta |  | CAALFM_CR01490CA | A0A1D8PRY9 | 0.00 | 0.57 | yeast unique |
| Mci4p | MCI4 | CAALFM_CR01740WA | Q5A995 | 0.00 | 0.57 | yeast unique |
| Vacuolar transporter chaperone | VTC3 | CAALFM_CR03610CA | A0A1D8PSJ6 | 0.00 | 0.57 | yeast unique |
| Protein-transporting protein |  | CAALFM_CR04080CA | A0A1D8PSM6 | 0.00 | 0.57 | yeast unique |
| RNA cytidine acetyltransferase | NAT10 | CAALFM_CR04240CA | Q5A6P2 | 0.00 | 0.57 | yeast unique |
| Uncharacterized protein |  | CAALFM_CR04730WA | A0A1D8PST6 | 0.00 | 0.57 | yeast unique |
| Rgd3p | RGD3 | CAALFM_CR07560WA | A0A1D8PTI9 | 0.00 | 0.57 | yeast unique |
| Actin-related protein 2 | ARP2 | CAALFM_CR08950WA | A0A1D8PTX1 | 0.00 | 0.57 | yeast unique |
| Nuo1p | NUO1 | CAALFM_CR09550CA | A0A1D8PU10 | 0.00 | 0.57 | yeast unique |

^1^Parameters used: minimum 95% peptide threshold, minimum 95% protein threshold and minimum of 2 peptides
