# Supplementary material for: Candida albicans biofilm–induced vesicles confer drug resistance through matrix biogenesis
Source: PLoS Biol. 2018 Oct 8;16(10):e2006872. doi: 10.1371/journal.pbio.2006872 (PMC6209495; doi:10.1371/journal.pbio.2006872)
Supplement: S3 Table — (DOCX) [file pbio.2006872.s004.docx]

**S3 Table. 1D ^1^H NMR chemical shift assignment of the major spin systems found in *C. albicans* extracellular matrix and extracellular vesicles.**

| Determination of percentages of the different mannose residues found in *C. albicans* biofilm extracellular vesicles and extracellular matrix. | | | |
| --- | --- | --- | --- |
| No. | **Residue type** | **Matrix** | **Vesicles** |
| A | α-1-2-Manα-1-3- | 3.7 | 25.8 |
| B | α-1-2-Manα-1-2- | 4.3 | 4.0 |
| C | α-1-2-Manα-1-2- | 7.2 | 3.2 |
| D | α-1-2-Manα-1-2- | 7.4 | 6.7 |
| E | β-1-2-Manα-1-2- | 11.9 | 6.5 |
| H | 2,6-Manα-1-6- (b) | 15.6 | 6.7 |
| I | Manα-1-2- | 13.5 | 5.1 |
| J | 3-Manα-1-2- | 7.2 | 23.8 |
| K | Manα-1-6- | 10.6 | 5.8 |
| L | 6-Manα-1-6- | 6.4 | 2.1 |
| M | β-1-2-Manβ-1-2- | 5.4 | 3.7 |
| N | Manβ-1-2- | 6.7 | 6.6 |
