# Supplementary material for: Candida albicans biofilm–induced vesicles confer drug resistance through matrix biogenesis
Source: PLoS Biol. 2018 Oct 8;16(10):e2006872. doi: 10.1371/journal.pbio.2006872 (PMC6209495; doi:10.1371/journal.pbio.2006872)
Supplement: S4 Table — (DOCX) [file pbio.2006872.s005.docx]

**S4 Table. Chemical shift assignment of the major spin systems found in *C. albicans* extracellular vesicles.**

| Chemical shift assignments of the major spin systems found in *C. albians* biofilm extracellular vesicles | | | | | | | | |
| --- | --- | --- | --- | --- | --- | --- | --- | --- |
| No. | **Residue type** | **Chemical shift (ppm)** | | | | | | |
|  |  | **1** | **2** | **3** | **4** | **5** | **6** | **6'** |
| A | α-1-2-Manα-1-3- | 5.37 | 4.10 | 3.99 | 3.70 | 3.77 | 3.90 | 3.77 |
|  |  | *103.4* | *81.4* | *73.1* | *70.1* | *76.2* | *64.1* |  |
| B | α-1-2-Manα-1-2- | 5.28 | 4.11 | 3.95 | 3.69 | 3.78 | 3.88 | 3.76 |
|  |  | *103.4* | *81.4* | *73.1* | *70.2* | *76.2* | *63.9* |  |
| C | α-1-2-Manα-1-2- | 5.27 | 4.12 | 3.95 | 3.73 | 3.78 | 3.88 | 3.76 |
|  |  | *103.3* | *81.2* | *72.9* | *69.2* | *76.2* | *63.9* |  |
| D | α-1-2-Manα-1-2- | 5.25 | 4.11 | 3.91 | 3.72 | 3.78 | 3.88 | 3.76 |
|  |  | *103.4* | *81.4* | *73.1* | *70.2* | *76.2* | *63.9* |  |
| E | β-1-2-Manα-1-2- | 5.15 | 4.26 | 3.90 | 3.73 | 3.78 | 3.88 | 3.76 |
|  |  | *102.8* | *81.2* | *73.1* | *70.2* | *76.2* | *63.9* |  |
| H | 2,6-Manα-1-6-(l) | 5.09 | 4.01 | 3.95 | 3.73 | 3.81 | 3.97 | 3.73 |
|  |  | *101.0* | *81.5* | *73.2* | *70.2* | *73.9* | *68.7* |  |
| I | Manα-1-2- | 5.05 | 4.07 | 3.84 | 3.67 | 3.76 | 3.88 | 3.76 |
|  |  | *104.9* | *73.2* | *73.5* | *69.9* | *76.2* | *63.9* |  |
| J | 3-Manα-1-2- | 5.04 | 4.21 | 3.93 | 3.78 | 3.76 | 3.86 | 3.77 |
|  |  | *104.8* | *72.6* | *81.2* | *69.2* | *76.3* | *64.1* |  |
| K | Manα-1-6- | 4.92 | 4.01 | 3.82 | 3.67 | 3.81 | 3.88 | 3.73 |
|  |  | *102.4* | *73.1* | *73.9* | *69.7* | *75.8* | *63.9* |  |
| L | 6-Manα-1-6- | 4.91 | 4.01 | 3.82 | 3.73 | 3.81 | 3.97 | 3.74 |
|  |  | *102.4* | *73.1* | *73.9* | *69.2* | *75.8* | *68.3* |  |
| M | β-1-2-Manβ-1-2- | 4.85 | 4.26 | 3.66 | 3.61 | 3.40 | 3.92 | 3.78 |
|  |  | *101.8* | *81.0* | *75.2* | *70.0* | *79.2* | *64.3* |  |
| N | Manβ-1-2- | 4.84 | 4.16 | 3.62 | 3.60 | 3.36 | 3.92 | 3.75 |
|  |  | *103.8* | *73.3* | *75.9* | *70.0* | *79.2* | *64.3* |  |
| O | β-1-6-Glcβ-1-6 | 4.52 | 3.34 | 3.49 | 3.45 | 3.63 | 4.22 | 3.86 |
|  |  | *105.7* | *76.0* | *78.7* | *72.5* | *77.7* | *71.6* |  |
