# Supplementary material for: Candida albicans biofilm–induced vesicles confer drug resistance through matrix biogenesis
Source: PLoS Biol. 2018 Oct 8;16(10):e2006872. doi: 10.1371/journal.pbio.2006872 (PMC6209495; doi:10.1371/journal.pbio.2006872)
Supplement: S5 Table — (DOCX) [file pbio.2006872.s006.docx]

**S5 Table. Primer sequences for strain construction**

| **Target Gene Primer Name** | **Function^1^** | **Primer Sequence** |
| --- | --- | --- |
| BRO1 F1 | K/O | TTCTTTGGTTTTCTTTCTGAAT |
| BRO1 F3 | K/O | gtcagcggccgcatccctgcACTCTTTTCGGTCATTATCTTG |
| BRO1 R1 | K/O | cacggcgcgcctagcagcggAGGATGTAAGGATAAGTTGGTG |
| BRO1 R3 | K/O | TGAATTTACAGGCGTATATGTG |
| BRO1 nF | K/O | TTGTCGTTATTGGATTTATTGA |
| BRO1 nR | K/O | TTTTCCTCCTGCTATAATGTTT |
| BRO1 intF | K/O | AATGGTTTGATTCATTTTCTTC |
| BRO1 intR | K/O | TTCAGGTAATGTCACCAAGTTA |
| BRO1 K/O Check U | K/O | TCCTTTTCATGATCATTGTT |
| BRO1 K/O Check D | K/O | CCTCCTTATAAACACGTGAA |
| BRO1 Compl F | Compl | ccgctgctaggcgcgccgtgTTGTCGTTATTGGATTTATTGA |
| BRO1 Compl R | Compl | gcagggatgcggccgctgacAAAAAGAATTCAAAACATTGCT |
| BRO1 Compl nF | Compl | GTCATTCAAGCAATTAATGAAA |
| BRO1 Compl nR | Compl | ACTAAAACCCCAACAAATCAC |
| BRO1 Compl Check D F | Compl | TACAATTTTGCCACAACTCTAC |
| BRO1 Compl Check D R | Compl | TTCAGTCAGTTTATTGACAACG |
| BRO1 Compl Check U F | Compl | ttttgattttgaagctagtgtg |
| BRO1 Compl Check U R | Compl | GAAAACAATTTTGGTGAGAAAT |
| DOA4 F1 | K/O | TGTTTTTGTTTTTGAGTGTAGC |
| DOA4 F3 | K/O | gtcagcggccgcatccctgcTCGACTTTATCGTCATGATTTA |
| DOA4 R1 | K/O | cacggcgcgcctagcagcggAGTTGGTGGTAATTTAGTTTGG |
| DOA4 R3 | K/O | TAGTCACACCAAAGAAGAAACA |
| DOA4 nF | K/O | TTTTCAAATTGATATGGATACG |
| DOA4 nR | K/O | TTAGCACATTCGTGAAGAAAC |
| DOA4 intF | K/O | GTATCATTGTCGGAAGAAGAAT |
| DOA4 intR | K/O | CCAAAAGAAAATTCAAAAACTC |
| DOA4 K/O Check U | K/O | AAGTGATTTTCGTTATCGGTAT |
| DOA4 K/O Check D | K/O | CATTACTTGCTTCAATGTGG |
| DOA4 Compl F | Compl | ccgctgctaggcgcgccgtgGCTGCAAAGTTTTCAAATTGAT |
| DOA4 Compl R | Compl | gcagggatgcggccgctgacATTACTTGCTTCAATGTGGGAA |
| DOA4 Compl nF | Compl | AGCAATTAATGAAAGAGTTGACT |
| DOA4 Compl nR | Compl | gtttaaacTCGAAAACGATGTT |
| DOA4 Compl Check D F | Compl | TTGAACCATACTAAATCGGAG |
| DOA4 Compl Check D R | Compl | ACCACCACGACCACTAAAAC |
| DOA4 Compl Check U F | Compl | gaaagaagagatgctattggtg |
| DOA4 Compl Check U R | Compl | GTTATGTTTTCGTGACACTTCT |
| HSE1 F1 | K/O | CACCTAAGATCCTTTGTTTGTT |
| HSE1 F3 | K/O | gtcagcggccgcatccctgcTCTTCAGTTTCTTTTCTCAAGC |
| HSE1 R1 | K/O | cacggcgcgcctagcagcggGAGGAGGCAAAGTAGTTGTATT |
| HSE1 R3 | K/O | CAATTTATGAACATGAACAAGC |
| HSE1 Nf | K/O | AACTGAGTTCACTTCAACAACA |
| HSE1 Nr | K/O | CAATTTATGAACATGAACAAGC |
| HSE1 intF | K/O | AGAGAGCTTTGAAATTGTCATT |
| HSE1 intR | K/O | GTGGGTACTGGTTAAAAGTTGT |
| HSE1 K/O Check U | K/O | TTCCATCTTTGTTGCTATTC |
| HSE1 K/O Check D | K/O | CTGTTAAAGCTGAAAATCCA |
| HSE1 Compl F | Compl | ccgctgctaggcgcgccgtgCACCTAAGATCCTTTGTTTGTT |
| HSE1 Compl R | Compl | gcagggatgcggccgctgacATTGTTGATCCTTCTAAATTGG |
| HSE1 Compl nF | Compl | GTCATTCAAGCAATTAATGAAA |
| HSE1 Compl nR | Compl | ACTAAAACCCCAACAAATCAC |
| HSE1 Compl Check U F | Compl | AGAAGATAAGGCAGACCAAATA |
| HSE1 Compl Check U R | Compl | TATGCAGGATTTTGATAGTCTG |
| HSE1 Compl Check U1 R | Compl | AACGTTACACAACCATTGACTA |
| HSE1 Compl Check D F | Compl | TATAATAGAGCTGCACCTGGAC |
| HSE1 Compl Check D R | Compl | TGAGGATGAAGAGTTTTTCTCT |
| MVB12 F1 | K/O | ATTCAACAGATCCAGGAAGATA |
| MVB12 F3 | K/O | gtcagcggccgcatccctgcAAACTATTGGTGTTCATCTTCC |
| MVB12 R1 | K/O | cacggcgcgcctagcagcggGATTGAAAAGATTTCGCATATT |
| MVB12 R3 | K/O | AATTCTTTCTTTAAGCCATTTTT |
| MVB12 nF | K/O | GACATTCATCAACATCAAAAGA |
| MVB12 nR | K/O | TGGAAATAATCATTACATCGTG |
| MVB12 intF | K/O | CTCTATATTCTAGCGGATCCAA |
| MVB12 intR | K/O | TTAATTTCGTTGTCCCAAATA |
| MVB12 K/O Check U | K/O | ACAATAAAGTATGCAGAATAACAG |
| MVB12 K/O Check D | K/O | CAAAAGGTTCAGTTTTATAACCA |
| MVB12 Compl F | Compl | ccgctgctaggcgcgccgtgAAGAAGAAGAAGAAGAAGGAGG |
| MVB12 Compl R | Compl | gcagggatgcggccgctgacGAAGATTCAGGAATAGTAGTTAAAG |
| MVB12 Compl nF | Compl | AGCAATTAATGAAAGAGTTGACT |
| MVB12 Compl nR | Compl | gtttaaacTCGAAAACGATGTT |
| MVB12 Compl Check D F | Compl | TTGTCAGGTGTTTTGATAATGT |
| MVB12 Compl Check D R | Compl | CTAAAACCCCAACAAATCACAC |
| MVB12 Compl Check U F | Compl | atagaaagataccctgtattcca |
| MVB12 Compl Check U R | Compl | TTTTGCTCTTCCCCTCCTTTT |
| SNF7 F1 | K/O | ACCAGAAAATCTACCATACGAC |
| SNF7 F3 | K/O | gtcagcggccgcatccctgcATTCTTTTTGTTGTTGTTGTTG |
| SNF7 R1 | K/O | cacggcgcgcctagcagcggAAAAAGAAAAGAACCTGGTGTT |
| SNF7 R3 | K/O | TCAGTAGCGTTGACTAACTTTG |
| SNF7 intF | K/O | GATTTACCAAAGAAGGCAATAG |
| SNF7 intR | K/O | AATGCTTCTTCATCTTCATCTT |
| SNF7 nF | K/O | GGAAGAACAACAATATGGAAAT |
| SNF7 nR | K/O | GTATCGATTTGTGATGTAGCTG |
| SNF7 K/O Check U | K/O | TTATCATTACCTTCGCAAAC |
| SNF7 K/O Check D | K/O | TGGTTAATCGACATTAAAGG |
| SNF7 Compl F | Compl | ccgctgctaggcgcgccgtgCAAGGTGAACAAATACAAAGAA |
| SNF7 Compl R | Compl | gcagggatgcggccgctgacCAACAACAACAACAAAAAGAA |
| SNF7 Compl nF | Compl | GAAGTCGACTATGTCATTCAAG |
| SNF7 Compl nR | Compl | ACCACTAAAACCCCAACAAAT |
| SNF7 Compl Check UF | Compl | CCATAAAATATTCGGTTTGATT |
| SNF7 Compl Check U R | Compl | TAATAAAATGCTTTACCGGAAT |
| SNF7 Compl Check D R | Compl | AAAAATATCCCCACATGTTTAC |
| SNF7 Compl Check D F | Compl | ATGAATTTGTTGATGAAGATGA |
| SRN2 F1 | K/O | ATTTGGCTTTGTATGGTTAGAC |
| SRN2 F3 | K/O | gtcagcggccgcatccctgcAACGTTCAGGAACTTTTCTCTA |
| SRN2 R1 | K/O | cacggcgcgcctagcagcggTTTTCCTCCCTTATCTTGTTTA |
| SRN2 R3 | K/O | GTCGAATGAACTAACGTTGTAA |
| SRN2 nF | K/O | AAAGTGTGACTGGTTTGATGTA |
| SRN2 nR | K/O | ATTGAATACTTTCGAGAGATGG |
| SRN2 intF | K/O | ATACACCTTGACCAAACTTCTC |
| SRN2 intR | K/O | TCGATGTAATTTCTCCTTTCTT |
| SRN2 K/O Check U | K/O | ATGGGTTCATGTACTTGATGAT |
| SRN2 K/O Check D | K/O | ATGATGAAACTAGTCGAATGAAC |
| SRN2 Compl F | Compl | ccgctgctaggcgcgccgtgGAGATGTTGTAGTTAATAGAGTCT |
| SRN2 Compl R | Compl | gcagggatgcggccgctgacAACTAGTCGAATGAACTAACGT |
| SRN2 Compl nF | Compl | AGCAATTAATGAAAGAGTTGACT |
| SRN2 Compl nR | Compl | gtttaaacTCGAAAACGATGTT |
| SRN2 Compl Check U F | Compl | gtattccattgtatcgcctttg |
| SRN2 Compl Check U R | Compl | TGATCTATAATTTCCAGCTGCT |
| SRN2 Compl Check D F | Compl | TTGGTGTAGTAGATTGTAGGTG |
| SRN2 Compl Check D R | Compl | CTAAAACCCCAACAAATCACAC |
| SRN2 Compl Check D2 F | Compl | GAACAGTTGAGGATGAAGTTTT |
| SRN2 Compl Check D2 R | Compl | AAGCAACCTTTATTGAGTGAAG |
| SRN2 Compl Check D3 F | Compl | TAACTCCCTTCTCGGTTTTATT |
| SRN2 Compl Check D3 R | Compl | ATATACGAGAGCCAAGTCAATC |
| VPS2 F1 | K/O | ATGGAATTTCAATTGTCCTAAA |
| VPS2 F3 | K/O | gtcagcggccgcatccctgcCTATAATTTGTTCAGCAGCTTG |
| VPS2 R1 | K/O | cacggcgcgcctagcagcggATTTACCTTTGAGCTTTTGTTG |
| VPS2 R3 | K/O | TTGCAAATAAACCTCTATCACA |
| VPS2 nF | K/O | TTCATTCGTAAGAAATTGACAC |
| VPS2 nR | K/O | ATGACAAATTGCAACAAAATTA |
| VPS2 intF | K/O | TGGTAAGAAGTTAACACCACAA |
| VPS2 intR | K/O | AACTATCTAATCGCGCTTGTAA |
| VPS2 K/O Check U | K/O | AACCTTCACAAACTAAAATTGAA |
| VPS2 K/O Check D | K/O | CGATTTTGACCTCAAATCATA |
| VPS2 Compl F | Compl | ccgctgctaggcgcgccgtgTCGACATCTCTCAGTAAATCATAT |
| VPS2 Compl R | Compl | gcagggatgcggccgctgacTAGAGTTACAATTGAGCTCCAC |
| VPS2 Compl nF | Compl | AGCAATTAATGAAAGAGTTGACT |
| VPS2 Compl nR | Compl | gtttaaacTCGAAAACGATGTT |
| VPS2 Compl Check D F | Compl | GAAGTGACAGTGATACAACATG |
| VPS2 Compl Check D R | Compl | CAGTAGTGAGGATGAAGAGTTT |
| VPS2 Compl Check U F | Compl | atttctttcaccaatcaactcg |
| VPS2 Compl Check U R | Compl | CCTAATACTCTAGTGGCATCTC |
| VPS20 F1 | K/O | GGCTATTTCTAACACAAAAACC |
| VPS20 F3 | K/O | gtcagcggccgcatccctgcTTGCATAGTTTCTAGTTCAACG |
| VPS20 R1 | K/O | cacggcgcgcctagcagcggACAGTTCCTGATAACTCGTAGG |
| VPS20 R3 | K/O | AAGCGTTTTGGTGTAAATAAAT |
| VPS20 nF | K/O | TTTCCGATACCCTTAATAAAAA |
| VPS20 nR | K/O | AGCTGTGATATTGTTTGGAGTT |
| VPS20 intF | K/O | TAAAATAACTGCACAAGACAGG |
| VPS20 intR | K/O | TGTTCTTCGATTTCATTACTTG |
| VPS20 K/O Check U | K/O | TTAATTTTTACGGTTGCTTTTT |
| VPS20 K/O Check D | K/O | TATGTGAAAAACTTGGTTGAAA |
| VPS20 Compl F | Compl | ccgctgctaggcgcgccgtg GATAATTCATGAAGGTTTATCGATG |
| VPS20 Compl R | Compl | gcagggatgcggccgctgac GCCTTCCTGATTTTGCAAGA |
| VPS20 Compl nF | Compl | AGCAATTAATGAAAGAGTTGACT |
| VPS20 Compl nR | Compl | gtttaaacTCGAAAACGATGTT |
| VPS20 Compl Check D F | Compl | ATAATGAGATGCAAGTCGAAAA |
| VPS20 Compl Check D R | Compl | CTAAAACCCCAACAAATCACAC |
| VPS20 Compl Check U F | Compl | taccctgtattccattgtatcg |
| VPS20 Compl Check U R | Compl | ATCCAGCCTCTTAGTAATCAAG |
| VPS22 F1 | K/O | AACTTGAGTCAGAGGAATTGAA |
| VPS22 F3 | K/O | gtcagcggccgcatccctgcTTTTTCCATCCATCTTAGATTC |
| VPS22 R1 | K/O | cacggcgcgcctagcagcggTAATCAAAATCAGAAAGGAAGG |
| VPS22 R3 | K/O | GTGGAAGAACTGATAAAGGTGT |
| VPS22 Nf | K/O | ATCTTAAATTAACTGCTGAACG |
| VPS22 Nr | K/O | TTAAATGCAAATTTACCAAGTG |
| VPS22 intF | K/O | TCACTCAAATTGTTCAACTGAT |
| VPS22 intR | K/O | GACAGTTTTACATCGCACTTTA |
| VPS22 K/O Check U | K/O | TCACTGGTAAAAGGAGTTAAATG |
| VPS22 K/O Check D | K/O | AAGTCAAATTAATCAAAGAACCA |
| VPS22 Compl F | Compl | ccgctgctaggcgcgccgtgGCCATCATTGTAACAAAACCAA |
| VPS22 Compl R | Compl | gcagggatgcggccgctgacTGAAATTTTCTCGATGTGGAAG |
| VPS22 Compl nF | Compl | AGCAATTAATGAAAGAGTTGACT |
| VPS22 Compl nR | Compl | gtttaaacTCGAAAACGATGTT |
| VPS22 Compl Check U F | Compl | aagataccctgtattccattgt |
| VPS22 Compl Check U R | Compl | TCACTCATTTATGGTTGTCAAC |
| VPS22 Compl Check D F | Compl | TCACAATCATACAACACCAATG |
| VPS22 Compl Check D R | Compl | CAATACCAGCGCTATAACATTG |
| VPS22 Compl Check D2 F | Compl | GTAGTAGTGGTTGGATCTTGAT |
| VPS22 Compl Check D2 R | Compl | AAATCATATACGAGAGCCAAGT |
| VPS22 Compl Check D3 F | Compl | CTTCCATAATTTGCAGCATTTG |
| VPS22 Compl Check D3 R | Compl | TGAAGAGCCAAATCATATACGA |
| VPS23 F1 | K/O | AGGTTTAATGCTTTTTGGAATA |
| VPS23 F3 | K/O | gtcagcggccgcatccctgcTTATTAATAGGAAGGGGCTGTA |
| VPS23 R1 | K/O | cacggcgcgcctagcagcggTTTTTAGTTATGTGGGTGTTTG |
| VPS23 R3 | K/O | CCAGAATCAACAGTTTACATCA |
| VPS23 nF | K/O | TCCTTAACAGAACCCATAATTC |
| VPS23 nR | K/O | CATAGCTAGTGAAAAACGTCAA |
| VPS23 intF | K/O | CATGTTCAGTTTGGTAGAATTG |
| VPS23 intR | K/O | TATTAATTTCGGCACTAACCTT |
| VPS23 K/O Check U | K/O | TCGAACAATAAACACAACAA |
| VPS23 K/O Check D | K/O | CAAAATATTACCCCTCCAAT |
| VPS23 Compl F | Compl | ccgctgctaggcgcgccgtgATTTCGAACAATAAACACAACA |
| VPS23 Compl R | Compl | gcagggatgcggccgctgacCATAGCTAGTGAAAAACGTCAA |
| VPS23 Compl nF | Compl | GTCATTCAAGCAATTAATGAAA |
| VPS23 Compl nR | Compl | AAACGATGTTTGCACCAC |
| VPS23 Compl Check U F | Compl | CCATAAAATATTCGGTTTGATT |
| VPS23 Compl Check D R | Compl | CAATAGATTAGGCTCTCCTGAC |
| VPS23 Compl Check D F | Compl | CTACTTCAAACAGACCTGTCCT |
| VPS23 Compl Check U R | Compl | TTTTTGATGTGTCTTTTGATGT |
| VPS24 F1 | K/O | CAACAATGTCGTCATAACTAGG |
| VPS24 F3 | K/O | gtcagcggccgcatccctgcAAATATAGAGAAATGCCCAAAA |
| VPS24 R1 | K/O | cacggcgcgcctagcagcggGGTTTATTTCTTGTTCAATGGT |
| VPS24 R3 | K/O | ATTTGGTTCTCTTATCCCCTAT |
| VPS24 nF | K/O | ATACGAATACCTTGTTGTTGCT |
| VPS24 nR | K/O | TCAATTTCATTCTTCTTTCTTTC |
| VPS24 intF | K/O | CAAAGAACAGGTATGTGAAAAA |
| VPS24 intR | K/O | ATTTCATCTAATGCCAATTCA |
| VPS24 K/O Check U | K/O | AGTCAAGATTTGATTCTCCTTG |
| VPS24 K/O Check D | K/O | GAATCTGGATTTGGTTCTCTT |
| VPS24 Compl F | Compl | ccgctgctaggcgcgccgtgAGGGACAATATAATAGAACTGGT |
| VPS24 Compl R | Compl | gcagggatgcggccgctgacTCATTATTGTCATAACCATTGG |
| VPS24 Compl nF | Compl | AGCAATTAATGAAAGAGTTGACT |
| VPS24 Compl nR | Compl | gtttaaacTCGAAAACGATGTT |
| VPS24 Compl Check U F | Compl | taccctgtattccattgtatcg |
| VPS24 Compl Check U R | Compl | AAGAGAAAACTTGCAAATCGAT |
| VPS24 Compl Check U2 F | Compl | gaaagaagagatgctattggtg |
| VPS24 Compl Check U2 R | Compl | GTGGAAAAGATGAAAAGACGAT |
| VPS24 Compl Check U3 F | Compl | gtgaatgtgttagaaaagctga |
| VPS24 Compl Check U3 R | Compl | GCAATAAATCACTGGTCATACA |
| VPS24 Compl Check D F | Compl | GAAACTTCATGGGCAATTGAA |
| VPS24 Compl Check D R | Compl | CAATACCAGCGCTATAACATTG |
| VPS25 F1 | K/O | AGTTTGGATGAATAGAAAGCAT |
| VPS25 F3 | K/O | gtcagcggccgcatccctgcTTGATGTTTACGCTAGTCAAAG |
| VPS25 R1 | K/O | cacggcgcgcctagcagcggCAGCAAGAGAAATGAGATTACA |
| VPS25 R3 | K/O | TTTTCAATAACATGCGAATAGA |
| VPS25 nF | K/O | AAGGAATGGAAGTATCAATTTT |
| VPS25 nR | K/O | AGGGTCAATTTTGATTGAAGTA |
| VPS25 intF | K/O | TACTCATTTCCACCATTTTACA |
| VPS25 intR | K/O | TCGTTATTCTCGTCTATCAACA |
| VPS25 K/O Check U | K/O | GAATCAAGAGATAAGAGGAGTCA |
| VPS25 K/O Check D | K/O | AACGTTCATAATTACCCAAATC |
| VPS25 Compl F | Compl | ccgctgctaggcgcgccgtgAATGTGAAATTAGGGTGAGAAT |
| VPS25 Compl R | Compl | gcagggatgcggccgctgacCTTGCTAATAGAGAGATGGAGG |
| VPS25 Compl nF | Compl | AGCAATTAATGAAAGAGTTGACT |
| VPS25 Compl nR | Compl | gtttaaacTCGAAAACGATGTT |
| VPS25 Compl Check U F | Compl | taccctgtattccattgtatcg |
| VPS25 Compl Check U R | Compl | AGTGAAAGTGGAACCTCTAATT |
| VPS25 Compl Check D F | Compl | AGCCAACACATATATAGAGCAA |
| VPS25 Compl Check D R | Compl | CTAAAACCCCAACAAATCACAC |
| VPS27 F1 | K/O | GTTATTGCGCTAAGTTCTTCTT |
| VPS27 F3 | K/O | gtcagcggccgcatccctgcTTTGCTTATCTAGAAATATTTAGCC |
| VPS27 R1 | K/O | cacggcgcgcctagcagcggATTTGAATTTGGAGGTTTGATA |
| VPS27 R3 | K/O | TCAGAATGTGATTTTTAATGGA |
| VPS27 nF | K/O | TGTCAATGTCTGGATGAGTATC |
| VPS27 nR | K/O | GGTTTTAGTGTTTGGTGAGATT |
| VPS27 intF | K/O | ATCACAAGATTTATCACAAGCA |
| VPS27 intR | K/O | GGAGGATAATGAGGTAATGAAA |
| VPS27 K/O Check U | K/O | GGCACTCAAACTCTCAAGTA |
| VPS27 K/O Check D | K/O | AATGAATCTTCATCATTTGG |
| VPS27 Compl F | Compl | ccgctgctaggcgcgccgtgGGTTGGTTGGTAAGGATATTAG |
| VPS27 Compl R | Compl | gcagggatgcggccgctgacTATAAACCACGACAAACCTACA |
| VPS27 Compl nF | Compl | CAAGCAATTAATGAAAGAGTTG |
| VPS27 Compl nR | Compl | ACTAAAACCCCAACAAATCAC |
| VPS27 Compl Check D F | Compl | TTCGGTTTGATTAGGTTATTTT |
| VPS27 Compl Check U R | Compl | TAATGATCTCATGGCAATTTTA |
| VPS27 Compl Check U R | Compl | ATCGTCTGGAGATTAAGAAGAA |
| VPS27 Compl Check D F | Compl | TCAGAGTAGCACATAATCGAAC |
| VPS28 F1 | K/O | GAATTTGGGAAAGAATTCAATA |
| VPS28 F3 | K/O | gtcagcggccgcatccctgcTCTGTTTCTTGCTTATACGATG |
| VPS28 R1 | K/O | cacggcgcgcctagcagcggTTTATTGTTCGATTTTACGATG |
| VPS28 R3 | K/O | ATCTTCACATTCCTTTCAACTC |
| VPS28 nF | K/O | AAACTAATAGACCGTTTTCGAC |
| VPS28 nR | K/O | ACTTGCTCCGATTAAACTAGAA |
| VPS28 intF | K/O | AACCAAGAAGTTACCAAATCAC |
| VPS28 intR | K/O | ATCAGCTTCCTCTTGTGTAAGT |
| VPS28 K/O Check U | K/O | CAACCTCAAAGTATCTGGAAAT |
| VPS28 K/O Check D | K/O | TGTCTTTACAAAAACTCACTGC |
| VPS28 Compl F | Compl | ccgctgctaggcgcgccgtgAGACGATATTGTTTAAACTAGCA |
| VPS28 Compl R | Compl | gcagggatgcggccgctgacGTTCTCTCTTTCTTTCTTGGTG |
| VPS28 Compl nF | Compl | AGCAATTAATGAAAGAGTTGACT |
| VPS28 Compl nR | Compl | gtttaaacTCGAAAACGATGTT |
| VPS28 Compl Check U F | Compl | gattttgaagctagtgtggaaa |
| VPS28 Compl Check U R | Compl | GTAACTTCTTGGTTGTAAACGT |
| VPS28 Compl Check D F | Compl | TCAAACCCTGACTCAACAAG |
| VPS28 Compl Check D R | Compl | CTAAAACCCCAACAAATCACAC |
| VPS36 F1 | K/O | TTTTGAATGCACTAAGTAATCG |
| VPS36 F3 | K/O | gtcagcggccgcatccctgcTATGCTTATGTTGTCTTTTTGC |
| VPS36 R1 | K/O | cacggcgcgcctagcagcggTTTGCTTCTTGTCTCTCTTTTT |
| VPS36 R3 | K/O | ATGAATACTATTGTTCCCCTTG |
| VPS36 nF | K/O | AGTCGGTCGAGTATATCTCTTG |
| VPS36 nR | K/O | AGTGGTGTTGATCATGATACTG |
| VPS36 intF | K/O | TGATATCAAATGGCAAATTCTA |
| VPS36 intR | K/O | TGTTTATCGTATTCATTCCTCA |
| VPS36 K/O Check U | K/O | ATAACAACATCTTTCCGTGAAT |
| VPS36 K/O Check D | K/O | ATGATGATGATTGTCACTTTTG |
| VPS36 Compl F | Compl | ccgctgctaggcgcgccgtgATTGAAATGATTATAAGCGGGT |
| VPS36 Compl R | Compl | gcagggatgcggccgctgacTTGCATTGGGTTTTCTATATGT |
| VPS36 Compl nF | Compl | AGCAATTAATGAAAGAGTTGACT |
| VPS36 Compl nR | Compl | gtttaaacTCGAAAACGATGTT |
| VPS36 Compl Check U F | Compl | taccctgtattccattgtatcg |
| VPS36 Compl Check U R | Compl | TTTTGCTTCTTGTCTCTCTTTT |
| VPS36 Compl Check D F | Compl | CCTGATTATAGTTTCTTTGGTGC |
| VPS36 Compl Check D R | Compl | CCACTAAAACCCCAACAAATC |
| VPS36 Compl Check D2 F | Compl | ATTGGAAAAGTGTGTTGAAGAG |
| VPS36 Compl Check D2 R | Compl | AAATCATATACGAGAGCCAAGT |
| VPS36 Compl Check D3 F | Compl | TTGATTAAGGAACAACAGGAGA |
| VPS36 Compl Check D3 R | Compl | TCGGAAATGACAAATGAATTCA |
| VPS4 F1 | K/O | TCAAATCTCAACGCAAGTATAG |
| VPS4 F3 | K/O | gtcagcggccgcatccctgcACAATTGAAACAAACCATTGTA |
| VPS4 R1 | K/O | cacggcgcgcctagcagcggAAAAATTTCCTCCATATTGTTG |
| VPS4 R3 | K/O | CACGATAATAAACCCTGAAACT |
| VPS4 nF | K/O | ATTATTTTAACCGCATTTCATC |
| VPS4 nR | K/O | GACTGAAGAACTGGATAGTGGT |
| VPS4 intF | K/O | ATGATAATGATGATGCTGACAC |
| VPS4 intR | K/O | TTCATTAACTGTTGGTCGATTA |
| VPS4 K/O Check U | K/O | AGTTGCTGGTTCAATTTATG |
| VPS4 K/O Check D | K/O | AAAAGATCAGTGAATCACCA |
| VPS4 Compl F | Compl | ccgctgctaggcgcgccgtgTTCTTGGAGAGAGAGAAACATT |
| VPS4 Compl R | Compl | gcagggatgcggccgctgacAAAGGGTGAAAGACAAAGTA |
| VPS4 Compl nF | Compl | GAAGTCGACTATGTCATTCAAG |
| VPS4 Compl nR | Compl | ACTAAAACCCCAACAAATCAC |
| VPS4 Compl Check U F | Compl | CCATAAAATATTCGGTTTGATT |
| VPS4 Compl Check U R | Compl | GTCGATCCATTTACAGAACTTT |
| VPS4 Compl Check D F | Compl | TATAGGCTTCTTCATACCGAGT |
| VPS4 Compl Check D R | Compl | GGAAAGAAAAATTAACACCTTG |
| SUN41 F1 | K/O | AAGATAGCATTCAACATGACAA |
| SUN41 R1 | K/O | cacggcgcgcctagcagcggAAAGGAACGACTAAAAGAAACA |
| SUN41 F3 | K/O | gtcagcggccgcatccctgcACAAGATACCCCTTTTTCTCTT |
| SUN41 R3 | K/O | TCATTGTCACAACCATTATCTC |
| SUN41 nF | K/O | ATATCAATTTTTATTGGGCAAC |
| SUN41 nR | K/O | TATTATTTACTGCTGCATTTGG |
| SUN41 intF | K/O | TCCTATCACTACTGTCAGTCCA |
| SUN41 intR | K/O | CAAGTAAGCAATACCATTAGCA |
| SUN41 K/O Check U | K/O | ACTACCAAGCAAAACATCTACC |
| SUN41 K/O Check D | K/O | CCTTAGCACTACTAAAGCTGGT |
| SUN41 Compl F | Compl | ccgctgctaggcgcgccgtgAGATAGCATTCAACATGACAAA |
| SUN41 Compl R | Compl | gcagggatgcggccgctgacCGTCATTGTCACAACCATTATC |
| SUN41 Compl nF | Compl | TCAAGCAATTAATGAAAGAGTTG |
| SUN41 Compl nR | Compl | ggggatcgtttaaacTCG |
| SUN41 Compl Check U F | Compl | gtattccattgtatcgcctttg |
| SUN41 Compl Check U R | Compl | GAAAGAATTCCTGCATGTAACT |
| SUN41 Compl Check D F | Compl | GGCACATCAAATTCGATATCAT |
| SUN41 Compl Check D R | Compl | TATAACATTGACGAGCAGTAGT |

1 K/O – knockout; Compl - complement
