# Supplementary material for: Candida albicans biofilm–induced vesicles confer drug resistance through matrix biogenesis
Source: PLoS Biol. 2018 Oct 8;16(10):e2006872. doi: 10.1371/journal.pbio.2006872 (PMC6209495; doi:10.1371/journal.pbio.2006872)
Supplement: S6 Table — (DOCX) [file pbio.2006872.s007.docx]

**S6 Table. Strain genotypes**

| **Gene** | **Strain** | **Genotype** | **Ref** |
| --- | --- | --- | --- |
| Reference | SN152 | *URA3 IRO1 arg4 his1 leu2*  *ura3::λimm434 iro1::* λimm434 *arg4 his1 leu2* | ^1^ |
| *bro1 -/-* | URZ370 | *URA3 IRO1 arg4 his1 leu2 bro1::C.d HIS1*  *ura3::λimm434 iro1::* λimm434 *arg4 his1 leu2 bro1::C.m LEU2* | This study |
| *bro1 -/-, +* | URZ430 | *URA3 IRO1 arg4 his1 leu2::BRO1::Nou^R^ bro1::C.d HIS1*  *ura3::λimm434 iro1::* λimm434 *arg4 his1 leu2 bro1::C.m LEU2* | This study |
| *doa4 -/-* | URZ468 | *URA3 IRO1 arg4 his1 leu2 doa4::C.d HIS1*  *ura3::λimm434 iro1::* λimm434 *arg4 his1 leu2 doa4::C.m LEU2* | This study |
| *doa4 -/-, +* | URZ531 | *URA3 IRO1 arg4 his1 leu2::DOA4::Nou^R^ doa4::C.d HIS1*  *ura3::λimm434 iro1::* λimm434 *arg4 his1 leu2 doa4::C.m LEU2* | This study |
| *hse1 -/-* | URZ358 | *URA3 IRO1 arg4 his1 leu2 hse1::C.d HIS1*  *ura3::λimm434 iro1::* λimm434 *arg4 his1 leu2 hse1::C.m LEU2* | This study |
| *hse1 -/-, +* | URZ403 | *URA3 IRO1 arg4 his1 leu2::HSE1::Nou^R^ hse1::C.d HIS1*  *ura3::λimm434 iro1::* λimm434 *arg4 his1 leu2 hse1::C.m LEU2* | This study |
| *mvb12 -/-* | URZ477 | *URA3 IRO1 arg4 his1 leu2 mvb12::C.d HIS1*  *ura3::λimm434 iro1::* λimm434 *arg4 his1 leu2 mvb12::C.m LEU2* | This study |
| *vps2 -/-* | URZ453 | *URA3 IRO1 arg4 his1 leu2 vps2::C.d HIS1*  *ura3::λimm434 iro1::* λimm434 *arg4 his1 leu2 vps2::C.m LEU2* | This study |
| *vps4 -/-* | URZ368 | *URA3 IRO1 arg4 his1 leu2 vps4::C.d HIS1*  *ura3::λimm434 iro1::* λimm434 *arg4 his1 leu2 vps4::C.m LEU2* | This study |
| *vps4 -/-, +* | URZ400 | *URA3 IRO1 arg4 his1 leu2::VPS4::Nou^R^ vps4::C.d HIS1*  *ura3::λimm434 iro1::* λimm434 *arg4 his1 leu2 vps4::C.m LEU2* | This study |
| *vps20 -/-* | URZ458 | *URA3 IRO1 arg4 his1 leu2 vps20::C.d HIS1*  *ura3::λimm434 iro1::* λimm434 *arg4 his1 leu2 vps20::C.m LEU2* | This study |
| *vps20 -/-, +* | URZ537 | *URA3 IRO1 arg4 his1 leu2::VPS20::Nou^R^ vps20::C.d HIS1*  *ura3::λimm434 iro1::* λimm434 *arg4 his1 leu2 vps20::C.m LEU2* | This study |
| *vps22 -/-, +* | URZ501 | *URA3 IRO1 arg4 his1 leu2 vps22::C.d HIS1*  *ura3::λimm434 iro1::* λimm434 *arg4 his1 leu2 vps22::C.m LEU2* | This study |
| *vps23 -/-* | URZ364 | *URA3 IRO1 arg4 his1 leu2 vps23::C.d HIS1*  *ura3::λimm434 iro1::* λimm434 *arg4 his1 leu2 vps23::C.m LEU2* | This study |
| *vps23 -/-, +* | URZ418 | *URA3 IRO1 arg4 his1 leu2::VPS23::Nou^R^ vps23::C.d HIS1*  *ura3::λimm434 iro1::* λimm434 *arg4 his1 leu2 vps23::C.m LEU2* | This study |
| *vps24 -/-* | URZ470 | *URA3 IRO1 arg4 his1 leu2 vps24::C.d HIS1*  *ura3::λimm434 iro1::* λimm434 *arg4 his1 leu2 vps24::C.m LEU2* | This study |
| *vps25 -/-* | URZ503 | *URA3 IRO1 arg4 his1 leu2 vps25::C.d HIS1*  *ura3::λimm434 iro1::* λimm434 *arg4 his1 leu2 vps25::C.m LEU2* | This study |
| *vps25 -/-, +* | URZ534 | *URA3 IRO1 arg4 his1 leu2::VPS25::Nou^R^ vps25::C.d HIS1*  *ura3::λimm434 iro1::* λimm434 *arg4 his1 leu2 vps25::C.m LEU2* | This study |
| *vps27 -/-* | URZ362 | *URA3 IRO1 arg4 his1 leu2 vps27::C.d HIS1*  *ura3::λimm434 iro1::* λimm434 *arg4 his1 leu2 vps27::C.m LEU2* | This study |
| *vps27 -/-, +* | URZ398 | *URA3 IRO1 arg4 his1 leu2::VPS27::Nou^R^ vps27::C.d HIS1*  *ura3::λimm434 iro1::* λimm434 *arg4 his1 leu2 vps27::C.m LEU2* | This study |
| *vps28 -/-* | URZ461 | *URA3 IRO1 arg4 his1 leu2 vps28::C.d HIS1*  *ura3::λimm434 iro1::* λimm434 *arg4 his1 leu2 vps28::C.m LEU2* | This study |
| *Vps36 -/-* | URZ465 | *URA3 IRO1 arg4 his1 leu2 vps36::C.d HIS1*  *ura3::λimm434 iro1::* λimm434 *arg4 his1 leu2 vps36::C.m LEU2* | This study |
| *Vps36 -/-, +* | URZ398 | *URA3 IRO1 arg4 his1 leu2::VPS36::Nou^R^ vps36:C.d HIS1*  *ura3::λimm434 iro1::* λimm434 *arg4 his1 leu2 vps36::C.m LEU2* | This study |
| *snf7 -/-* | URZ362 | *URA3 IRO1 arg4 his1 leu2 snf7::C.d HIS1*  *ura3::λimm434 iro1::* λimm434 *arg4 his1 leu2 snf7::C.m LEU2* | This study |
| *snf7 -/-, +* | URZ403 | *URA3 IRO1 arg4 his1 leu2::SNF7::Nou^R^ snf7::C.d HIS1*  *ura3::λimm434 iro1::* λimm434 *arg4 his1 leu2 snf7::C.m LEU2* | This study |
| *srn2 -/-* | URZ482 | *URA3 IRO1 arg4 his1 leu2 srn2::C.d HIS1*  *ura3::λimm434 iro1::* λimm434 *arg4 his1 leu2 srn2::C.m LEU2* | This study |
| *vta1 -/-* | URZ524 | *URA3 IRO1 arg4 his1 leu2 vta1::C.d HIS1*  *ura3::λimm434 iro1::* λimm434 *arg4 his1 leu2 vta1::C.m LEU2* | This study |
| *sun41 -/-* | URZ376 | *URA3 IRO1 arg4 his1 leu2 sun41::C.d HIS1*  *ura3::λimm434 iro1::* λimm434 *arg4 his1 leu2 sun41::C.m LEU2* | This study |
| *sun41 -/-, +* | URZ562 | *URA3 IRO1 arg4 his1 leu2::SUN41::Nou^R^ sun41::C.d HIS1*  *ura3::λimm434 iro1::* λimm434 *arg4 his1 leu2 sun41::C.m LEU2* | This study |
| *phr1 -/-* | KMR101 | *URA3 IRO1 arg4 his1 leu2 phr1::C.d HIS1*  *ura3::λimm434 iro1::* λimm434 *arg4 his1 leu2 phr1::C.m LEU2* | ^2^ |
| *mnn9 -/-* | KMR392 | *URA3 IRO1 arg4 his1 leu2 mnn9::C.d HIS1*  *ura3::λimm434 iro1::* λimm434 *arg4 his1 leu2 mnn9::C.m LEU2* | ^3^ |

1 Noble, S. M. & Johnson, A. D. Strains and strategies for large-scale gene deletion studies of the diploid human fungal pathogen Candida albicans. *Eukaryot Cell* **4**, 298-309, doi:10.1128/EC.4.2.298-309.2005 (2005).

2 Mitchell, K. F. *et al.* Community participation in biofilm matrix assembly and function. *Proc Natl Acad Sci U S A* **112**, 4092-4097, doi:10.1073/pnas.1421437112 (2015).

3 Taff, H. T. *et al.* A Candida biofilm-induced pathway for matrix glucan delivery: implications for drug resistance. *PLoS Pathog* **8**, e1002848, doi:10.1371/journal.ppat.1002848 (2012).
